# Supplementary material for: Bi‐allelic mutation in SEC16B alters collagen trafficking and increases ER stress
Source: EMBO Mol Med. 2023 Mar 14;15(4):e16834. doi: 10.15252/emmm.202216834 (PMC10086588; doi:10.15252/emmm.202216834)
Supplement: Supplementary file 1 — Expanded View Figures PDF [file EMMM-15-e16834-s009.pdf]

Expanded View Figures

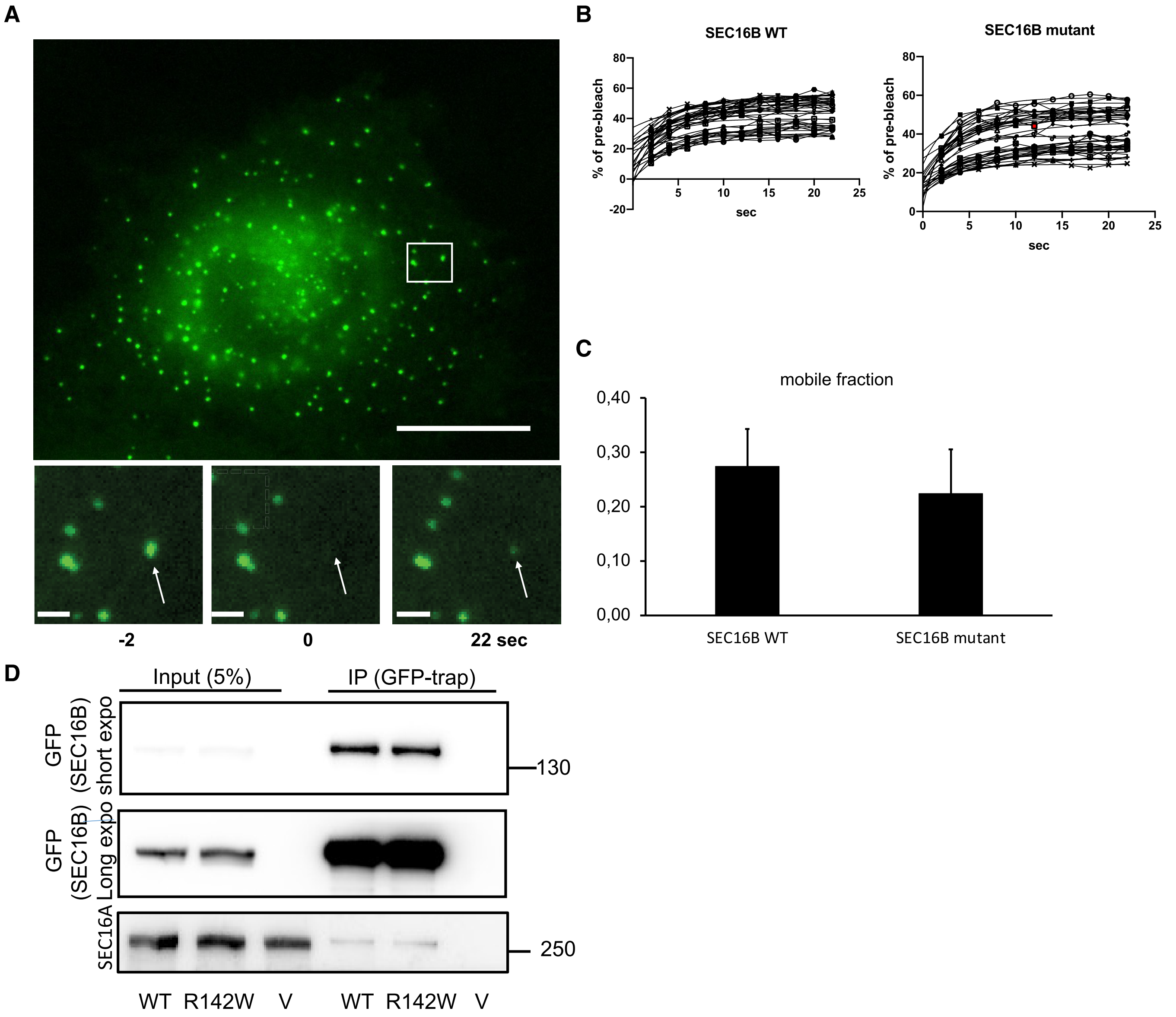

Figure EV1. Characterization of dynamics and interaction of SEC16B mutant.

A HeLa cells were transfected with plasmids encoding wild-type and mutant GFP-SEC16B. After 24 h, FRAP experiment on single ERES was performed on a Nikon CREST V3 spinning disc microscope. The panel shows a representative cell expressing mutant SEC16B with enlarged ERES before and after bleaching. The arrows indicate the position of the bleached ERES. Scale bar in large figure is 10 µm. Scale bar in inserts is 1 µm.

B Quantification of average fluorescence intensities of single ERES. All values were normalized to the pre-bleach fluorescence intensity.

C Calculation of the mobile fractions from the FRAP measurements. Three independent biological experiments. Bars are mean ± SD.

D HeLa cells were transfected with GFP-tagged wild-type or mutant Sec16B. After 24 h, cells were lysed and the lysate was subjected to immunoprecipitation using GFP-tap beads (Chromotek). The immunoprecipitate was immunoblotted against GFP to determine efficiency of the IP, as well as against endogenous Sec16A.

Source data are available online for this figure.

© 2023 The Authors

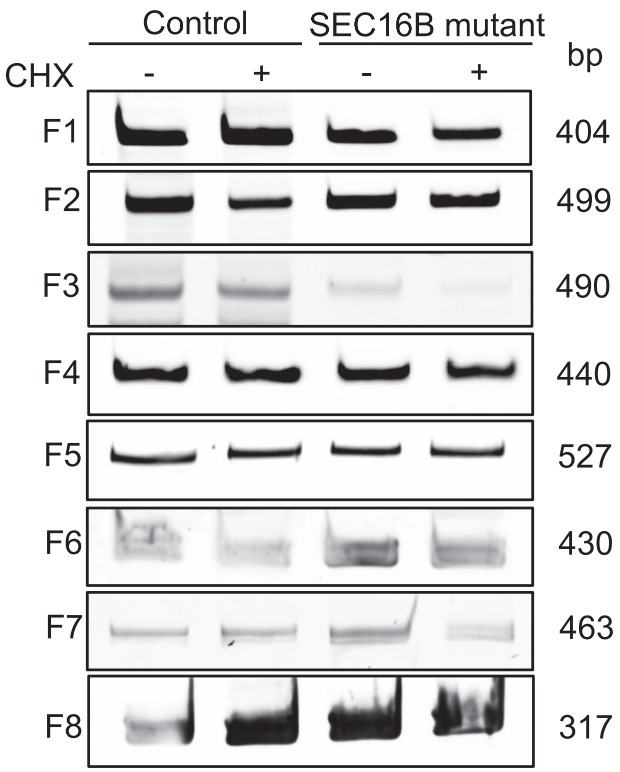

Figure EV2. No evidence of cryptic splicing site in SEC16B open reading frame.

Cells were either left untreated or treated with cycloheximide at concentration of 100 μg/ml for 6 h. gDNA was isolated and PCR was performed using the primer in Table EV4 and sequenced by sanger sequencing. In addition, PCR products were separated on 10% PCR polyacrylamide gel electrophoresis. One representative experiment out of three independent experiments is shown. Biological replicates were performed.

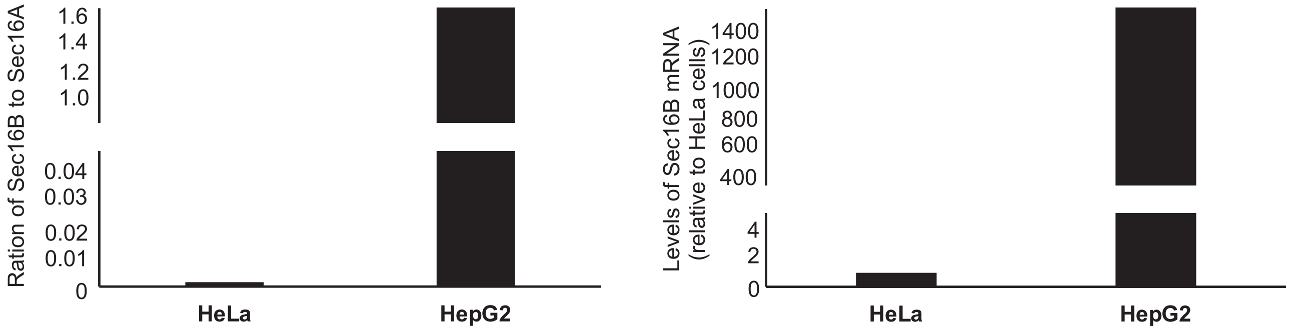

Figure EV3. Relative expression of SEC16A and SEC16B in HeLa and HepG2 cells.

qPCR assessment of mRNA expression level of SEC16B A and SEC16B in HeLa and HepG2 cells. The values are $\Delta\Delta C_T$. HepG2 normalized to HeLa cells. GAPDH was used as housekeeping gene. Bars are from two biological replicates.

 © 2023 The Authors
