# Supplementary material for: Bi‐allelic mutation in SEC16B alters collagen trafficking and increases ER stress
Source: EMBO Mol Med. 2023 Mar 14;15(4):e16834. doi: 10.15252/emmm.202216834 (PMC10086588; doi:10.15252/emmm.202216834)
Supplement: Supplementary file 7 — PDF+ [file EMMM-15-e16834-s003.pdf]

Article

SOURCE DATA · TRANSPARENT PROCESS · OPEN ACCESS

EMBO Molecular Medicine

Bi-allelic mutation in SEC16B alters collagen trafficking and increases ER stress

Ahmed El-Gazzar[1], Barbara Voraberger[1], Frank Rauch[2,3], Mario Mairhofer[4,†], Katy Schmidt[5], Brecht Guillemyn[6], Goran Mitulović[7,‡], Veronika Reiterer[8], Margot Haun[8], Michaela M Mayr[8], Johannes A Mayr[9], Susanne Kimeswenger[10], Oliver Drews[11], Vrinda Saraff[12,13], Nick Shaw[12,14], Nadja Fratzl-Zelman[15,16], Sofie Symoens[6], Hesso Farhan[8] & Wolfgang Högler[1,12,14,*]

Abstract

Osteogenesis imperfecta (OI) is a genetically and clinically heterogeneous disorder characterized by bone fragility and reduced bone mass generally caused by defects in type I collagen structure or defects in proteins interacting with collagen processing. We identified a homozygous missense mutation in *SEC16B* in a child with vertebral fractures, leg bowing, short stature, muscular hypotonia, and bone densitometric and histomorphometric features in keeping with OI with distinct ultrastructural features. In line with the putative function of SEC16B as a regulator of trafficking between the ER and the Golgi complex, we showed that patient fibroblasts accumulated type I procollagen in the ER and exhibited a general trafficking defect at the level of the ER. Consequently, patient fibroblasts exhibited ER stress, enhanced autophagosome formation, and higher levels of apoptosis. Transfection of wild-type *SEC16B* into patient cells rescued the collagen trafficking. Mechanistically, we show that the defect is a consequence of reduced SEC16B expression, rather than due to alterations in protein function. These data suggest *SEC16B* as a recessive candidate gene for OI.

Keywords autophagy; endoplasmic reticulum; osteogenesis imperfecta; SEC16B; type I collagen

Subject Categories Genetics, Gene Therapy & Genetic Disease; Musculoskeletal System

DOI 10.15252/emmm.202216834 | Received 2 September 2022 | Revised 15 February 2023 | Accepted 17 February 2023 | Published online 14 March 2023

EMBO Mol Med (2023) 15: e16834

Introduction

Osteogenesis imperfecta (OI) is a clinically and genetically heterogeneous group of disorders characterized by low bone mass, recurrent fractures, and skeletal deformities, and, in some, blue sclerae, ligamentous laxity, hearing loss, and dentinogenesis imperfecta. About 90% of individuals with OI harbor heterozygous pathogenic variants in the genes that encode the chains of type I collagen (*COL1A1* and *COL1A2*; Van Dijk & Sillence, 2014). Type I collagen is the major protein of the extracellular matrix (ECM) of bone tissue and comprises two α1(I) (COL1A1) and one α2(I) (COL1A2) chains, which form a heterotrimer. Most of the remaining affected individuals develop the condition as a result of variants in over 20 other genes (El-Gazzar & Hogler, 2021). These genes encode proteins that are involved in bone development, post-translational modification of the chains of type I procollagen, assembly of procollagen molecules, secretion of the proteins, assembly of proteins into fibril structures, conversion of procollagen to collagen, and crosslink formation

1 Department of Paediatrics and Adolescent Medicine, Johannes Kepler University Linz, Linz, Austria
2 Shriners Hospital for Children-Canada, Montreal, QC, Canada
3 Department of Human Genetics, McGill University, Montreal, QC, Canada
4 Department of Medical Engineering and Applied Social Sciences, University of Applied Sciences Upper Austria, Linz, Austria
5 Centre for Anatomy and Cell Biology, Medical University of Vienna, Vienna, Austria
6 Center for Medical Genetics, Department of Biomolecular Medicine, Ghent University Hospital, Ghent University, Ghent, Belgium
7 Clinical Department of Laboratory Medicine Proteomics Core Facility, Medical University Vienna, Vienna, Austria
8 Institute of Pathophysiology, Medical University of Innsbruck, Innsbruck, Austria
9 University Children's Hospital, Salzburger Landeskliniken (SALK) and Paracelsus Medical University (PMU), Salzburg, Austria
10 Department of Dermatology, Johannes Kepler University Linz, Austria
11 Biomedical Mass Spectrometry, Center for Medical Research, Johannes Kepler University, Linz, Austria
12 Department of Endocrinology and Diabetes, Birmingham Women's and Children's Hospital NHS Foundation Trust, Birmingham, UK
13 Institute of Applied Health Research, University of Birmingham, Birmingham, UK
14 The Institute of Metabolism and Systems Research, University of Birmingham, Birmingham, UK
15 1st Medical Department Hanusch Hospital, Ludwig Boltzmann Institute of Osteology at Hanusch Hospital of OEGK and AUVA Trauma Centre Meidling, Vienna, Austria
16 Vienna Bone and Growth Center, Vienna, Austria
*Corresponding author. Tel: +43 (0)5 7680 84 22001; Fax: +43 (0)5 7680 84 22004; E-mail: wolfgang.hoegler@kepleruniklinikum.at
†Present address: Department of Hematology and Internal Oncology, Johannes Kepler University, Linz, Austria
‡Present address: Bruker Austria, Vienna, Austria

© 2023 The Authors. Published under the terms of the CC BY 4.0 license.

(Canty & Kadler, 2005; Forlino *et al*, 2011; Byers & Pyott, 2012; Marini *et al*, 2014; Claeys *et al*, 2021; Jovanovic *et al*, 2022). In a small proportion, the causative genetic defects are yet to be identified.

SEC16 was first identified in the yeast *S. cerevisiae* as a peripheral membrane protein that affects coat protein complex II (COPII) assembly and vesicle budding from the endoplasmic reticulum (ER) (Espenshade *et al*, 1995; Gimeno *et al*, 1996; Shaywitz *et al*, 1997; Supek *et al*, 2002). In vertebrates, the SEC16 family consists of two genes, *SEC16A* and *SEC16B* that encode SEC16A (250 kDa) and SEC16B (117 kDa), respectively (Watson *et al*, 2006; Bhattacharyya & Glick, 2007; Iinuma *et al*, 2007). These play key roles in biogenesis and maintenance of ER exit sites (ERES), which are ribosome-free regions of the rough ER where COPII carriers are formed to ferry large proteins toward distal compartments (Hughes *et al*, 2009; Aridor, 2018). While the role of SEC16A in ER export is well established, less is known about SEC16B (Budnik & Stephens, 2009). SEC16B can bind to SEC16A, localizes to ERES, and plays a role in peroxisome biogenesis (Bhattacharyya & Glick, 2007; Yonekawa *et al*, 2011).

To date, variants/polymorphisms in *SEC16B* have been linked to obesity and type 2 diabetes (Ng *et al*, 2010; Sahibdeen *et al*, 2018).

In the current study, we identified a homozygous variant in *SEC16B* exon 4 (NM_033127.3: c.424C>T; p.Arg142Trp) in a child with skeletal dysplasia resembling OI and characterized the bone ultrastructural phenotype in a transiliac bone sample. We show that in patient-derived fibroblast cells, procollagen trafficking was altered, and that cells accumulated COL1A1 in the ER. In addition, *SEC16B*-mutated patient-derived cells displayed ER stress, increased autophagy, and apoptosis. In line with an altered passage along the secretory pathway, patient cells secreted type I collagen with altered post-translational modifications. We finally show that the mutation changes the expression level of *SEC16B*, thereby explaining the biological phenotype. Apart from showing that SEC16B is a candidate gene in OI, we uncover a clear biological role in protein trafficking for this enigmatic protein.

Results

The clinical phenotype is characterized by leg bowing, vertebral fractures, low bone mass, musculoskeletal pain, short stature, and muscle weakness

This boy is one of the five offspring of a consanguineous couple originating from Yemen. Parents are first cousins. His siblings are reported to be well and there is no family history of skeletal dysplasia or other conditions. The patient had bowed lower limbs, which was first noted at age 2 years, and muscular hypotonia which resulted in delay of walking until age 3 years. At age 4.5 years, he was of normal weight, but short (93.5 cm, −2.1 SD, 0.4^{th} height percentile). Radiography demonstrated slightly abnormal growth plates around the knee joint and short, bowed femora and humeri (Fig 1A–C). At age 5 years, he underwent bilateral tibial osteotomies for genu varum deformity, and at age 7 years, he had bilateral femoral rodding surgery. He continued walking with a waddling gait, experienced chronic leg pain, and used a wheelchair at school. At the age of 9 years, the metaphyseal changes around the knee joint had improved but generalized osteopenia was noted. Vertebral fractures grade 1 (20–25% height reduction) in T8 and T10 and grade 2 (> 25% height reduction) in T6 and T7 were noted on lateral spine X-rays. Assessment of calcium–phosphate metabolism was repeatedly normal. Dual-energy X-ray absorptiometry (DXA) revealed a lumbar spine bone mineral density (LS BMD) *Z* score of −1.8 and a total body bone mineral density (TB BMD) *Z* score of −1.8.

At the age of 10 years, repeat DXA showed an LS bone mineral apparent density (BMAD) *Z* score of −2.8 and a total body less head (TBLH) BMD *Z* score of −1.8. At this point, intravenous pamidronate was commenced and later switched to zoledronate at 13 years of age and continued until age 17. Blue sclerae, dental abnormalities, and hearing loss were not observed as in classical forms of OI due to collagen gene mutations. A recessive form of OI was suspected, so a transiliac bone biopsy, blood samples, and skin fibroblasts were taken from the patient.

At the age of 18 years, his final height was 155.5 cm (−3.77 SD), weight 63.3 kg, BMI 26.17 kg/m^2, his vertebral fractures had remodeled, and bone mass had normalized (BMAD *z*-score -0.9, TBLH BMD *z*-score -1.1). He continued to have moderate muscle weakness and persistent pain in his knees and hips but had no backache or scoliosis.

The analysis of the transiliac biopsy sample showed reduced bone mass, low cortical and trabecular thickness, and increased heterogeneity of mineralization

Bone histomorphometry and quantitative backscattered electron imaging (qBEI) outcomes are compiled in Table 1. The iliac crest bone had very thin and irregularly shaped cortex, and small and mostly isolated trabecular features (Fig 2A and B). Bone histomorphometry demonstrated reduced trabecular bone volume (BV/TV: −45%), trabecular thickness (Tb.Th: −40%), and cortical width (Ct.Wi: −79%) compared to healthy controls (Table 1). This is a similar reduction as reported in children with mild OI, except for cortical thickness that was within the limits of values observed in children with severe forms of OI (Rauch *et al*, 2000). In contrast to classical OI, indices of bone formation (surface extent of osteoblasts and osteoid deposition) were not increased, whereas the osteoclast number was elevated (Fig 2C). Polarized light microscopy revealed the coexistence of ordered lamellae and very disordered matrix in trabecular bone (Fig 2D). The most striking histological abnormalities were found in cortical bone with areas of matrix that contained roundish cells that resembled chondrocytes and woven bone osteoblasts surrounded by poorly mineralized matrix (Fig 2E). The presence of cartilage-like matrix and primary woven bone can clearly be distinguished in Giemsa-stained sections (Fig 2F and G). Moreover, inclusions of mineralized cartilage were seen within some trabeculae (Fig 2H). Bone mineralization density distribution (BMDD) showed that the typical calcium content of the matrix (CaPeak) was elevated in both bone compartments, as seen in OI, whereas the average calcium content (CaMean) was in the upper normal range. This reflected an increase in the fraction of lowly mineralized (CaLow: trabecular bone +82% and cortical bone: +61%, vs. healthy reference) and highly mineralized matrix (CaHigh: trabecular bone 8-fold and cortical bone 72-fold, vs. healthy reference), resulting in high heterogeneity in mineralization (Table 1).

© 2023 The Authors

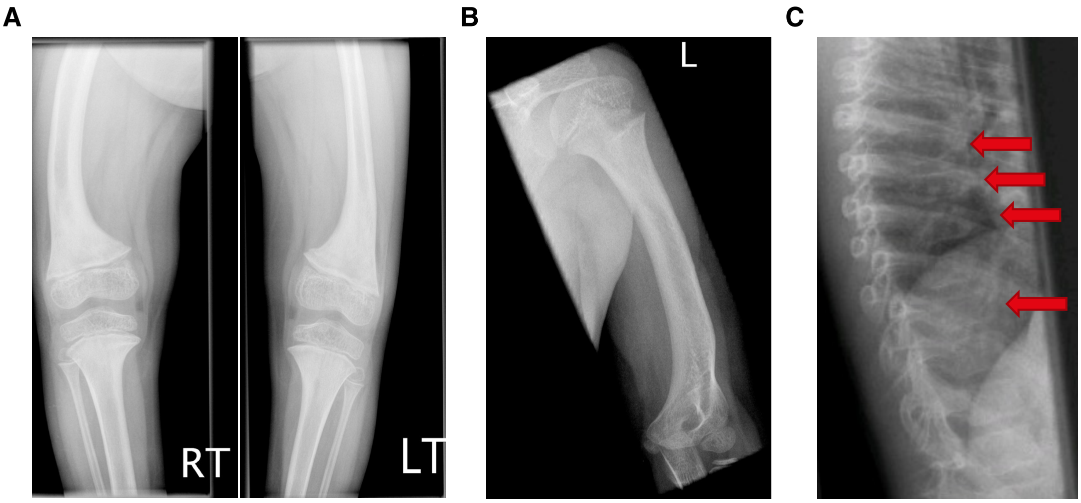

Figure 1. Radiographs demonstrate bowing deformity, short long bones, and vertebral fractures of the affected patient.

A–C (A) Bilateral bowing deformity and short femora and tibiae with atypical growth plates, (B) short and bowed humerus at age 4.5 years, and (C) vertebral fractures (arrows) on lateral imaging at age 9 years.

The backscattered electron microscopy image of the entire biopsy sample also demonstrated scarcity of mineralized bone and unusual protrusions of the cortex (Fig 2IA). The bone trabeculae had areas with hypomineralized matrix (< 18 wt % Ca, Fig $2Ia_1$) and very highly mineralized areas (> 30 wt % Ca, Fig $2Ia_2$) that corresponded mainly to inclusions of mineralized cartilage as seen in Giemsa-stained sections. The cortical compartment contained matrix as highly mineralized as cartilage and a substantial amount of matrix with graded mineralization between the values of CaPeak corresponding to mineralized bone and mineralized cartilage (Fig 2Ib and Ic) which resulted in an abnormal broadening of the bone mineralization density distribution (BMDD) curve (Fig 2J).

These abnormalities are also mirrored by the shape and position of the BMDD curves. The BMDD curves of cancellous and cortical bone are both shifted to the right, toward higher mineralization compared to healthy controls, with a very similar position of CaPeak (Fig 2J). In trabecular bone, a second small peak appears between 27 and 30 wt% Ca, corresponding to mineralized cartilage. In contrast, in cortical bone, there is a broad shoulder between the peak position and the mineralization densities corresponding to the presence of substantial amount of cartilaginous matrix.

Identification of a bi-allelic *SEC16B* variant

Whole-exome sequencing was performed and identified a homozygous missense variant in *SEC16B* exon 4 (NM_033127.3: c.424C>T; p.Arg142Trp, further referred to as SEC16B-R142W; Table EV1). Sanger sequencing confirmed homozygosity for the variant in the proband and determined that the asymptomatic mother and one brother were heterozygous for the variant (Fig 3A and B). Materials from the deceased father and three other siblings were not available to test.

This variant has a population heterozygote frequency in Africans/African Americans assessed in the United States of 1/470 individuals which predicts a homozygous frequency of about 1/910,000. This alteration is located in a region predicted to be involved in ER binding.

Fibroblasts with SEC16B-R142W exhibited altered type I procollagen trafficking from the ER

We analyzed the localization by immunofluorescence co-staining of type I collagen together with the ER marker KDEL. We found that patient fibroblasts exhibited higher intracellular pools of COL1A1 compared to control fibroblasts and that this intracellular pool co-localized with the ER marker KDEL (Fig 4A and B). We confirmed this finding by subcellular fractionation and found that COL1A1 and COL1A2 accumulated in the ER in patient cells in contrast to control cells (Fig 4C and D) and were less abundant in the Golgi fraction (Fig 4E and F). This is the first indication that collagen transport to the extracellular matrix is altered in cells with mutant SEC16B.

Next, we wanted to gain a deeper insight into possible mechanisms for impairment of ER-to-Golgi transport. Because SEC16B was proposed to exert its function at ERES (endoplasmic reticulum exit sites; Bhattacharyya & Glick, 2007; Budnik *et al*, 2011), we immunostained for SEC31A to assess whether control and patient cells have different numbers of ERES. Because of the impairment of ER export, we suspected that the number of ERES is downregulated in patient cells. To our surprise, the number of ERES was slightly, but consistently, higher in the patient fibroblasts (Fig 5A and B). While this does not explain the trafficking defect, the increase in ERES number is possibly the consequence of unfolded protein response (UPR) induction to compensate for the disruption of ER homeostasis by the ER export defect.

Analysis of secreted collagens from patient and healthy fibroblast medium by mass spectrometry revealed that three peptides derived from COL1A1 (start positions 269 GFSGLDGAK, 398 GAnGAPGIAG APGFPGAR, and 1171 TGDAGPVGPPGPPGPPGPPGPPsAGFDFSF LPQPPQEK) were detected only in the healthy control (Table EV2). This includes the phosphorylation site of serine 1193, which was

Table 1. Results of bone histomorphometry and qBEI analyses.

Parameters	Control values (7.0–10.9 years) (Glorieux *et al*, 2000)	Patient	OI type I (7.6 ± 3.8 years) (Rauch *et al*, 2000)
Histomorphometry			
BV/TV [%]	22.4 ± 4.2	12.40	11.0 ± 5.2
Md. BV/TV [%]	21.8 ± 4.0	11.94	*n.a.*
Tb. Th [μm]	129 ± 17	78.38	105.0 ± 25
Tb. N [/mm]	1.73 ± 0.17	1.58	1.3 ± 0.39
Ct. Wi [mm]	0.97 (0.37)	0.20	0.52 (0.20)
OV/BV [%]	2.64 (1.04)	3.71	5.2 (2.6)
O. Th [μm]	5.9 (1.1)	4.75	5.5 (1.7)
OS/BS [%]	29.1 (12.9)	32.04	48 (14)
Ob. S/BS [%]	8.2 (4.4)	7.78	19.4 ± 9.5
ES/BS [%]	17.0 (6.0)	9.52	15.6 [13.7–21.8]
Oc.S/BS [%]	1.29 (0.62)	2.29	1.37 [1.05–1.70]
N.Oc/BS [/mm]	0.36 (0.16)	0.79	0.47 (0.29)
qBEI: Bone mineralization density distribution (Roschger *et al*, 2008; Fratzl-Zelman *et al*, 2009)			
Trabecular bone			
CaMean [wt% Ca]	20.95 (0.57)	21.36	22.43 (0.63)
CaPeak [wt% Ca]	21.66 (0.52)	22.88	23.39 (0.57)
CaWidth [Δwt% Ca]	3.47 [3.12; 3.64]	3.64	3.08 (0.28)
CaLow [% bone area]	6.14 [4.90; 7.99]	11.22	5.94 (2.05)
CaHigh [% bone area]	0.89 [0.43; 1.47]	7.75	7.54 [5.00;11.82]
Cortical bone			
CaMean [wt% Ca]	20.45 [19.68; 21.04]	20.78	22.45 (0.57)
CaPeak [wt% Ca]	21.14 [20.62; 21.75]	22.53	23.25 (0.58)
CaWidth [Δwt% Ca]	3.81 [3.38; 4.38]	4.85	3.31 (0.31)
CaLow [% bone area]	9.06 [6.22; 15.00]	14.61	4.66 (0.86)
CaHigh [% bone area]	0.46 [0.28; 1.22]	12.69	8.08 (4.30)

Data are mean (± SD) or median [25th; 75th percentiles].

not detected in the patient when compared to the healthy control. Moreover, one peptide was found in the patient but not in the control (start position (K)QGPSGASGER(G)), this is located at residues 985–994. The absence of specific peptides in all replicates at high confidence suggests that flanking cleavage sites were resistant or inaccessible to digestion by trypsin.

To directly test the possibility of an ER export defect in patient fibroblasts, we conducted a RUSH (retention using selective hooks) assay (Boncompain *et al*, 2012). The RUSH assay allows the visualization of synchronous trafficking of a selected secretory protein from the ER to its target compartment. The secretory protein is trapped in the ER using streptavidin-based retention and treatment of cells with biotin relieves retention and allows the protein to exit the ER. Because we were interested in assessing ER-to-Golgi trafficking, we used a RUSH construct encoding for GFP-tagged mannosidase-II. Trafficking of mannosidase-II is COPII dependent and can therefore be used as a surrogate to measure ER export. Another advantage is that mannosidase-II exits the ER and traffics to the Golgi apparatus but not beyond. When we quantify the RUSH experiment, we measure the fluorescence in the Golgi region compared to the fluorescence outside the Golgi region. Thus, it is important that we are sure that the fluorescence outside the Golgi region originates only from the ER and not from any post-Golgi elements. We observed that patient cells were significantly slower in trafficking the reporter construct from ER to Golgi complex (Fig 5C and D). This result further supports the notion of a trafficking defect in cells with the SEC16B-R142W mutation.

Retardation of ER-to-Golgi trafficking often leads to proteostasis stress of the ER. In line with this was the finding of higher levels of autophagosome formation, in both electron micrographs (Fig 6A and B), by biochemically measuring LC3 lipidation status (Fig 6C and D). The increase in autophagy was most likely due to an enhanced level of ER stress in patient-derived cells. This notion was supported by the observation of higher levels of ER chaperones calnexin and BiP (Fig 4C), which might indicate that these cells experience ER stress. To test whether SEC16B mutant cells are more susceptible to ER stress, we treated them with tunicamycin and thapsigargin, two compounds that induce ER stress. As a surrogate for ER stress induction, we immunoblotted for XBP1s. We noticed that patient-derived fibroblasts exhibited an enhanced ER stress

 © 2023 The Authors

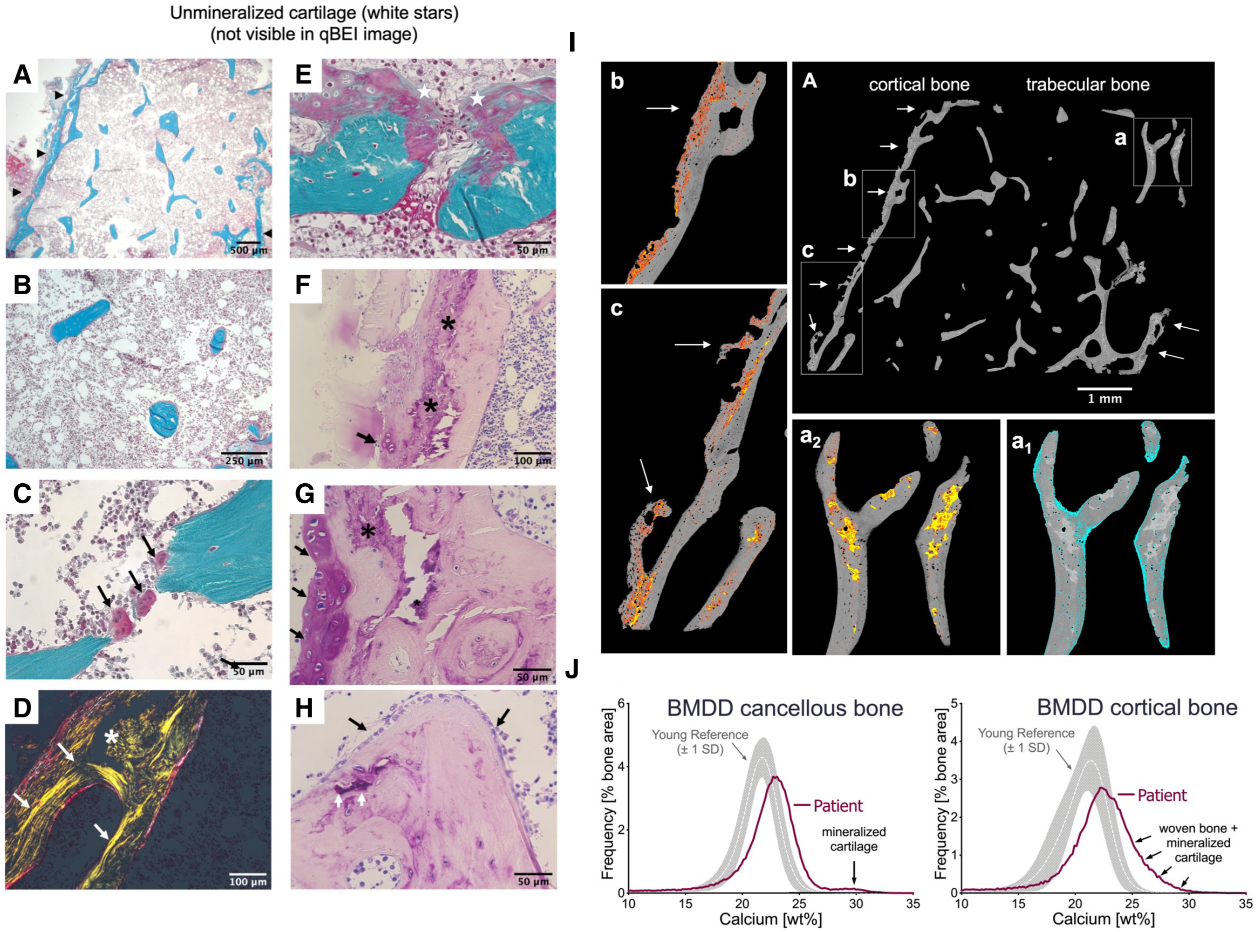

Figure 2. Bone Histology and quantitative backscattered electron imaging.

A–H Transiliac bone sample of the patient at age of 10 years viewed in the light microscope. (A–E) Goldner-stained sections, (F–H): Giemsa-stained sections. (A) Overview of the entire sample shows scarcity of mineralized bone (stained in green). Black arrowheads point toward the very thin cortices (right side, only a fractured cortex is visible). (B) Trabecular features are mostly small and isolated. (C) Black arrows point toward three bone-resorbing osteoclasts leading to the disconnection of two trabecular features. (D) Cortical bone viewed under polarized light: white arrows point toward collagen fibers arranged in a parallel concentric way around osteon next to a region where collagen fibers are randomly arranged, as typical for woven bone (white asterisk). (E) Detail from cortical bone showing roundish cells on the periosteal side surrounded by a mainly red, thus, poorly mineralized matrix (white stars). (F, G) Details from the cortex in Giemsa-stained section show areas with unmineralized cartilage (black arrows) and mineralized woven bone and/or mineralized cartilage (dark purple, black asterisks) and mineralized bone is stained pink. (H) Trabecular feature with mineralized cartilage inclusion stained deep purple (arrowhead), surrounded by bone tissue stained pink. Note many osteoblasts on the trabecular surface (black arrows)

I (A) Backscattered electron microscopy overview image of the entire sectional area shows very thin mineralized cortices (one intact, one fragmented, white arrows) and small and isolated trabecular features. The three rectangles (a, trabecular bone, b, c, cortical bone) are shown magnified. (a_1) trabecular features showing areas of lowly mineralized matrix: blue pixels represent matrix mineralized below 18 wt % Ca. Note that the colored pixels are localized mainly on trabecular surfaces and focally also in deeper regions of the trabeculae. (a_2) Same region as Aa_1 but here highly mineralized matrix is shown: yellow pixels represent matrix mineralized above 27.5 wt % Ca and red pixels represent matrix mineralized between 25 and 27.5 wt % Ca. Note the well-delineated inclusions which appear mostly yellow, corresponding according to the shape and the degree of mineralization to mineralized cartilage. (b, c) Details from the cortex: yellow pixels represent matrix mineralized above 27.5 wt % Ca, and red pixels represent matrix mineralized between 25 and 27.5 wt % Ca. Note also the protrusions of the cortex with areas of high porosity (white arrows in Ac).

J Bone mineralization density distribution (BMDD) of the patient trabecular and cortical bone compared to the corresponding reference of healthy children and adolescents—The patient's curves are shifted to the right toward higher calcium concentration compared to healthy references, indicating higher material density. Note that trabecular BMDD shows a second small peak at higher calcium concentration reflecting mineralized cartilage as viewed in yellow in Aa2. In contrast, the BMDD peak of cortical bone is broader and shows a "shoulder" at higher calcium concentrations indicating the presence of mineralized tissue with different material densities mirroring the red and yellow pixels in Ib and Ic. Gray bands represent reference BMDD ± 1 SD (Fratzl-Zelman *et al*, 2009).

Source data are available online for this figure.

response compared to control cells (Fig 6E). The proteostasis stress might also explain the higher level of cell death as indicated by enhanced cleavage of caspase 3 (Fig 6F and G). Taken together, these data show that the trafficking alteration has a negative impact on primary fibroblasts in the form of induction of ER stress, autophagy, and apoptosis.

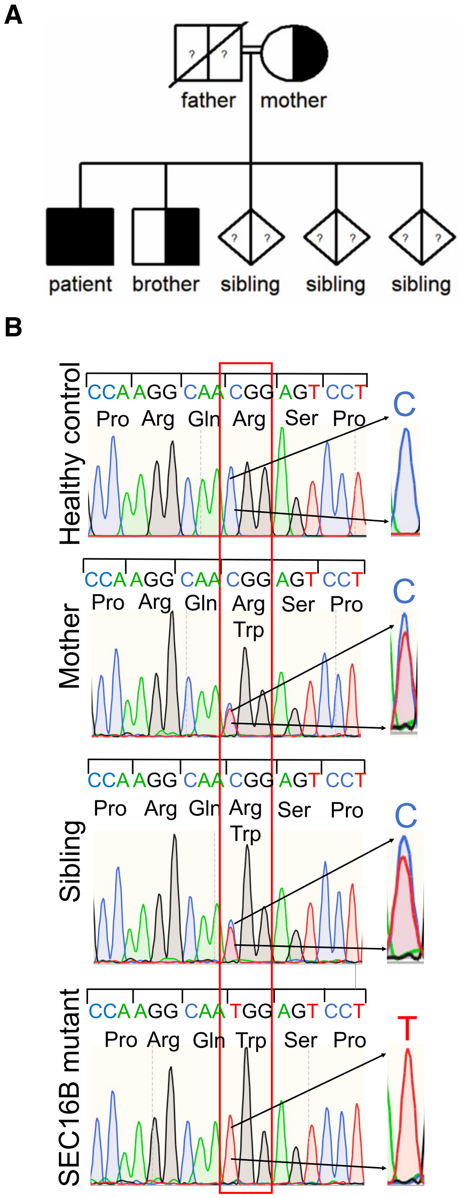

Figure 3. Homozygous missense mutation in *SEC16B*.

A Family pedigree with *SEC16B* homozygosity in the patient and heterozygosity in the mother and one brother.

B Validation of the *SEC16B* mutation by Sanger sequencing. Sanger sequencing of gDNA of the patient, brother, mother, and healthy control. Magnification of the electropherogram shows the position c.424 and the base change C→T.

Engineered patient cells expressing wt *SEC16B* do not accumulate COL1A1 in the ER

To verify that the *SEC16B* sequence variant underlies the ER export defect, we performed a rescue experiment. We stably expressed flag-tagged wild-type (wt) SEC16B in patient cells and used an empty vector as negative control (Fig 7A). Both sets of engineered patient cell lines (transfected with empty vector or wt *SEC16B*) were stained with antibody against KDEL (rER marker) and COL1A1. Expression of wt SEC16B in patient cells substantially reduced COL1A1 co-localization with the ER marker when compared to the empty vector-transfected cells (Fig 7B and C). In addition, the level of autophagy was also reduced by overexpression of flag-tagged SEC16B (Fig 7D and E), indicating that the proteostasis stress has been resolved. Taken together, these results confirm that the *SEC16B* variant disrupts COL1A1 trafficking from ER to Golgi complex in an *ex vivo* model and therefore, we next sought to mechanistically decipher how this mutation is linked to ER export.

Mutant SEC16B behaves as the wild-type protein

Replacement of a charged arginine by a bulky tryptophan might indicate an altered or a loss of function of mutant SEC16B. R142 is located immediately upstream of the central conserved domain that is thought to facilitate ERES localization. However, when we aligned human SEC16B with mouse SEC16B and human SEC16A, we noticed that R142 is not conserved with mouse SEC16B, while the upstream R140 was conserved in mouse SEC16B and human SEC16A. In addition, we noticed above that the presence of SEC16B-R142W does not affect the number of ERES significantly, which might be interpreted as such that this mutation does not affect the function of SEC16B. None of the commercially available antibodies to SEC16B could be used to localize the protein by immunofluorescence staining (Table EV3). To experimentally address whether the R142W mutation affects SEC16B function, we generated GFP-tagged versions of wild-type and mutant SEC16B and expressed them in HeLa cells, which are known to express very low levels of endogenous SEC16B. A hallmark of SEC16B function is its localization to ERES, which we assessed by co-localizing GFP-SEC16B with SEC13, a component of the outer COPII coat that stains all ERES. As shown in Fig 8A, wild-type and mutant SEC16B co-localized perfectly with SEC13, indicating that the R142W mutation does not interfere with subcellular localization of SEC16B. Next, we assessed the dynamics of SEC16B in living cells using fluorescence recovery after photobleaching (FRAP) microscopy where we bleach single ERES and monitor their fluorescence recovery. We have used this method before to assess the dynamics of SEC16A (Farhan *et al*, 2010; Tillmann *et al*, 2015). Using FRAP, we were unable to detect any substantial difference in the dynamics of mutant and wild-type SEC16B because both exhibited roughly the same mobile fractions and half-times of recovery (Fig EV1A–C).

Finally, we found that the interaction of mutant and wild-type SEC16B with SEC16A was not altered, which supports our notion that the mutation itself does not have a major effect on protein function (Fig EV1D).

Mutation of *SEC16B* reduces the expression levels, thereby explaining the biological phenotype

Since we did not detect a localization or turnover defect of mutant SEC16B, we compared the levels of SEC16B between patient and control fibroblasts. Because no reliable antibody for SEC16B exists, we used quantitative real-time PCR and observed a more than 70% reduction of SEC16B mRNA in patient fibroblasts (Fig 8B). We asked whether the reduction in SEC16B mRNA levels was due to different splicing and exon usage. We used primers covering the entire open

 © 2023 The Authors

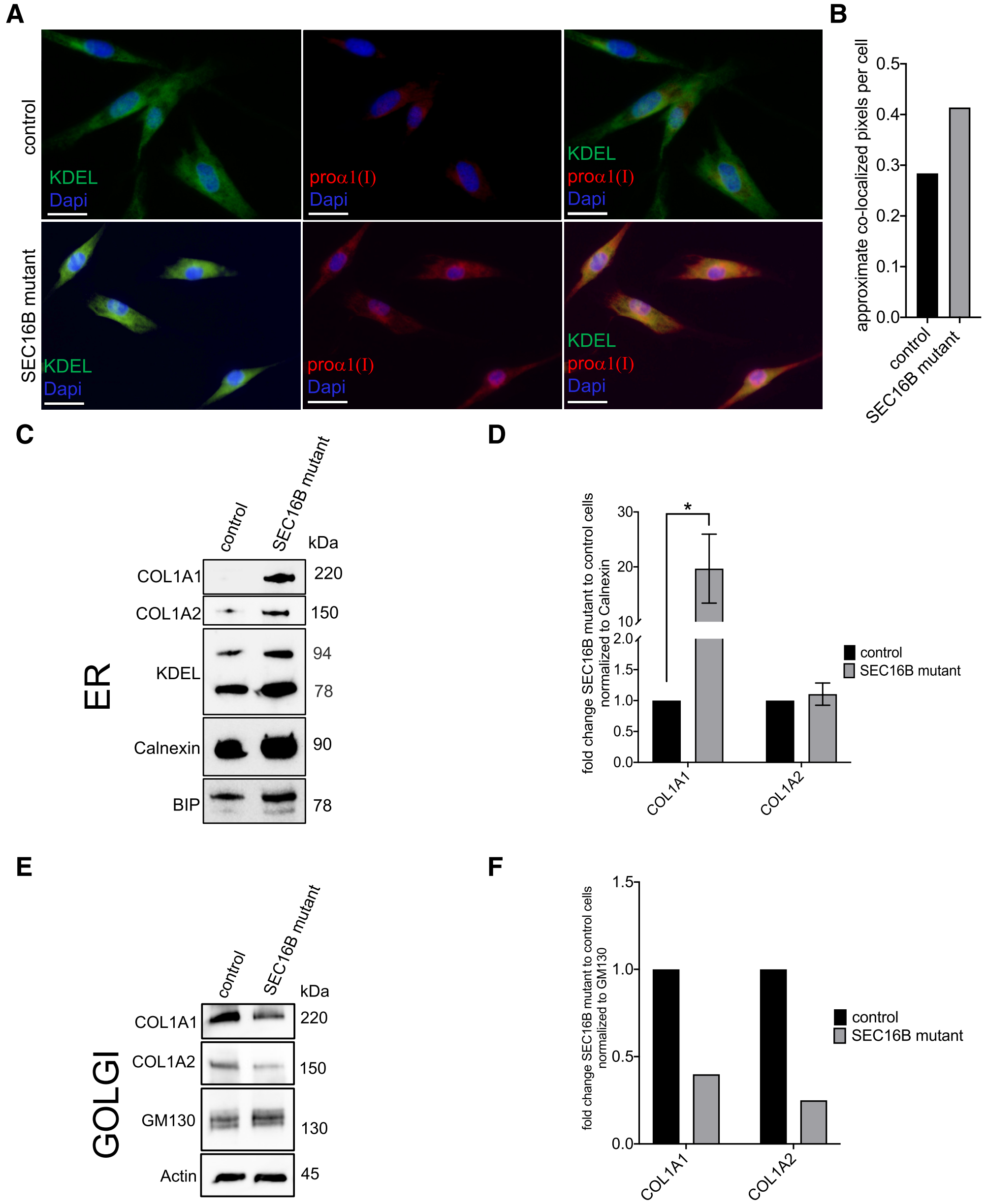

Figure 4. Expression of COL1A1 and COL1A2 in ER and Golgi complex by western blotting.

A Immunofluorescence labeling of the control and the patient fibroblasts for COL1A1 marker proα1 (I) and rER marker KDEL showed that COL1A1 is accumulated in ER in SEC16B mutant fibroblast when compared to control cells. Scale bar is 25 μm.

B Cellular analysis of the images was performed by first identifying cells based on nuclear recognition (DAPI stain), then measuring fluorescence intensity of the estimated cytoplasmic areas of each cell. A mean intensity threshold above background was used to determine positivity for each fluorochrome within the cytoplasm, thereby defining number of pixels as either positive or negative for each marker. The positive pixel data were then used to define co-localized areas. The approximate co-localized pixels per cell were calculated using data from multiple algorithms.

C–F (C) COL1A1 and COL1A2 expression in the ER of SEC16B patient and control fibroblasts assessed by western blotting. KDEL, calnexin, BIP, and PDI, all ER-resident proteins, were used as ER markers. (E) Golgi complex was isolated according to the method described in Current Protocols in Cell Biology, Unit 3.9, from healthy control cells and patient-derived fibroblasts. Subsequently, COL1A1 and COL1A2 expressions in the Golgi complex of SEC16B patient and control fibroblasts were determined by western blotting. GM130 (Golgi-resident protein) was used as marker for Golgi complex. All cells were treated with ascorbic acid 0.05 mg/ml for 20 h before the Golgi complex was isolated. (D, F) Quantification of the bands that was done with Bio-Rad Image Lab 6.1 software to calculate the expression levels. COL1A1 and COL1A2 band intensities were either normalized to (D) calnexin or (F) GM130. The normalized intensities were reported as fold change patients to control cells. Columns represent the mean of three (D) or two (F) independent experiments (raw values: 0.27 and 0.53 for COL1A1 and 0.29 and 0.21 for COL1A2), respectively. Biological replicates were performed; bars, standard error of the mean (SEM); *significant ($P < 0.05$) differences were obtained by using unpaired two-tailed *t*-test.

Source data are available online for this figure.

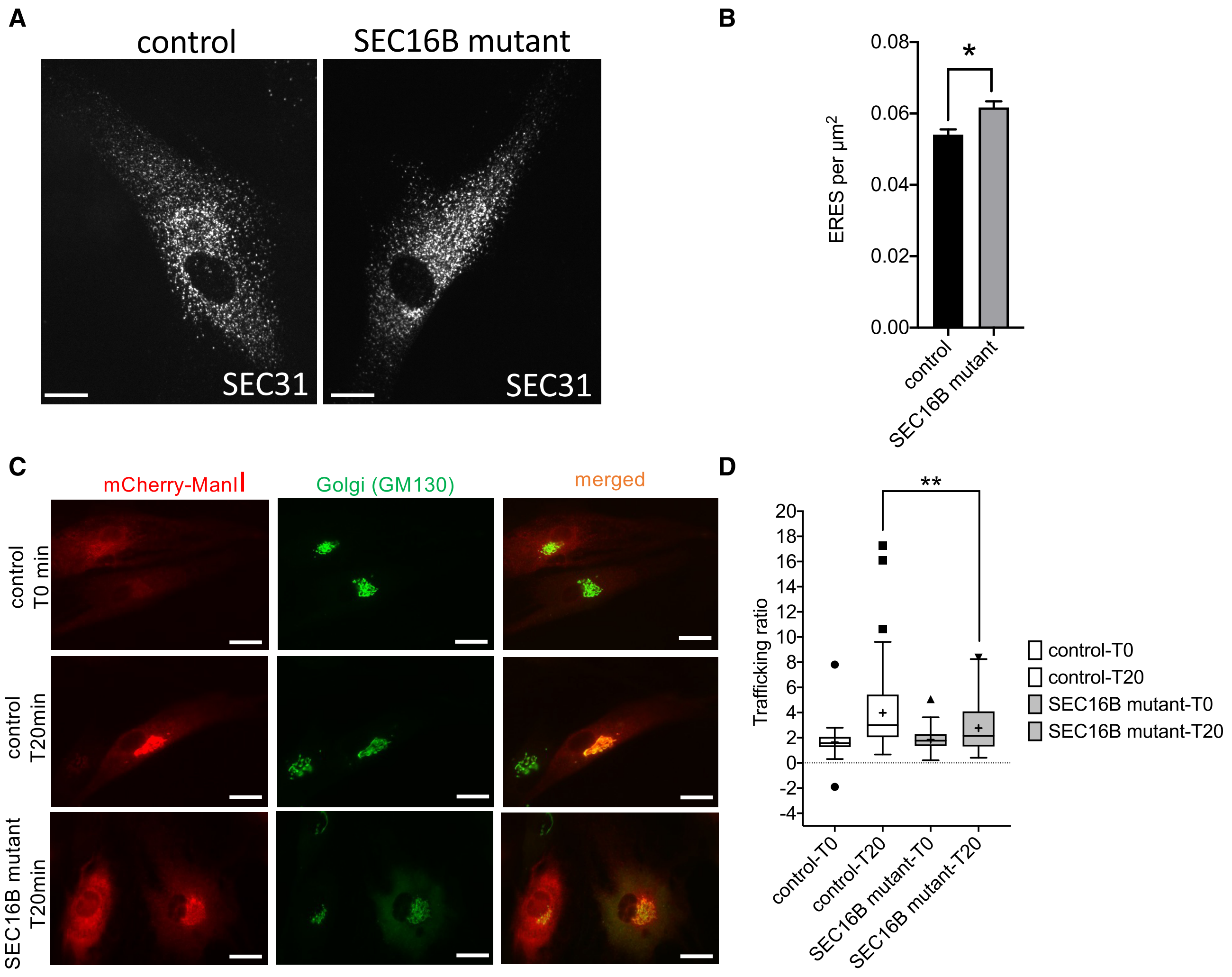

Figure 5. SEC16B mutant cells exhibit slower trafficking from ER to Golgi complex.

A Patient and control fibroblasts were plated on glass cover slips. After 24 h, cells were fixed and stained for SEC31 to label ERES. The scale bars are 10 µm.

B Quantification of the number of ERES per area from cells processed. The values are from four independent experiments and biological replicates. Seventy-one cells per experiment were acquired. Bars are means ± standard error of the mean (SEM); *significant ($P < 0.05$) differences obtained by using a paired two-tailed *t*-test.

C Patient and control fibroblasts were electroporated with plasmids encoding mCherry-tagged mannosidase II (ManII) as part of a RUSH construct. After 24 h, cells were fixed (T0) or treated with 40 mM biotin for 20 min (T20), followed by fixation and immunostaining for GM130 to label the Golgi complex. The scale bars are 10 µm.

D Quantification of 59 to 81 cells from three independent RUSH experiments (biological replicates). Biological replicates were performed. Central band represents the median. Whiskers are minimum and maximum values. Outliers are defined as > 1.5-fold of upper quartiles. ** Highly significant ($P < 0.005$) differences were obtained by unpaired two-tailed *t*-test.

Source data are available online for this figure.

reading frame of SEC16B from control and patient cells (Table EV4). To stabilize mRNAs, we also included a condition treated with cycloheximide (Fig EV2, Table EV4). We did not detect any appreciable alterations in the sequence, which indicates that there is no cryptic splicing site, intron retention, exon skipping, or deletion. Thus, the reason for the reduction in SEC16B mRNA is more complex and might be due to structural alteration of the mutant mRNA, differences in codon usage, or might be indirectly caused by an effect on secretion of factors controlling the levels of SEC16B mRNA. Examining all these possibilities experimentally is not a trivial task and is beyond the scope of the current work.

This reduction of SEC16B mRNA in patient fibroblasts is comparable to a knockdown and might explain the ER export defect. Previous attempts to test effects of SEC16B did not show any effect, but this is most likely due to the fact that these experiments were performed in HeLa cells, which express extremely low levels of SEC16B (Fig EV3). Therefore, we performed siRNA-mediated knockdown of SEC16B in HepG2 cells, which we validated to express SEC16B (Figs 8C and EV3). Effects on ER export were assessed using the RUSH assay using GFP with a signal sequence. This GFP variant enters the lumen of the ER and can be used as a generic marker for trafficking between the ER and the Golgi. Silencing SEC16B resulted

 © 2023 The Authors

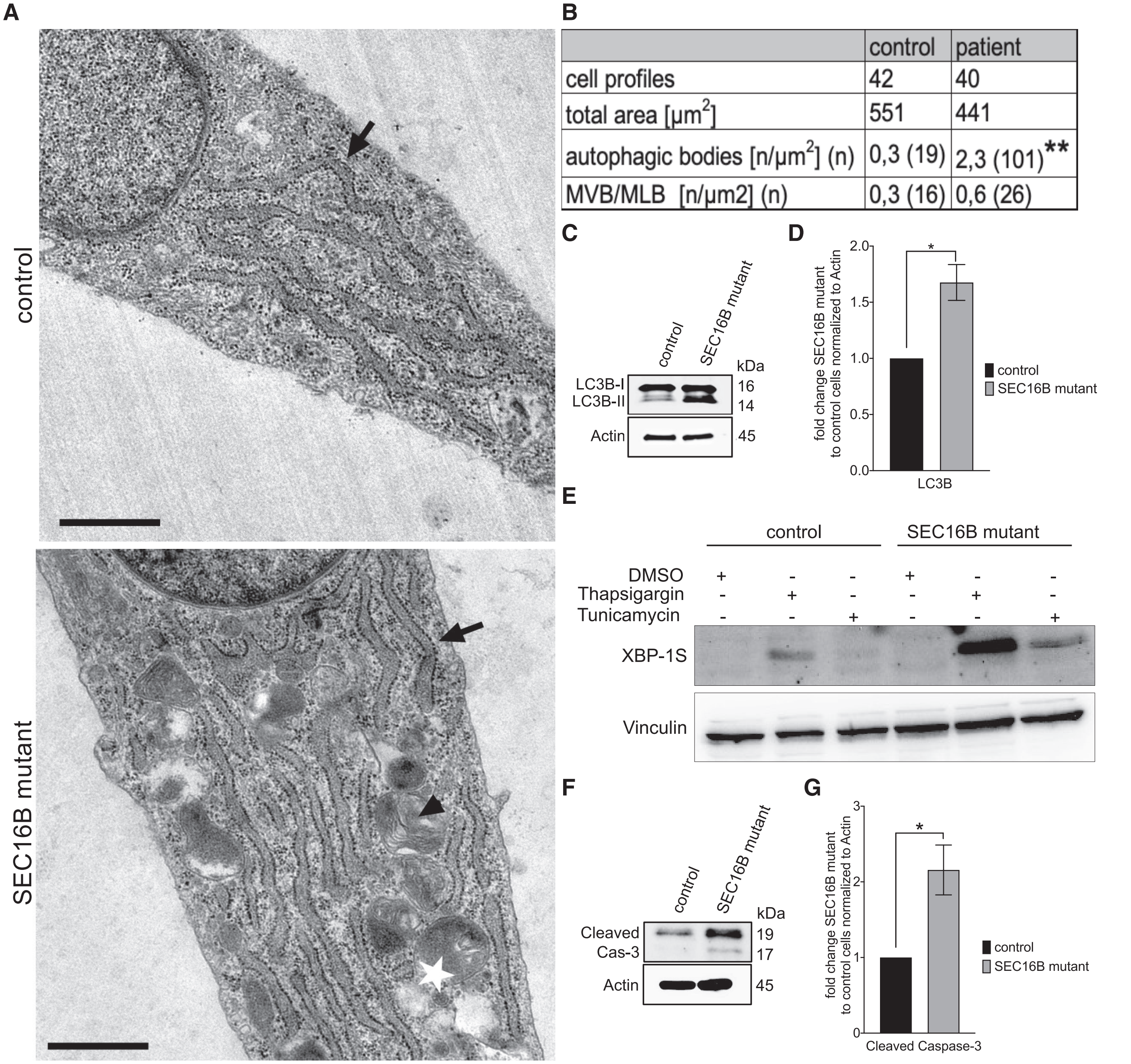

Figure 6. SEC16B mutant cells show enhanced autophagosome formation and promoted apoptosis.

A Micrograph of thin-section electron microscopy for fibroblasts from SEC16B mutant patient cells and healthy control cells. Arrows point to rER, arrowheads to MVBs/MLBs, and star indicates autophagosome. Scale bar is 1 μm. Summary of the number of autophagosomes.

B Autophagosomes were counted in 42 control and 40 SEC16B mutant patient cell micrographs with a total area of 551 and 441 μm^2, respectively. Nineteen autophagic bodies were counted in healthy control versus 101 in SEC16B mutant patient cells with the rate of 0.3 per μm^2 autophagic body in control to 2.3 per μm^2 in SEC16B mutant patient cells. **Highly significant ($P < 0.001$ differences were obtained by using a two-tailed *t*-test). MLB, multilamellar bodies; MVB, multivesicular body.

C Western blotting analysis of autophagy marker LC3B.

D Quantification of the bands. Columns represent the mean of three independent experiments, biological replicates were performed; bars, standard error of the mean (SEM); *significant ($P < 0.05$) differences obtained by using unpaired two-tailed *t*-test.

E Twenty-four hours after plating of patient and control fibroblasts, cells were treated with DMSO, 10 mM thapsigargin, or 1 mg/ml tunicamycin for 6 h. Cells were lysed and immunoblotted for XBP1s as a marker for ER stress and vinculin to ensure equal loading.

F Western blotting analysis of apoptosis marker cleaved caspase 3 shows enhanced expression in patient cells when compared to healthy control. One representative experiment out of three independent experiments is shown. Biological replicates were performed.

G Quantification of the bands. All band intensities were normalized to those of actin and reported as fold change patient to control cells. Columns represent the mean of three independent experiments, biological replicates were performed; bars, standard error of the mean (SEM); *significant ($P < 0.05$) differences obtained by using unpaired two-tailed *t*-test.

Source data are available online for this figure.

© 2023 The Authors

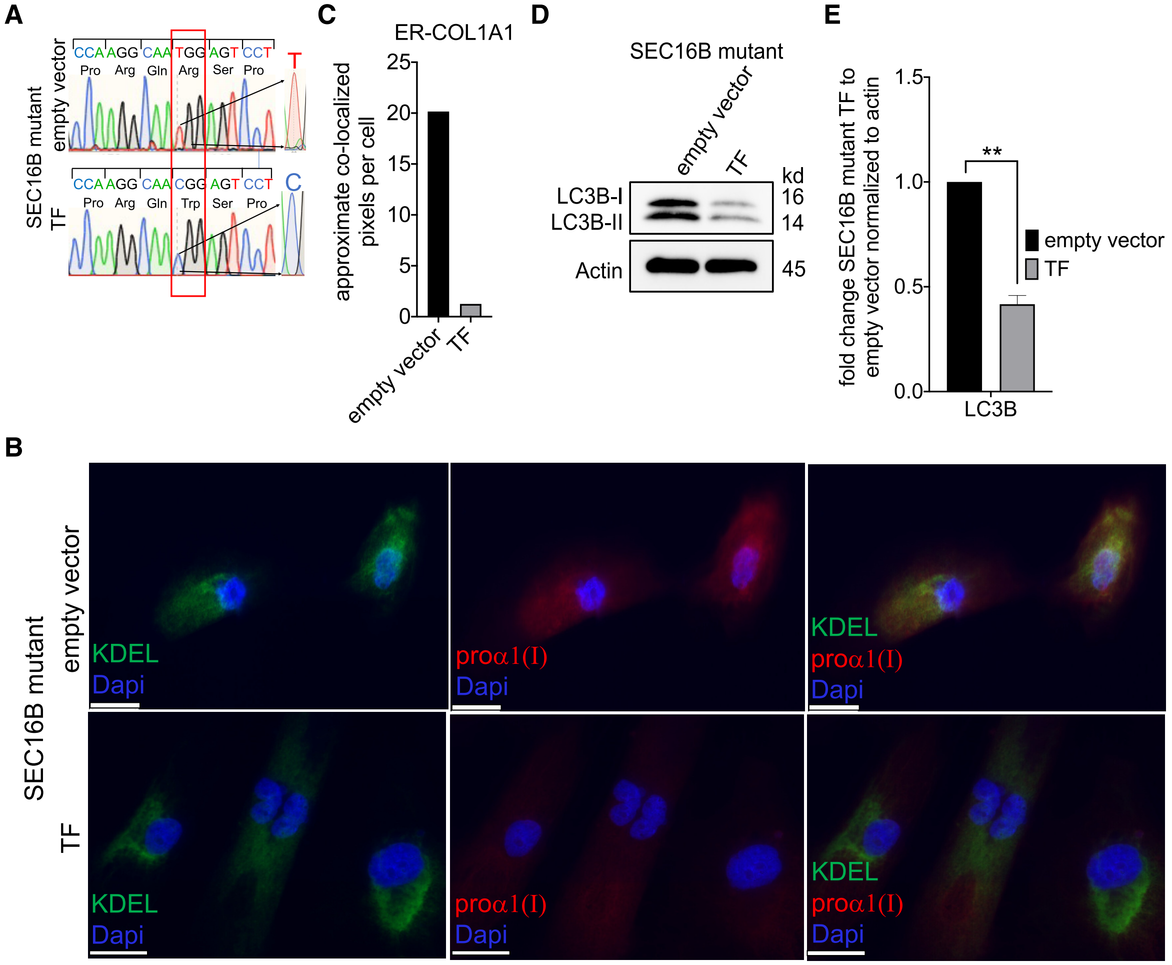

Figure 7. Patients cells transfected with *SEC16B* wt no longer retain COL1A1 in the ER.

Patient cells were either transfected with pCDNA3 empty vector (empty vector) or pcDNA3, which encodes wt SEC16B (TF). Cells were then selected with puromycin and single colonies were picked up and expanded. Established single colonies were used.

A Genomic DNA of patient cells either transfected with empty vector or with SEC16B wt plasmids was Sanger sequenced. Magnification of the electropherogram shows the position c.424 is corrected and T C as in the healthy control cells.

B Immunofluorescence labeling of the control and the patient fibroblasts for COL1A1 marker proα1 (I) and rough ER marker KDEL. The staining showed that while COL1A1 is accumulated in the ER of patient cells transfected with empty vector, it is not accumulated in the ER of the patient cells transfected with SEC16B wt. Scale bar is 25 μm.

C Quantification of approximate co-localized pixels per cell was performed as described above in Fig 4B.

D LC3B showed reduced expression in the TF SEC16B mutant cells (similar to expression in healthy control Fig 5B) when compared to empty vector-transfected patient cells.

E Quantification of the bands. All band intensities were normalized to those of actin and reported as fold change patient to control cells. Columns represent the mean of three independent experiments, biological replicates were performed; bars, standard error of the mean (SEM); **highly significant ($P < 0.001$ differences obtained by using unpaired two-tailed *t*-test).

Source data are available online for this figure.

in a decrease in the efficiency of ER-to-Golgi complex trafficking (Fig 8D and E), thus supporting the notion that the reduction in the levels of SEC16B in mutant fibroblasts underlies the observed trafficking defect.

Discussion

Research in the past five decades led to elucidation of the mechanisms and the machinery that controls secretory trafficking from the ER. However, the role of SEC16B remained elusive, and only a handful of papers aimed at investigating its biological effects without clear conclusions (Budnik *et al*, 2011). In our current work, we provide evidence that SEC16B regulates trafficking from the ER and that its loss of function results in osteogenesis imperfecta. The bone fragility is most likely caused by defects in collagen trafficking and consequently, an increased rate of cell death due to chronic ER stress. Our data are in agreement with previous reports in animal models and patients showing bone defects with mutations in genes involved in ER proteostasis. For instance, mutations in *SEC24D*, one

© 2023 The Authors

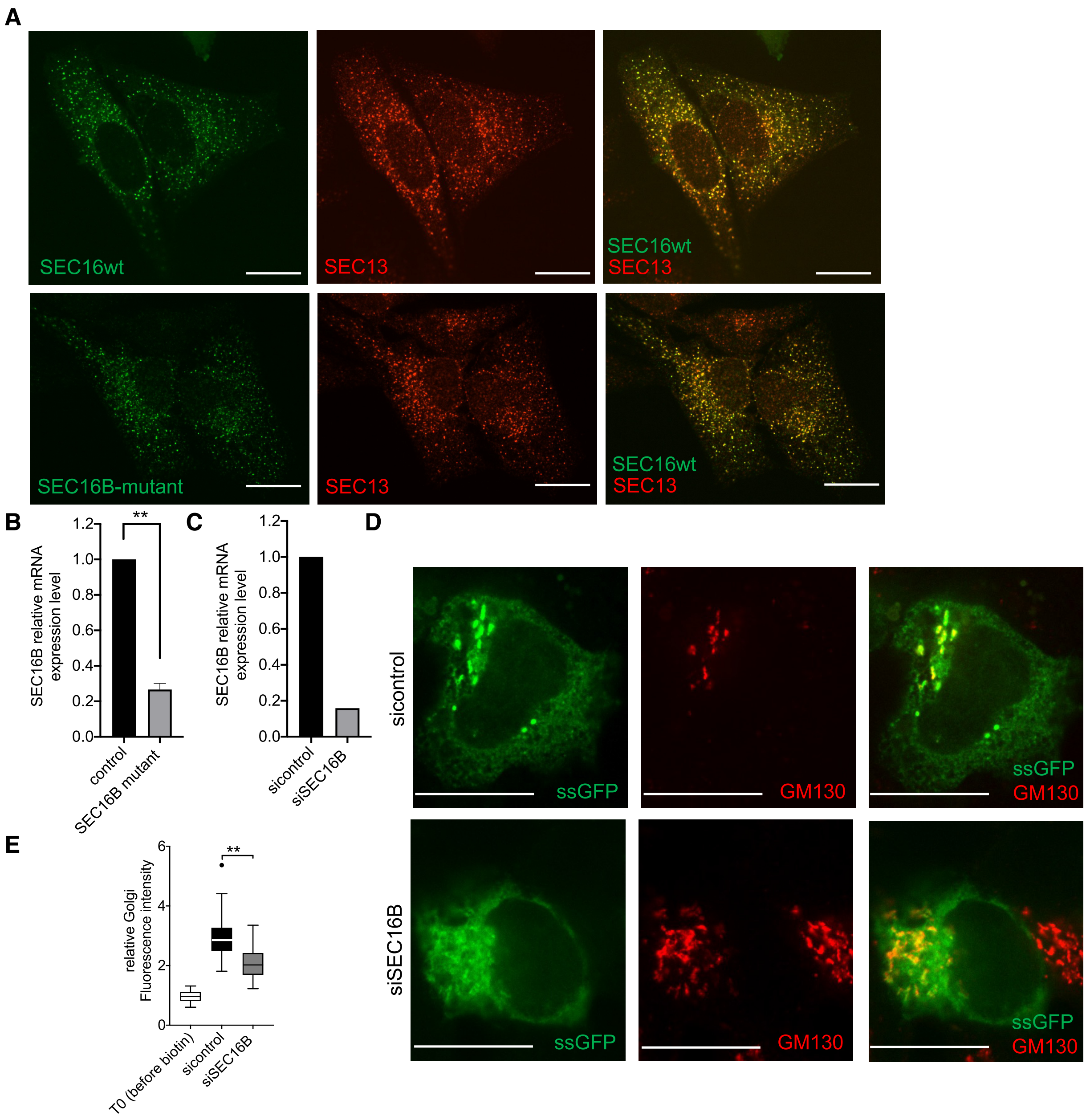

Figure 8. Knock-down SEC16B by siRNA resulted in a marked decrease in the efficiency of ER-to-Golgi complex trafficking.

A HeLa cells were transfected with plasmids encoding GFP-tagged SEC16B-R142W. After 24 h, cells were fixed and immunostained against SEC13 to label ERES. Green color shows SEC16B-R142W expression, red color shows SEC13 expression, and yellow color shows the overlap of the two colors. The scale bars are 10 μm.

B *SEC16B* mRNA expression in control and mutant cells analyzed by qRT–PCR. Columns represent the mean of $\Delta\Delta C_T$ values performed in triplicates and three independent experiments; biological replicates were performed. Actin was used as a reference gene to calculate ΔC_T values. Bars, standard error of the mean (SEM); **highly significant ($P < 0.01$) differences obtained by using unpaired two-tailed *t*-test.

C HepG2 cells were transfected with different SEC16B siRNAs. Quantification of the knockdown efficiency of *SEC16B* siRNA in HepG2 cells on mRNA level. Cells were lysed 72 h after siRNA transfection. Columns represent the mean of $\Delta\Delta C_T$ values performed in triplicates and two independent experiments; biological replicates were performed. Actin was used as a reference gene to calculate ΔC_T values.

D HepG2 cells were transfected with the indicated siRNA. After 48 h, cells were transfected with plasmids encoding signal sequence GFP (ssGFP) as part of a RUSH construct. After 24 h, cells were treated with 40 mM biotin for 15 min followed by fixation and immunostaining for GM130 to label the Golgi complex. The scale bars are 10 μm.

E Quantification of the RUSH assay using ssGFP in HepG2 cells. The central band represents the median. Whiskers are minimum and maximum values. Outliers are defined as > 1.5-fold of upper quartiles. The results are from four independent experiments (biological replicates) with 41–65 cells. The statistical test in Fig 8E is unpaired two tailed *t*-test.

Source data are available online for this figure.

of the COPII components, are known to cause retention of type I procollagen in the ER (Garbes *et al*, 2015). Similarly, mutations in *SEC23A* (Boyadjiev *et al*, 2006; Lang *et al*, 2006) and *SEC13* (Townley *et al*, 2008; Schmidt *et al*, 2013) lead to the accumulation of type I procollagen in the ER and consequently, to osteogenesis defects. TANGO1, a protein that regulates the biogenesis of ERES, was recently implicated in the transport of bulky collagens (Yuan *et al*, 2018). Of note, TANGO1 acts at the ERES in cooperation with SEC16A, a close homolog of SEC16B (Maeda *et al*, 2017). A truncated form of TANGO1 in humans has been implicated with dentinogenesis imperfecta and bone defects (Lekszas *et al*, 2020). Likewise, knockout of TANGO1 in mice also resulted in major bone defects (Guillemyn *et al*, 2021). Our current work expands this list by showing that SEC16B also regulates COL1A1 trafficking.

Through cell engineering, we confirmed the pathogenicity of the mutation by reversing the cellular disease phenotype. Overexpression of a SEC16B plasmid reversed the pathological phenotype with respect to subcellular collagen localization and autophagy induction.

Our analysis also revealed that the trafficking defect of collagen is accompanied by secretion of aberrant forms of collagen such as the absence of serine 1193 phosphorylation site in the patient. This phosphorylation site is located at a transition region from the triple helix to non-helix of COL1A1, and an amino acid substitution at this site is associated with OI (www.ncbi.nlm.nih.gov/clinvar/RCV000699710/). Furthermore, several COL1A1 peptides were either detectable only in the *SEC16B* wild-type or mutant background. Differences in detected peptides indicate that flanking cleavage sites were resistant or inaccessible to trypsin digestion. Post-translational modifications, such as acetylation or glycation, interfere with digestion by trypsin (Wang *et al*, 2002; Guan & Xiong, 2011; Rýglová *et al*, 2017). Likely, the absence of SEC16B results in impaired trafficking and processing of collagen. An explanation for this might come from the observation of metastability of proteins. After initial folding, some proteins are prone to misfold, especially under stress conditions. Probably, the longer residence time of proteins in the ER induces ER stress, thereby leading to processing defects by altering protein folding.

Besides suggesting SEC16B as a new candidate gene for OI, our work is the first to identify a wider biological role for this enigmatic protein. Most research so far focused on SEC16A, which has the closest homology to yeast Sec16, and consequently, little was known about SEC16B. Several reports found that polymorphisms in SEC16B are associated with obesity and metabolic disease. However, no functional analysis was performed, and it is therefore not clear what the impact of these polymorphisms is. Others have analyzed the consequences of depleting SEC16B in HeLa cells and found that it had no major effect on ERES function, thereby contributing to the enigmatic nature of this protein (Budnik *et al*, 2011; Yonekawa *et al*, 2011). However, it should be stressed that HeLa expresses 10- to 100-fold less SEC16B than SEC16A, thus providing a possible explanation for a lack of an effect of SEC16B knockdown. We show that silencing *SEC16B* negatively affects ERES function in cells with equal expression of SEC16B and SEC16A. Apparently, SEC16B is more critical in cell lines with a higher secretory burden, such as osteoblasts that mainly produce collagen type 1, which is bulky, and therefore, represents a bigger challenge to the ER-export machinery.

The observed highly mineralized cartilage within the trabeculae might be residual growth plate cartilage, which one would not expect in the ilium of a healthy 10-year-old child, but in our patient, this might be caused by the overall delay in skeletal growth and maturation. In contrast, the presence of unmineralized, mineralized cartilage and woven bone along the outer side of the cortex might possibly be newly formed from the periosteum that is well-known to have osteogenic and chondrogenic potential for reparative needs (Bisseret *et al*, 2015; Bahney *et al*, 2019; Jeyaraman *et al*, 2021). It is important to note that the cortical plates of our patient were about 200 μm thin, which in fact is close to the range of a trabecular plate and seemed fractured. As a consequence, microcracks and microfracture may have occurred triggering a repair mechanism with subsequent microcallus formation. Such a repair process would firstly require chondrogenic differentiation from periosteal cells and subsequent formation of woven bone. Such a woven bone was reported to have variable degrees of mineralization and large osteocyte lacunae, in line with our observations. Moreover, microcallus formation in the cortex can lead to piston-like distention, very similar to the cortical protrusions seen in the biopsy sample (Hahn *et al*, 1995; Okazaki *et al*, 2014). Of note, the patient had also multiple vertebral fractures as revealed by X-rays that would trigger repair mechanisms, including increased osteoclast recruitment (Bahney *et al*, 2019). Thus, the *SEC16B* mutation seems not only to impact endochondral bone formation but furthermore, results in low bone mass with microfractures, vertebral fractures, and altered bone material properties which together increase bone fragility.

In conclusion, we identified *SEC16B* as a new recessive OI gene. The homozygous mutation caused a distinct phenotype of bone deformity and fragility, reduced bone formation with thin cortices and trabeculae, distinct ultrastructural defects of the bone matrix, poor growth, pain, and muscle weakness. In patient fibroblasts, we demonstrate that the mutation results in lower SEC16B mRNA levels and thereby impairs the COPII machinery, reduces ER-to-Golgi complex transport of COL1A1, and causes ER stress with increased autophagy and apoptosis. In addition, our mass spectrometry analysis showed secretion of aberrant forms of COL1A1, including the absence of the serine 1193 phosphorylation site in the patient. Therefore, we assign another human phenotype to genetic defects in components of the ER export machinery.

Materials and Methods

Clinical information

This male patient first presented to the metabolic bone clinic at Birmingham Children's Hospital, Birmingham, UK, at the age of 2 years. Clinical details, radiographs, and densitometry information over the course of his routine clinical care were obtained from his medical records. Dual-energy X-ray absorptiometry was measured using a GE Lunar™ iDXA (GE Medical Systems, Madison, Wisconsin, USA). At age 9 years, a transiliac bone biopsy was performed to delineate the structural bone phenotype. At age 19 years, a skin biopsy was taken to enable functional testing of skin fibroblasts. Blood samples for DNA testing were collected from the patient, his mother, and an older brother (unable to obtain father's samples as deceased). Informed consent was obtained from the patient. Ethics approval has been obtained for whole-exome sequencing (Shriner's Hospital Montreal IRB A03-M28-11A) and cell culture work (Ethic

© 2023 The Authors

commission of the Medical Faculty JKU, Nr 1149/2020). These experiments conformed to the principles set out in the WMA Declaration of Helsinki and the Department of Health and Human Services Belmont Report.

Histomorphometry and backscattered electron microscopy

The transiliac bone biopsy was analyzed at the Ludwig Boltzmann Institute of Osteology (LBIO) in Vienna as described previously (El-Gazzar *et al*, 2021). Briefly, the bone sample was fixed in 70% ethanol, dehydrated by graded ethanol series, and embedded in polymethyl methacrylate. For light microscopy evaluation (histology and histomorphometry), 3-μm-thin sections were cut from the sample block and stained either by Goldner trichrome to distinguish mineralized bone (green) from unmineralized osteoid (red) or by Giemsa staining to distinguish mineralized bone (pink) from unmineralized cartilage (purple) and mineralized cartilage (dark purple; Gross & Strunz, 1977). The sections were then visualized in bright field and under polarized light with a light microscope equipped with a digital camera (Zeiss Jena, Germany, Axiophot with an Axiocam HRc). Histomorphometric outcomes were compared to reference values of healthy children and children with OI (Glorieux *et al*, 2000; Rauch *et al*, 2000). The remaining sample block was ground and polished to obtain a plane parallel surface which was subsequently carbon coated. qBEI was performed to evaluate bone mineralization density distribution (BMDD) using a Zeiss scanning microscope (DSM 962; Zeiss, Oberkochen, Germany). For the BMDD, the entire surface of the biopsy was scanned with a pixel resolution of 4 μm. Five different parameters are measured to characterize the obtained BMDD curve: (i) CaMean, the weighted mean calcium content; (ii) CaPeak, the most frequent calcium content; (iii) CaWidth, the width of the BMDD at half maximum; (iv) CaLow; and (v) CaHigh measuring the amount of lowly and highly mineralized bone (defined by the 5% and 95% percentiles of healthy adults). BMDD outcomes were compared to reference values of healthy children and children with OI (Roschger *et al*, 2008; Fratzl-Zelman *et al*, 2009).

Whole-exome sequencing

Whole-exome sequencing (library preparation, capturing, sequencing, and bioinformatics) was performed at the Genome Quebec Innovation Center in Montreal. The exome was captured by the SureSelect Human All Exon Kit version 4 (Agilent Technologies, Inc., Santa Clara, CA, USA). Subsequently, 100 bp paired-end reads were generated on an Illumina HiSeq 2000 sequencer. Alignment, variant calling, and annotation were performed using Burrows–Wheeler Aligner (BWA) (v.0.5.9) (Li & Durbin, 2009), SAMtools (Li *et al*, 2009) and Annovar (Wang *et al*, 2010), respectively. To find novel causative variant(s), we focused on those located in coding regions (i.e., nonsense, frameshift, and missense), splicing sites, and untranslated regions and having low allele frequency (< 0.005) in gnomAD and our in-house exome databases. Sanger sequencing (Applied Biosystems 3730xl sequencer; Applied Biosystems, Foster City, CA, USA) was used to confirm the presence of the homozygous *SEC16B* variant in the patient and to confirm the heterozygous state of the variant in his mother and brother.

Mass spectrometry

Patient and control dermal fibroblasts were treated with 0.05 mg/ml of ascorbic acid for 20 h in OptiMEM medium (Thermo Fisher scientific). The culture medium of the treated cells was collected and supplemented with 25× protease inhibitor cocktail (Sigma-Aldrich). Proteins were concentrated in an Ultra-15, 100K filter device (Sigma-Aldrich) by centrifuging at 5,000 *g* for 30 min. The protein samples were boiled at 100°C for 5 min, sonicated for 15 min, and cooled to ambient temperature. The solution was centrifuged for 5 min at 4°C and 10,000 rpm. Extracted proteins were digested as previously described (Fichtenbaum *et al*, 2016).

The nano-HPLC separation was performed using a nanoRSLC UltiMate 3000 HPLC system by Thermo Fisher. Mobile phases applied for sample loading, desalting, and separation were as follows: 2% AcN, 0.1% trifluoroacetic acid, and 0.01% aqueous heptafluorobutyric acid solution were used for sample loading applying a user-defined program for sample injection. The loading mobile phase was delivered to the trap column at 30 μl/min using the loading pump.

Trapping column used for sample loading, concentration, and clean-up was a 0.3 mm ID × 5 mm length C18 PepMap trap column (300A pore size and 3 μm particle size, Thermo Fisher scientific). Nano-chromatographic separation of peptides was performed on a C18 μPAC (μ-Pillar-Arrayed-Column, Pharma Fluidics) using flow rates of 800 and 600 nl/min. The gradient was formed as follows: An isocratic start with 5% B (50% AcN, 30% methanol [MeOH], 10% 2,2,2-trifluoroethanol [TFE], 10% water, and 0.1% formic acid [FA]) was maintained for 12 min at 800 nl/min and was followed by increasing the amount of B to 60% until 93 min at 600 nl/min. The column and the trap column were flushed with 90% B for 6 min, until 99 min, which was followed by column equilibration of 20 min at 800 nl/min.

Blank samples (injection of loading solvent) were run between sample injections for cleaning the separation system, preventing carryover, and background control.

Mass spectrometric detection and MS/MS analysis were performed using the Q-Exactive Plus Orbitrap BioPharma mass spectrometer (Thermo Fisher Scientific). Needle voltage was set to 2.8 kV in positive mode and the top 10 ions were selected for MS/MS analysis (fragmentation), resolution was set to 70,000 for full MS scans, ions with single charge were excluded from MS/MS analysis, and fragmented ions were excluded for 60 s from further fragmentation. Raw MS/MS files were analyzed using Proteome Discoverer 2.4 (Thermo Fisher Scientific) and searching the Swissprot human database and using following parameters:

- Taxonomy: Homo sapiens.
- Modifications: carbamidomethyl on C as fixed, carboxymethylation on M, as well as phosphorylation on S, T, and Y as variable modifications.
- Peptide tolerance was set to 10 ppm and the MS/MS tolerance to 0.05 Da.
- Trypsin was selected as the enzyme, and two missed cleavages were allowed.
- False discovery rate (FDR) was set to 1% and the decoy database search was used for estimating the FDR.

Final analysis was based on Scaffold (Proteome Software).

RNA isolation and qPCR

Total RNA from patient and control skin primary fibroblasts was isolated using Monarch® Total RNA Miniprep kit (New England Biolabs) according to the manufacturer's instructions. qPCR: the reaction was performed with Luna universal qPCR mix. The probe to detect SEC16B was from Qiagen: Hs_SEC16B_1_SG QuantiTect Primer Assay (GeneGlobe ID: QT00072751). In Fig 8B and C, the qPCR data are displayed as fold of control. The results were calculated as $\Delta\Delta C_T$ using actin as internal reference transcript. $\Delta\Delta C_T$ was calculated as ΔC_T of the SEC16B–ΔC_T of the control as in Fig 8B. To work out the fold gene expression, we performed $2^{-\Delta\Delta C_T}$.

PCR and targeted sanger sequencing

A 306 bp PCR product specific for the *SEC16B* mutation was amplified using the primer pair SEC16B-In-3F 5′-TAC TGT GCT CCA GGA GCT CCT CAC AGC-3′ and SEC16B-In-4R 5′-GCA GCA TCA GAC GCC AGA AGA TTT GCC-3′. The PCR was performed using Q5 Hot Start High-Fidelity 2xMaster Mix (New England BioLabs) with the following thermocycling conditions 30 s at 98°C followed by 33 cycles of 98°C for 10 s, 72°C for 30 s, and 72°C for 15 s. The purity and length of the PCR products were checked by agarose gel electrophoresis. An aliquot was treated with Exo-CIP Rapid PCR Cleanup (New England BioLabs) and sent to Eurofins for Sanger sequencing.

DNA polyacrylamide electrophoresis

SEC16B mutant cells were either left untreated or treated with cycloheximide at concentration of 100 μg/ml for 6 h. gDNA was isolated as indicated above. PCR with primers covering the entire open reading frame was performed (primer sequences are in Table EV4). PCR fragments were sequenced by sanger sequencing and separated on DNA polyacrylamide gel. Electrophoresis of 10% polyacrylamide gels was run in 0.5× TBE buffer (44.5 mM Tris base, 44.5 mM boric acid, and 1 mM EDTA, pH 8.0) at 100 V for 30 min.

Isolation of ER and Golgi complex

ER was isolated using Endoplasmic Reticulum isolation kit (#ER0100; Sigma) according to the manufacturer's instructions from healthy control and patient-derived fibroblasts. Briefly, cells were collected by conventional tissue culture methods. After washing the cell pellet with 1× PBS, it was resuspended in hypotonic extraction buffer and incubated for 20 min at 4°C. The cells were centrifuged at 600 *g* for 5 min and resuspended in isotonic extraction buffer. By using a Dounce homogenizer, the cells were broken and further centrifuged at 2,000 *g* for the Golgi complex isolation and at 1,000 *g* for the ER isolation for 10 min at 4°C. Then, the supernatant was centrifuged at 12,000 *g* for 15 min at 4°C to obtain the post-mitochondrial fraction (PMF). The PMF was further centrifuged at 100,000 *g* at 4°C to obtain the microsomal fraction in the pellet. The pellet was mixed with isotonic extraction buffer and homogenized by using a pestle. The microsomal sample was mixed with the 60% OptiPrep density gradient medium to a final concentration of 20% Optiprep. Then, a step gradient was prepared by pipetting first 30% Optiprep, then the sample containing 20% Optiprep, and finally, the 15% Optiprep solution into an ultracentrifuge tube. The gradient was centrifuged at 150,000 *g* for 3 h. Fractions of 500 μl were then withdrawn from the top of the gradient downward and further used for western blotting.

For the Golgi complex isolation, the supernatant was mixed with the 60% OptiPrep density gradient medium to a final concentration of 30% Optiprep. A step gradient of 0–25% Optiprep in isotonic extraction buffer was prepared. Then, the sample containing 30% Optiprep was pipetted beneath the step gradient and further centrifuged at 200,000 *g* for 3 h. Fractions of 500 μl were withdrawn from the top of the gradient downwards and further used for western blotting.

Protein preparation and western blot analysis

Patient and control dermal fibroblasts were treated with 0.05 mg/ml of ascorbic acid for 20 h in OptiMEM medium (Thermo Fisher scientific, # 15392402). For Fig 6D, cells were treated with DMSO (Sigma-Aldrich), 10 mM thapsigargin (Sigma-Aldrich), or 1 mg/ml tunicamycin (Sigma-Aldrich) for 6 h. Protein preparation and western blots were carried out using standard procedures. In short, cell lysates were prepared after washing the cells thrice with cold 1× PBS and lysed in RIPA buffer (Cell Signaling) supplemented with 100× protease inhibitor cocktail (Sigma-Aldrich) and 100× phosphatase inhibitor cocktail (Sigma-Aldrich) and 200× PMSF (Cell Signaling). Proteins were extracted from cell lysates by centrifugation at 14,000 *g* for 10 min at 4°C.

To analyze secreted proteins, culture medium samples were supplemented with 25× protease inhibitor cocktail (Sigma-Aldrich). Proteins were concentrated in an Ultra-15, 100K filter device (Sigma-Aldrich) by centrifuging at 5,000 *g* for 30 min. Protein concentrations were determined via the Thermo Scientific Pierce BCA protein assay kit (Thermo Fisher Scientific). All protein samples were denatured by adding 4× Laemmli sample buffer (Bio-Rad) with 2-mercaptoethanol and boiling them at 99°C for 5 min. Afterward, equal amounts of denatured proteins (~ 15 μg) were loaded onto 4–20% gradient gels (Bio-Rad), separated by gel electrophoresis, and transferred to PVDF membranes (Bio-Rad). The membranes were then incubated with EveryBlot blocking buffer (Bio-Rad) for 5 min. Membranes were probed with the following primary antibodies diluted in blocking buffer overnight: rabbit monoclonal anti-human COL1A1 (dilution 1:1,000, Cell Signaling, # 39952), rabbit polyclonal anti-human COL1A2 (dilution 1:1,000, Abcam, # ab96723), rabbit monoclonal anti-human actin (1:2,000, Cell Signaling, # 8457), rabbit monoclonal anti-human GM130 (1:1,000, Cell Signaling, # 12480), mouse monoclonal anti-human KDEL (1:1,000, Abcam, # ab12223), rabbit monoclonal anti-human calnexin (1:1,000, Cell Signaling, # 2679), rabbit monoclonal anti-human PDI (1:1,000, Cell Signaling, # 3501), rabbit monoclonal anti-human BiP (1:1,000, Cell Signaling, # 3177), mouse monoclonal anti- human vinculin (Sigma-Aldrich, # SAB4200080), mouse monoclonal anti-human XBP1 (BioLegend, # 647502), rabbit monoclonal anti-human cleaved caspase-3 (1:1,000, Cell Signaling, # 9664), polyclonal rabbit anti-human LC3B (1:1,000, Cell Signaling, # 2775), and polyclonal rabbit anti-human SEC16A (Bethyl Laboratories, # A300-648A). Membranes were then washed thrice with 1x TBS 0.1% Tween.

© 2023 The Authors

Detection was carried out using the corresponding peroxidase-conjugated (HRP) secondary antibodies (1:15,000, Cell Signaling) diluted in blocking buffer with incubation for 1 h at room temperature. The membranes were washed six times with 1× TBS 0.1% Tween and developed using Signal Fire Elite ECL Reagent (Cell Signaling).

Quantification of the bands was done with Bio-Rad Image Lab 6.1 software to calculate the expression levels. All band intensities were normalized to those of actin, GM130, or calnexin, respectively, and reported as fold change patient to control cells.

Immunoprecipitation

HeLa cells expressing GFP-tagged SEC16B were lysed in buffer (50 mM Tris–HCl, pH 7.4; 150 mM NaCl; 1% Triton X 100). The cleared lysate was subject to immunoprecipitation with 35 µl of anti-GFP nanobodies (generated in-house). After 18 h, beads were washed three times and proteins were eluted by addition of sample buffer.

Transmission electron microscopy

Cultured cells were fixed with 2.5% glutaraldehyde/2% PFA/0.1 M cacodylate at room temperature, scraped, and sedimented. After three rounds of washes with 0.1 M cacodylate buffer, pellets were post-fixed with 1% OsO_4/0.1 M cacodylate buffer. Dehydration in an ascending ethanol series and embedding in EPON resin was carried out following standard procedures. Ultrathin sections (60 nm) were contrasted with lead citrate and uranyl acetate and imaged with an FEI Tecnai20 electron microscope equipped with a 4K Eagle-CCD camera. Images were processed with Adobe Photoshop.

Immunofluorescence

5×10^4 cells were grown on chamber slides (Thermo Scientific) and then treated with ascorbic acid 0.05 µg/ml for 20 h. Subsequently, growth media were discarded and slides were washed twice in PBS. Cells were fixed with 10% neutral buffered formalin (10% NBF) for 15 min at room temperature. After that, cells were rinsed with 1× PBS (3×) for 5 min each and then air dried (overnight) and shipped to Fred Hutchinson Cancer Research Center (FHCRC), Seattle, Washington, USA. At FHCRC, cells were rehydrated, permeabilized, and incubated with primary antibody (KDEL Abcam # ab115638 at 5 µg/ml [1:200], or no antibody [protein block only]) under a coverslip and incubated overnight at 4–6°C.

The next day, Leica PowerVision anti-Mouse-HRP polymer (# PV6114) was applied, then the OPAL fluorophore (OPAL 540 [Akoya # FP1494A] at 1:50) was added. Slides were washed and incubated in stripping buffer (Biocare Denaturing Solution kit [# DNS001L] with a ratio of Denaturing Solution A to Denaturing Solution B 1:1) before being incubated in 3% hydrogen peroxide. Then, the second primary antibody rabbit polyclonal LF-39 Kerafast # ENH095-FP was used at 1:200 (antibody diluted in protein block), or no primary antibody (protein block only) was added. Then, Leica PowerVision anti-Rabbit-HRP (# PV6119) was applied and the OPAL fluorophore (OPAL 650 (Akoya # OP-001005) at 1:50) was added. The nuclei were counterstained in DAPI (5 µg/ml), then coverslips were applied using ProLong Gold mounting media (Invitrogen, # P36930).

For ERES immunofluorescence, cells were fixed with 3% paraformaldehyde (20 min at room temperature) followed by washing and permeabilization in 0.2% triton X 100 for 5 min at room temperature. Subsequently, cells were washed and incubated with the primary antibody for 1 h at room temperature. We used two antibodies to stain ERES: mouse monoclonal anti-Sec31 (BD Biosciences, # 612350, dilution of 1:1,000) and rabbit monoclonal anti-Sec13 (R&D Systems, # MAB9055, dilution 1:1,000).

Chamber slide analysis

Slides were cured overnight at room temperature then slide images were acquired on the Versa 200 (Leica BioSystems, Buffalo Grove, IL, USA) at 40×. The same size area was selected for each slide (1.4 cm^2). Image files were analyzed with HALO image analysis software (Indica Labs, Albuquerque, New Mexico, USA). Cellular analysis of the images was performed by first identifying cells based on nuclear recognition (DAPI stain), then measuring fluorescence intensity of the estimated cytoplasmic areas of each cell. Indica Labs HighPlex FL v3.1.0 module was used to obtain the total number of cells in the selected areas. A mean intensity threshold above background was used to determine positivity for each fluorochrome within the cytoplasm, thereby, defining number of pixels as either positive or negative for each marker. Indica Labs Area Quantification FL v2.1.2 module was used to obtain these co-localization data. The positive pixel data were then used to define co-localized areas. The approximate co-localized pixels per cell were calculated using data from these algorithms.

Retention using selective hooks (RUSH) experiment

The RUSH assay allows the visualization of synchronous trafficking of a selected secretory protein from the ER to its target compartment. The secretory protein is trapped in the ER using streptavidin-based retention and treatment of cells with biotin relieves retention and allows the protein to exit the ER. Fibroblasts were electroporated using a Neon electroporation system with 1 µg of plasmid (Ii-Str_ManII-SBP-EGFP) plated on glass coverslips (thickness 0.16 mm). In case of HepG2 cells, the transfection was carried out using Mirus Bio™ TransIT™-LT1 Transfection Reagent. After 24 h, cells were either fixed immediately with 3% paraformaldehyde (20 min at room temperature) or treated with biotin (40 µM) for 20 min followed by fixation. Cells were immunostained against GM130 (BD biosciences, #610822, dilution of 1:1,000) or anti-SEC31 (BD biosciences, # 612351, dilution of 1:1,000) to label the Golgi complex as described previously (Centonze *et al*, 2019). Trafficking efficiency was assessed by measuring the GFP fluorescence in the Golgi complex area normalized to the fluorescence outside the Golgi complex area. Imaging was performed on a Nikon Crest-V3 spinning disc confocal microscope using a 60x objective (CFI Apochromat TIRF 60× NA: 1.49). The microscope is equipped with a Prime BSI sCMOS camera (Teledyne Photometrics).

ERES staining and counting

Immunofluorescence of ERES was performed as described previously (Centonze *et al*, 2019). Anti-SEC13 (R&D Systems, # MAB9055-100) and anti-GFP (Sigma-Aldrich, # 11814460001) were used. Imaging was performed on a Nikon Crest-V3 spinning disc confocal microscope using a 60× objective (CFI Apochromat TIRF 60x NA: 1.49). ERES were counted using Fiji based on the 3D-LoG filtering method, where a LoG (Laplacian of Gaussian) is

© 2023 The Authors

The paper explained

Problem

Osteogenesis imperfecta (OI) is a clinically and genetically heterogeneous group of disorders characterized by low bone mass, recurrent fractures, and skeletal deformities. Type I collagen is produced by bone cells and secreted to the bone tissue (matrix), where it represents the core element for bone formation. In the current work, we report a patient with a homozygous mutation in SEC16B, and a bone formation defect reminiscent of osteogenesis imperfecta (OI). The role of the SEC16B protein in OI is currently unknown.

Results

This study demonstrates that the SEC16B mutation results in the reduction of mRNA expression rather than affecting protein function. Mechanistically, we demonstrate that reduced SEC16B expression results in defective secretory trafficking of collagen from the endoplasmic reticulum (ER) with increased ER stress and autophagy. These defects in the bone matrix lead to bone deformity and fragility.

Impact

In conclusion, the results of this study suggest SEC16B as a recessive candidate gene for OI, and uncover a clear biological role for this enigmatic protein. This new found role of the SEC16B protein on the overall cell process may lead to new areas of study for many disorders.

applied to a background-subtracted image followed by thresholding and counting as ERES any structure with a size of more than 4 pixel units (0.07 μm per pixel). For every image, the mask of counted ERES was superimposed on the raw ERES image to assess the faithfulness of our ERES counting approach.

Fluorescence recovery after photobleaching (FRAP) experiment

HeLa cells were transfected with plasmids encoding wild-type and mutant GFP-SEC16B. After 24 h, FRAP experiment on single ERES was performed on a Nikon CREST V3 spinning disc microscope. Each ERES was imaged one frame before bleaching and then for 12 frames with an interval of 2 s between frames. Bleaching was performed at approximately 30% laser intensity and for two frames using the zoom-in mode. For quantification, the pre-bleach value was set to 1 and all subsequent fluorescence intensities were normalized to the pre-bleach fluorescence.

Downregulation of SEC16B by siRNA

HepG2 cells were transfected with 5 nmol siRNA (SMARTPool On-TargetPlus from Dharmacon) using HiPerFect (Qiagen) according to the manufacturers' protocol. All readouts were measured 72 h after transfection. In case of a RUSH assay, the reporter plasmid was usually transfected 48 h after knockdown.

Statistics

To detect statistically significant differences between study group and controls, two-sided Student's *t*-tests were applied. *P*-values < 0.05 was considered for statistically significant differences. For statistical evaluation, GraphPad Prism 8 software was used. There was no randomization or blinding done for this study. Statistical analysis was performed for the experiments which were replicated at least three times. No samples were excluded from this study.

Data availability

This study includes no data deposited in external repositories. Due to the consent agreement restrictions, patient WES raw data could not be made freely available and are therefore not deposited in a public database.

Expanded View for this article is available online.

Acknowledgements
The authors thank Prof. Peter Byers (University of Washington School of Medicine) for the advice that he has given during the course of the study. Helena Hödlmayr (JKU), Teresa Burner, and Francesca Barbazza (JKU) for their technical assistance with immunoblotting, cell transfections, and cell culture work, and Sonja Lueger, Petra Keplinger, and Pheadra Messmer at the Ludwig Boltzmann Institute of Osteology in Vienna for technical assistance with sample preparation and analysis. We would also like to thank Tracy Goodpaster (Fred Hutchinson Cancer Research Centre) for her technical assistance with IF. This work was mainly funded by Prof Högler's institutional funds and, in part, by Prof Farhan's institutional funds, the AUVA (Research funds of the Austrian workers compensation board), and the OEGK (Austrian Social Health Insurance Fund), and a research prize awarded to Dr. Ahmed El-Gazzar by the Austrian Society for Bone and Mineral Research.

Author contributions
Ahmed El-Gazzar: Conceptualization; resources; data curation; formal analysis; supervision; validation; investigation; visualization; methodology; writing – original draft; project administration; writing – review and editing. **Barbara Voraberger:** Data curation. **Frank Rauch:** Data curation. **Mario Mairhofer:** Data curation. **Katy Schmidt:** Data curation. **Brecht Guillemyn:** Data curation; formal analysis. **Goran Mitulović:** Data curation. **Veronika Reiterer:** Data curation. **Margot Haun:** Data curation. **Michaela M Mayr:** Data curation. **Johannes A Mayr:** Formal analysis; investigation. **Susanne Kimeswenger:** Data curation. **Oliver Drews:** Formal analysis. **Vrinda Saraff:** Resources; data curation. **Nick Shaw:** Resources; project administration. **Nadja Fratzl-Zelman:** Data curation; formal analysis. **Sofie Symoens:** Data curation; formal analysis. **Hesso Farhan:** Formal analysis; investigation; writing – review and editing. **Wolfgang Högler:** Supervision; investigation; resources; writing – review and editing.

Disclosure and competing interests statement
The authors declare that they have no conflict of interest.

For more information
i https://www.kepleruniklinikum.at/kliniken-einrichtungen/kinder-und-jugendheilkunde/forschung/
ii https://omim.org/entry/612855?search=sec16b&highlight=sec16b
iii https://www.proteinatlas.org/ENSG00000120341-SEC16B
iv http://compbio.charite.de/phenomizer/
v https://www.ncbi.nlm.nih.gov/clinvar/
vi https://www.gtexportal.org/home/
vii https://version11.string-db.org/
viii https://www.pathwaycommons.org/
ix https://www.genecards.org/

 © 2023 The Authors

x https://www.uniprot.org/
xi https://oi.gene.le.ac.uk/
xii https://swissmodel.expasy.org/
xiii https://oife.org/
xiv https://www.care4brittlebones.org/en/

References

Aridor M (2018) COPII gets in shape: lessons derived from morphological aspects of early secretion. *Traffic* 19: 823–839

Bahney CS, Zondervan RL, Allison P, Theologis A, Ashley JW, Ahn J, Miclau T, Marcucio RS, Hankenson KD (2019) Cellular biology of fracture healing. *J Orthop Res* 37: 35–50

Bhattacharyya D, Glick BS (2007) Two mammalian Sec16 homologues have nonredundant functions in endoplasmic reticulum (ER) export and transitional ER organization. *Mol Biol Cell* 18: 839–849

Bisseret D, Kaci R, Lafage-Proust MH, Alison M, Parlier-Cuau C, Laredo JD, Bousson V (2015) Periosteum: characteristic imaging findings with emphasis on radiologic-pathologic comparisons. *Skeletal Radiol* 44: 321–338

Boncompain G, Divoux S, Gareil N, de Forges H, Lescure A, Latreche L, Mercanti V, Jollivet F, Raposo G, Perez F (2012) Synchronization of secretory protein traffic in populations of cells. *Nat Methods* 9: 493–498

Boyadjiev SA, Fromme JC, Ben J, Chong SS, Nauta C, Hur DJ, Zhang G, Hamamoto S, Schekman R, Ravazzola M *et al* (2006) Cranio-lenticulo-sutural dysplasia is caused by a SEC23A mutation leading to abnormal endoplasmic-reticulum-to-Golgi trafficking. *Nat Genet* 38: 1192–1197

Budnik A, Stephens DJ (2009) ER exit sites – localization and control of COPII vesicle formation. *FEBS Lett* 583: 3796–3803

Budnik A, Heesom KJ, Stephens DJ (2011) Characterization of human Sec16B: indications of specialized, non-redundant functions. *Sci Rep* 1: 77

Byers PH, Pyott SM (2012) Recessively inherited forms of osteogenesis imperfecta. *Annu Rev Genet* 46: 475–497

Canty EG, Kadler KE (2005) Procollagen trafficking, processing and fibrillogenesis. *J Cell Sci* 118: 1341–1353

Centonze FG, Reiterer V, Nalbach K, Saito K, Pawlowski K, Behrends C, Farhan H (2019) LTK is an ER-resident receptor tyrosine kinase that regulates secretion. *J Cell Biol* 218: 2470–2480

Claeys L, Storoni S, Eekhoff M, Elting M, Wisse L, Pals G, Bravenboer N, Maugeri A, Micha D (2021) Collagen transport and related pathways in osteogenesis imperfecta. *Hum Genet* 140: 1121–1141

El-Gazzar A, Hogler W (2021) Mechanisms of bone fragility: from osteogenesis imperfecta to secondary osteoporosis. *Int J Mol Sci* 22: 625

El-Gazzar A, Mayr JA, Voraberger B, Brugger K, Blouin S, Tischlinger K, Duba HC, Prokisch H, Fratzl-Zelman N, Hogler W (2021) A novel cryptic splice site mutation in COL1A2 as a cause of osteogenesis imperfecta. *Bone Rep* 15: 101110

Espenshade P, Gimeno RE, Holzmacher E, Teung P, Kaiser CA (1995) Yeast SEC16 gene encodes a multidomain vesicle coat protein that interacts with Sec23p. *J Cell Biol* 131: 311–324

Farhan H, Wendeler MW, Mitrovic S, Fava E, Silberberg Y, Sharan R, Zerial M, Hauri HP (2010) MAPK signaling to the early secretory pathway revealed by kinase/phosphatase functional screening. *J Cell Biol* 189: 997–1011

Fichtenbaum A, Schmid R, Mitulovic G (2016) Direct injection of HILIC fractions on the reversed-phase trap column improves protein identification rates for salivary proteins. *Electrophoresis* 37: 2922–2929

Forlino A, Cabral WA, Barnes AM, Marini JC (2011) New perspectives on osteogenesis imperfecta. *Nat Rev Endocrinol* 7: 540–557

Fratzl-Zelman N, Roschger P, Misof BM, Pfeffer S, Glorieux FH, Klaushofer K, Rauch F (2009) Normative data on mineralization density distribution in iliac bone biopsies of children, adolescents and young adults. *Bone* 44: 1043–1048

Garbes L, Kim K, Riess A, Hoyer-Kuhn H, Beleggia F, Bevot A, Kim MJ, Huh YH, Kweon HS, Savarirayan R *et al* (2015) Mutations in SEC24D, encoding a component of the COPII machinery, cause a syndromic form of osteogenesis imperfecta. *Am J Hum Genet* 96: 432–439

Gimeno RE, Espenshade P, Kaiser CA (1996) COPII coat subunit interactions: Sec24p and Sec23p bind to adjacent regions of Sec16p. *Mol Biol Cell* 7: 1815–1823

Glorieux FH, Travers R, Taylor A, Bowen JR, Rauch F, Norman M, Parfitt AM (2000) Normative data for iliac bone histomorphometry in growing children. *Bone* 26: 103–109

Gross UM, Strunz V (1977) Surface staining of sawed sections of undecalcified bone containing alloplastic implants. *Stain Technol* 52: 217–219

Guan KL, Xiong Y (2011) Regulation of intermediary metabolism by protein acetylation. *Trends Biochem Sci* 36: 108–116

Guillemyn B, Nampoothiri S, Syx D, Malfait F, Symoens S (2021) Loss of TANGO1 leads to absence of bone mineralization. *JBMR Plus* 5: e10451

Hahn M, Vogel M, Amling M, Ritzel H, Delling G (1995) Microcallus formations of the cancellous bone: a quantitative analysis of the human spine. *J Bone Miner Res* 10: 1410–1416

Hughes H, Budnik A, Schmidt K, Palmer KJ, Mantell J, Noakes C, Johnson A, Carter DA, Verkade P, Watson P *et al* (2009) Organisation of human ER-exit sites: requirements for the localisation of Sec16 to transitional ER. *J Cell Sci* 122: 2924–2934

Iinuma T, Shiga A, Nakamoto K, O'Brien MB, Aridor M, Arimitsu N, Tagaya M, Tani K (2007) Mammalian Sec16/p250 plays a role in membrane traffic from the endoplasmic reticulum. *J Biol Chem* 282: 17632–17639

Jeyaraman M, Muthu S, Gangadaran P, Ranjan R, Jeyaraman N, Prajwal GS, Mishra PC, Rajendran RL, Ahn BC (2021) Osteogenic and chondrogenic potential of periosteum-derived mesenchymal stromal cells: do they hold the key to the future? *Pharmaceuticals (Basel)* 14: 1133

Jovanovic M, Guterman-Ram G, Marini JC (2022) Osteogenesis imperfecta: mechanisms and signaling pathways connecting classical and rare OI types. *Endocr Rev* 43: 61–90

Lang MR, Lapierre LA, Frotscher M, Goldenring JR, Knapik EW (2006) Secretory COPII coat component Sec23a is essential for craniofacial chondrocyte maturation. *Nat Genet* 38: 1198–1203

Lekszas C, Foresti O, Raote I, Liedtke D, Konig EM, Nanda I, Vona B, De Coster P, Cauwels R, Malhotra V *et al* (2020) Biallelic TANGO1 mutations cause a novel syndromal disease due to hampered cellular collagen secretion. *Elife* 9: e51319

Li H, Durbin R (2009) Fast and accurate short read alignment with burrows-wheeler transform. *Bioinformatics* 25: 1754–1760

Li H, Handsaker B, Wysoker A, Fennell T, Ruan J, Homer N, Marth G, Abecasis G, Durbin R, Genome Project Data Processing S (2009) The sequence alignment/map format and SAMtools. *Bioinformatics* 25: 2078–2079

Maeda M, Katada T, Saito K (2017) TANGO1 recruits Sec16 to coordinately organize ER exit sites for efficient secretion. *J Cell Biol* 216: 1731–1743

Marini JC, Reich A, Smith SM (2014) Osteogenesis imperfecta due to mutations in non-collagenous genes: lessons in the biology of bone formation. *Curr Opin Pediatr* 26: 500–507

Ng MC, Tam CH, So WY, Ho JS, Chan AW, Lee HM, Wang Y, Lam VK, Chan JC, Ma RC (2010) Implication of genetic variants near NEGR1, SEC16B,

TMEM18, ETV5/DGKG, GNPDA2, LIN7C/BDNF, MTCH2, BCDIN3D/FAIM2, SH2B1, FTO, MC4R, and KCTD15 with obesity and type 2 diabetes in 7705 Chinese. *J Clin Endocrinol Metab* 95: 2418–2425

Okazaki N, Chiba K, Taguchi K, Nango N, Kubota S, Ito M, Osaki M (2014) Trabecular microfractures in the femoral head with osteoporosis: analysis of microcallus formations by synchrotron radiation micro CT. *Bone* 64: 82–87

Rauch F, Travers R, Parfitt AM, Glorieux FH (2000) Static and dynamic bone histomorphometry in children with osteogenesis imperfecta. *Bone* 26: 581–589

Roschger P, Fratzl-Zelman N, Misof BM, Glorieux FH, Klaushofer K, Rauch F (2008) Evidence that abnormal high bone mineralization in growing children with osteogenesis imperfecta is not associated with specific collagen mutations. *Calcif Tissue Int* 82: 263–270

Rýglová Š, Braun M, Suchý T (2017) Collagen and its modifications—crucial aspects with concern to its processing and analysis. *Macromol Mater Eng* 302: 1600460

Sahibdeen V, Crowther NJ, Soodyall H, Hendry LM, Munthali RJ, Hazelhurst S, Choudhury A, Norris SA, Ramsay M, Lombard Z (2018) Genetic variants in SEC16B are associated with body composition in black south Africans. *Nutr Diabetes* 8: 43

Schmidt K, Cavodeassi F, Feng Y, Stephens DJ (2013) Early stages of retinal development depend on Sec13 function. *Biol Open* 2: 256–266

Shaywitz DA, Espenshade PJ, Gimeno RE, Kaiser CA (1997) COPII subunit interactions in the assembly of the vesicle coat. *J Biol Chem* 272: 25413–25416

Supek F, Madden DT, Hamamoto S, Orci L, Schekman R (2002) Sec16p potentiates the action of COPII proteins to bud transport vesicles. *J Cell Biol* 158: 1029–1038

Tillmann KD, Reiterer V, Baschieri F, Hoffmann J, Millarte V, Hauser MA, Mazza A, Atias N, Legler DF, Sharan R *et al* (2015) Regulation of Sec16 levels and dynamics links proliferation and secretion. *J Cell Sci* 128: 670–682

Townley AK, Feng Y, Schmidt K, Carter DA, Porter R, Verkade P, Stephens DJ (2008) Efficient coupling of Sec23-Sec24 to Sec13-Sec31 drives COPII-dependent collagen secretion and is essential for normal craniofacial development. *J Cell Sci* 121: 3025–3034

Van Dijk FS, Sillence DO (2014) Osteogenesis imperfecta: clinical diagnosis, nomenclature and severity assessment. *Am J Med Genet A* 164A: 1470–1481

Wang Y, Xu A, Knight C, Xu LY, Cooper GJ (2002) Hydroxylation and glycosylation of the four conserved lysine residues in the collagenous domain of adiponectin. Potential role in the modulation of its insulin-sensitizing activity. *J Biol Chem* 277: 19521–19529

Wang K, Li M, Hakonarson H (2010) ANNOVAR: functional annotation of genetic variants from high-throughput sequencing data. *Nucleic Acids Res* 38: e164

Watson P, Townley AK, Koka P, Palmer KJ, Stephens DJ (2006) Sec16 defines endoplasmic reticulum exit sites and is required for secretory cargo export in mammalian cells. *Traffic* 7: 1678–1687

Yonekawa S, Furuno A, Baba T, Fujiki Y, Ogasawara Y, Yamamoto A, Tagaya M, Tani K (2011) Sec16B is involved in the endoplasmic reticulum export of the peroxisomal membrane biogenesis factor peroxin 16 (Pex16) in mammalian cells. *Proc Natl Acad Sci USA* 108: 12746–12751

Yuan L, Kenny SJ, Hemmati J, Xu K, Schekman R (2018) TANGO1 and SEC12 are copackaged with procollagen I to facilitate the generation of large COPII carriers. *Proc Natl Acad Sci USA* 115: E12255–E12264

License: This is an open access article under the terms of the Creative Commons Attribution License, which permits use, distribution and reproduction in any medium, provided the original work is properly cited.

Expanded View Figures

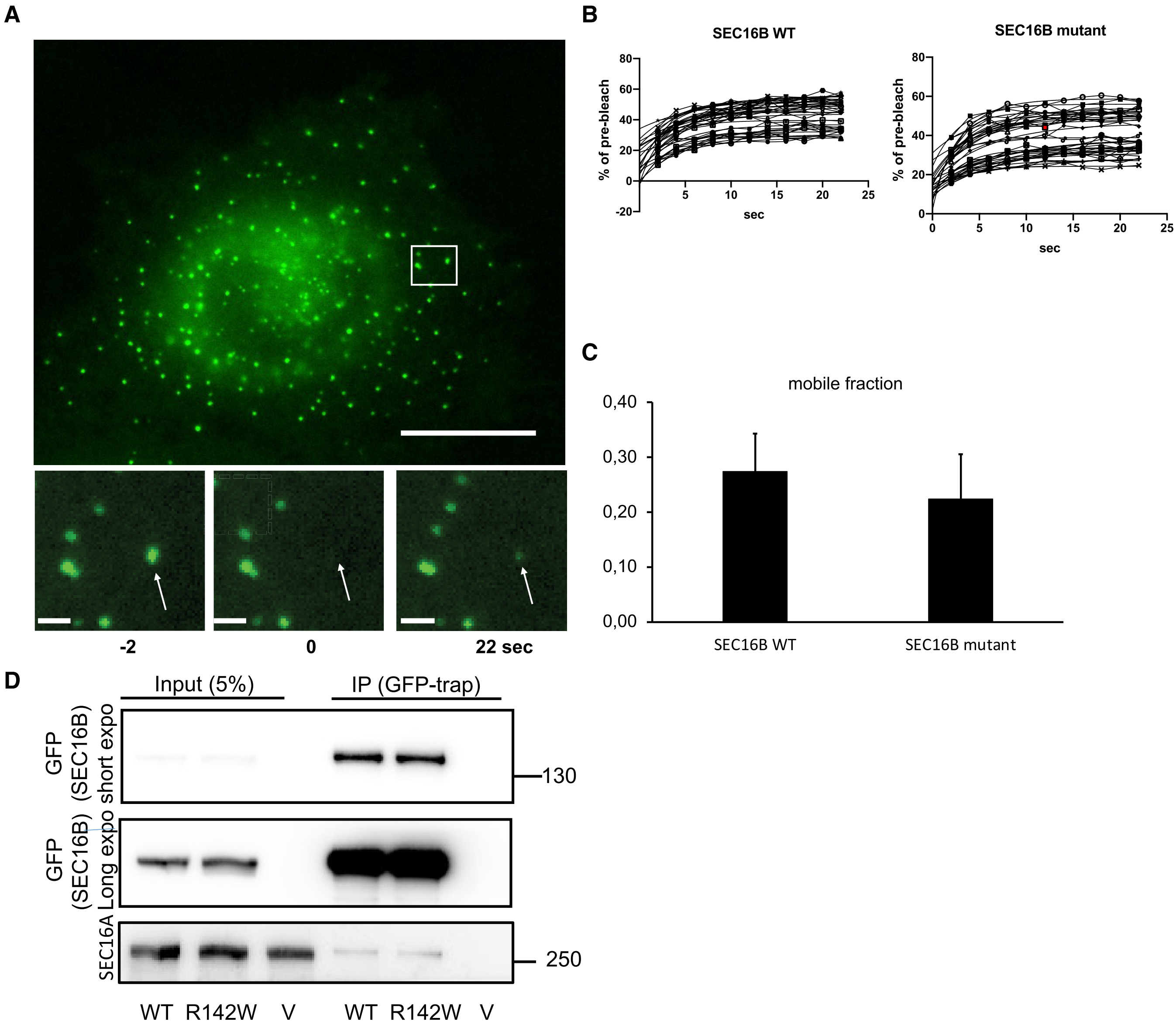

Figure EV1. Characterization of dynamics and interaction of SEC16B mutant.

A HeLa cells were transfected with plasmids encoding wild-type and mutant GFP-SEC16B. After 24 h, FRAP experiment on single ERES was performed on a Nikon CREST V3 spinning disc microscope. The panel shows a representative cell expressing mutant SEC16B with enlarged ERES before and after bleaching. The arrows indicate the position of the bleached ERES. Scale bar in large figure is 10 μm. Scale bar in inserts is 1 μm.

B Quantification of average fluorescence intensities of single ERES. All values were normalized to the pre-bleach fluorescence intensity.

C Calculation of the mobile fractions from the FRAP measurements. Three independent biological experiments. Bars are mean ± SD.

D HeLa cells were transfected with GFP-tagged wild-type or mutant Sec16B. After 24 h, cells were lysed and the lysate was subjected to immunoprecipitation using GFP-tap beads (Chromotek). The immunoprecipitate was immunoblotted against GFP to determine efficiency of the IP, as well as against endogenous Sec16A.

Source data are available online for this figure.

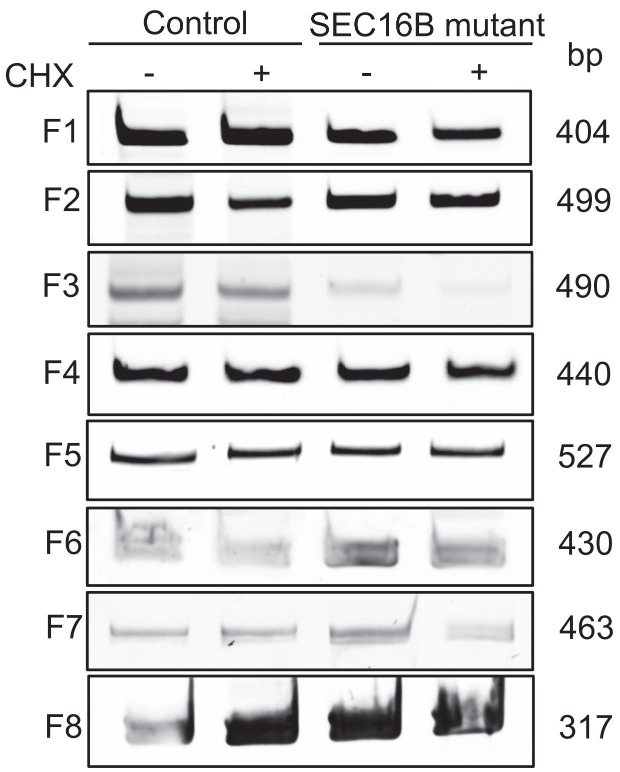

Figure EV2. No evidence of cryptic splicing site in SEC16B open reading frame.

Cells were either left untreated or treated with cycloheximide at concentration of 100 µg/ml for 6 h. gDNA was isolated and PCR was performed using the primer in Table EV4 and sequenced by sanger sequencing. In addition, PCR products were separated on 10% PCR polyacrylamide gel electrophoresis. One representative experiment out of three independent experiments is shown. Biological replicates were performed.

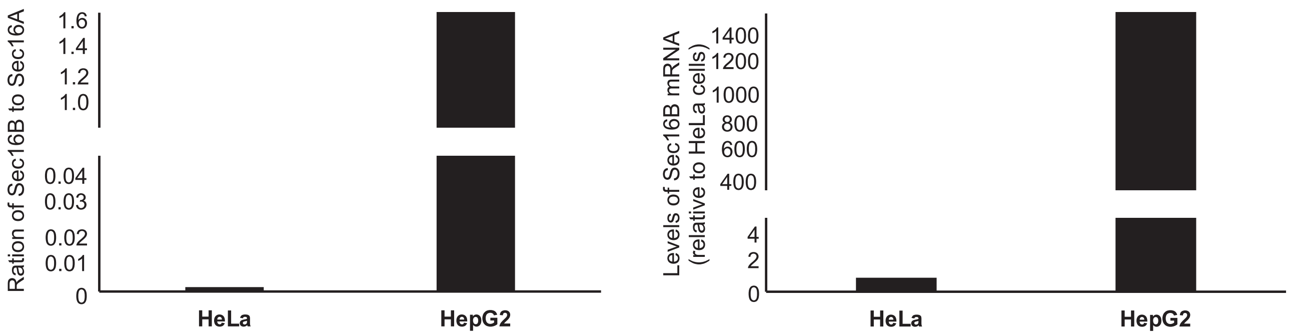

Figure EV3. Relative expression of SEC16A and SEC16B in HeLa and HepG2 cells.

qPCR assessment of mRNA expression level of SEC16B A and SEC16B in HeLa and HepG2 cells. The values are $\Delta\Delta C_T$. HepG2 normalized to HeLa cells. GAPDH was used as housekeeping gene. Bars are from two biological replicates.

 © 2023 The Authors
