# Supplementary figures and images for: Bi‐allelic mutation in SEC16B alters collagen trafficking and increases ER stress
Source: EMBO Mol Med. 2023 Mar 14;15(4):e16834. doi: 10.15252/emmm.202216834 (PMC10086588; doi:10.15252/emmm.202216834)

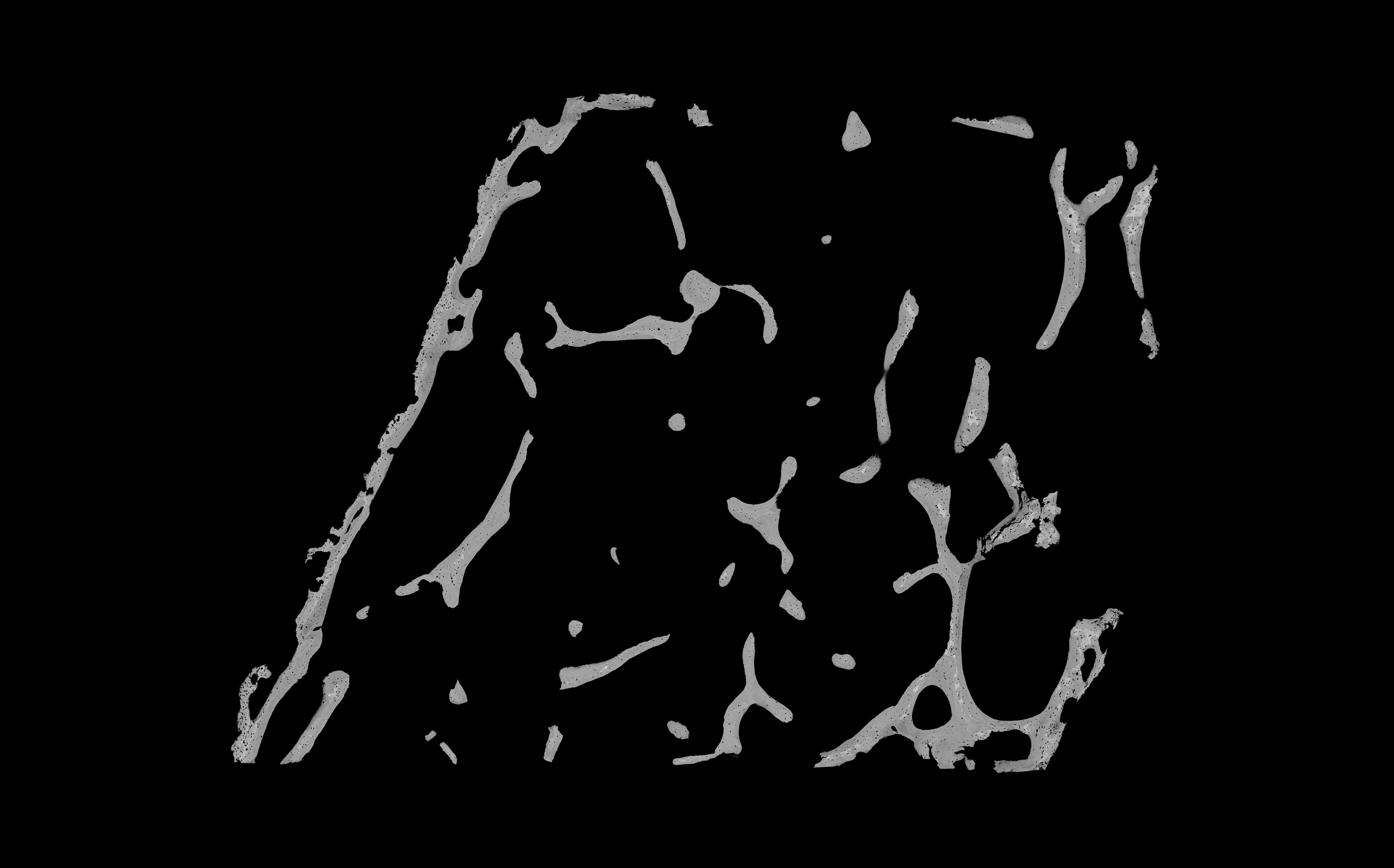

Supplement: Supplementary file 8 — Source Data for Figure 2 [file EMMM-15-e16834-s002.zip › Figure2/2I.tif]

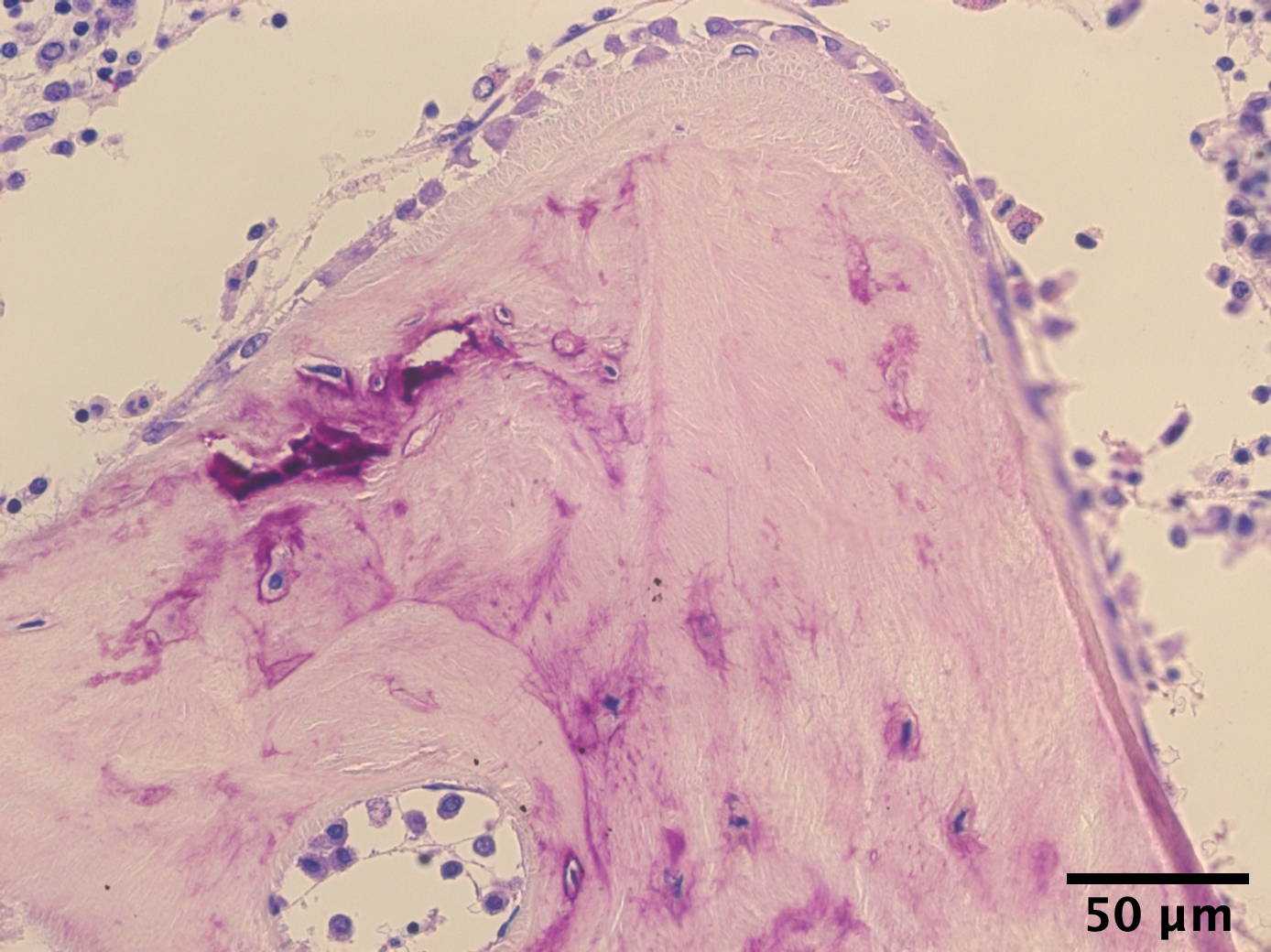

Supplement: Supplementary file 8 — Source Data for Figure 2 [file EMMM-15-e16834-s002.zip › Figure2/2H.tif]

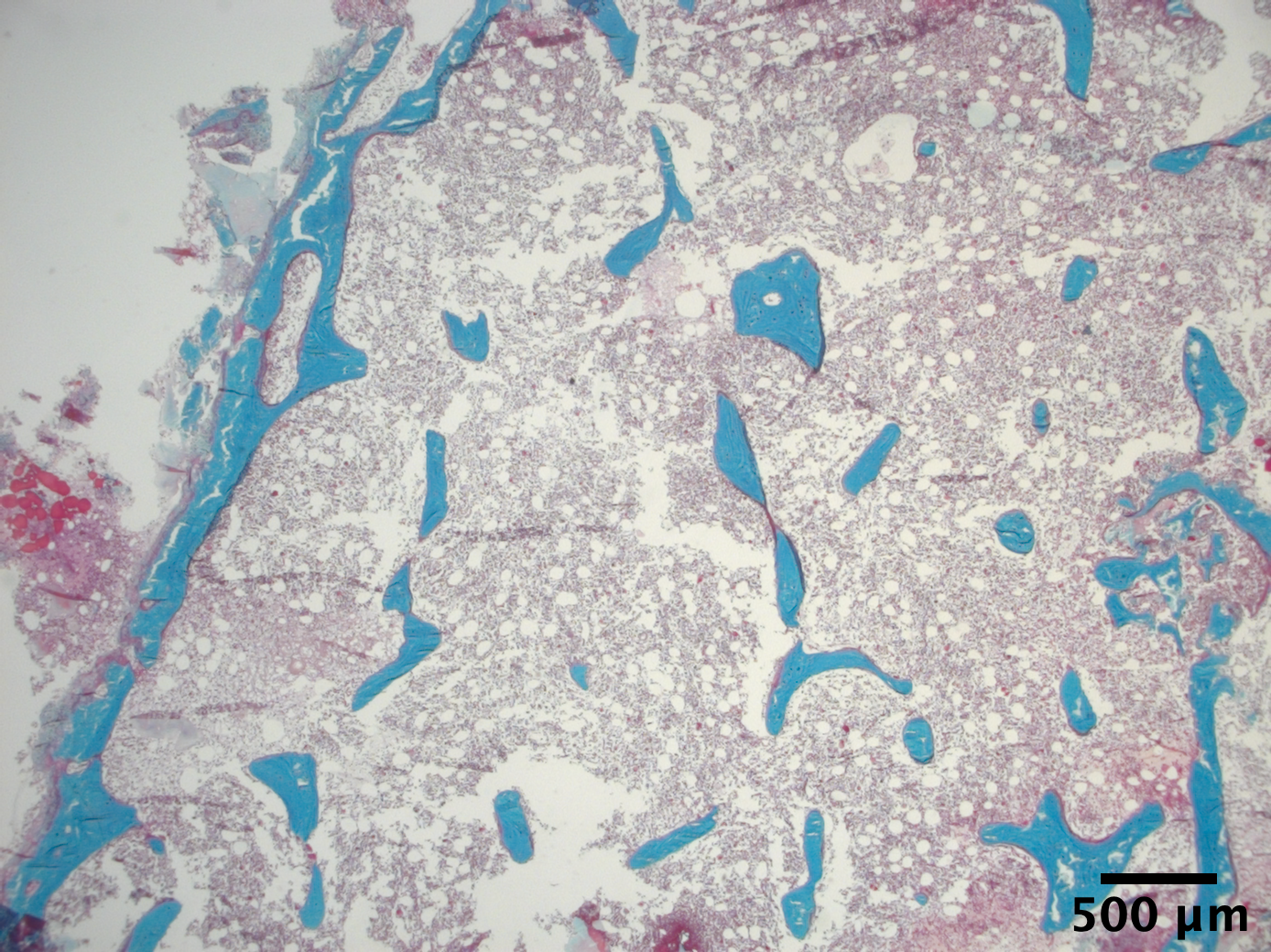

Supplement: Supplementary file 8 — Source Data for Figure 2 [file EMMM-15-e16834-s002.zip › Figure2/2A.tif]

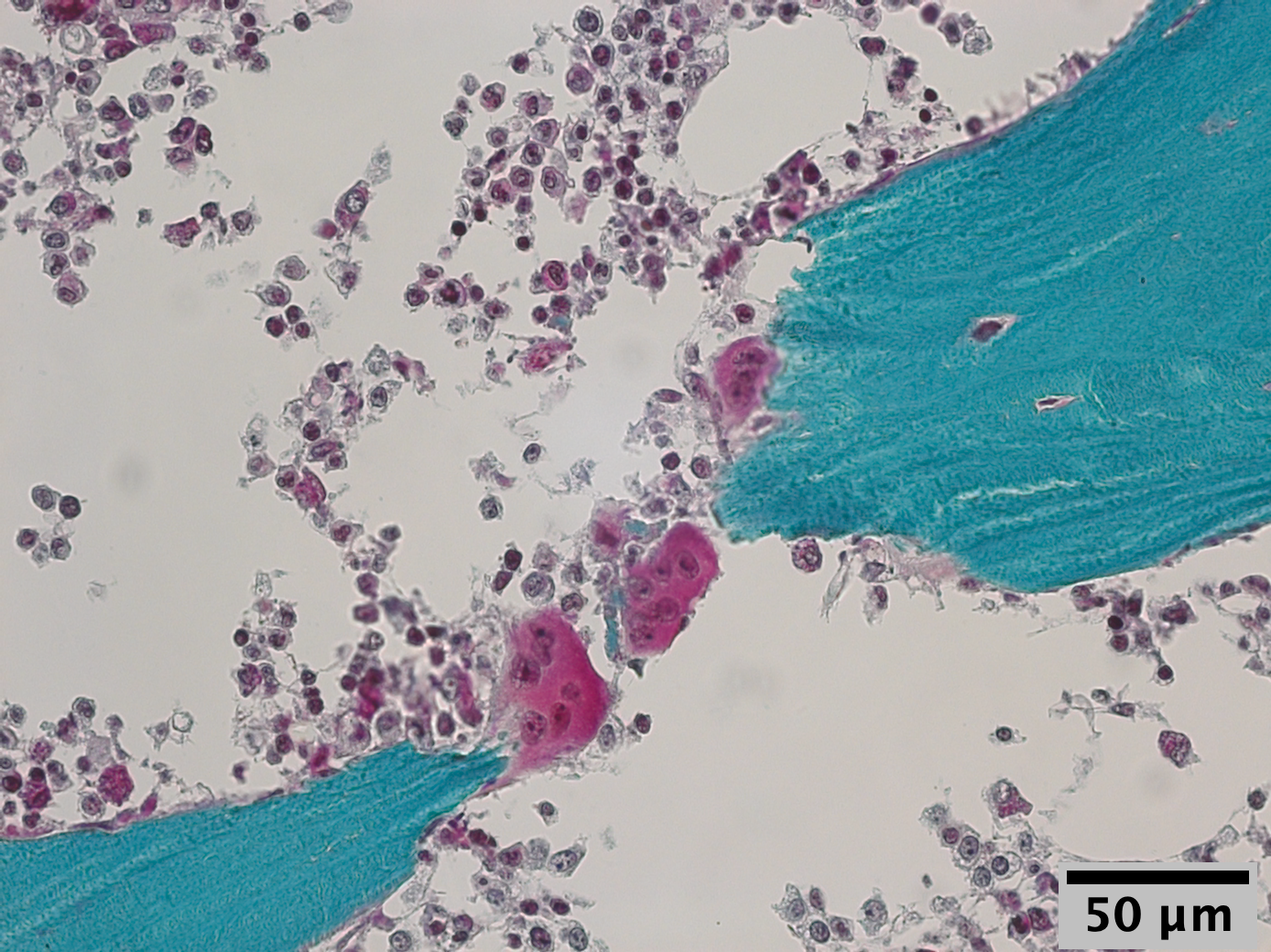

Supplement: Supplementary file 8 — Source Data for Figure 2 [file EMMM-15-e16834-s002.zip › Figure2/2C.tif]

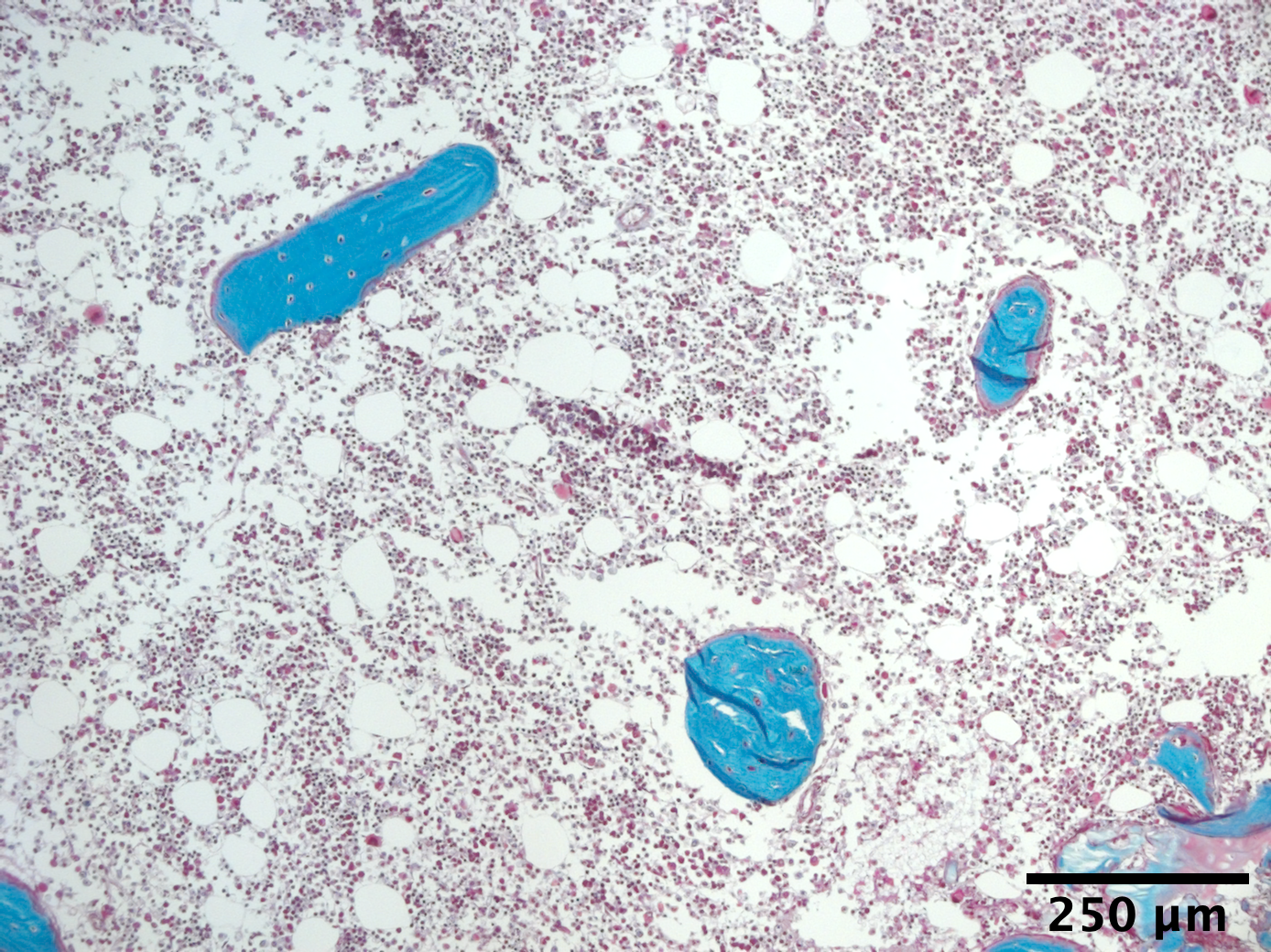

Supplement: Supplementary file 8 — Source Data for Figure 2 [file EMMM-15-e16834-s002.zip › Figure2/2B.tif]

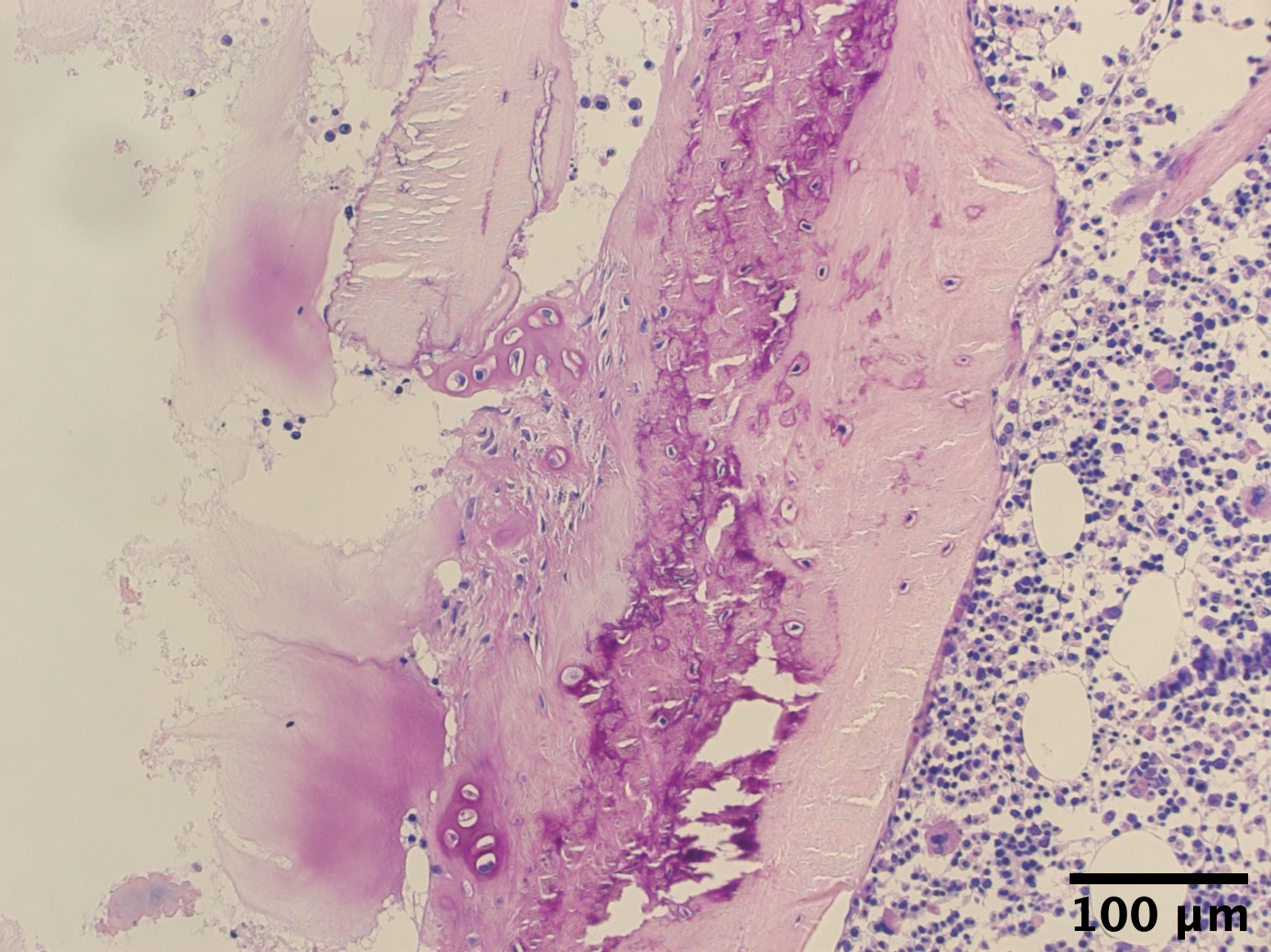

Supplement: Supplementary file 8 — Source Data for Figure 2 [file EMMM-15-e16834-s002.zip › Figure2/2F.tif]

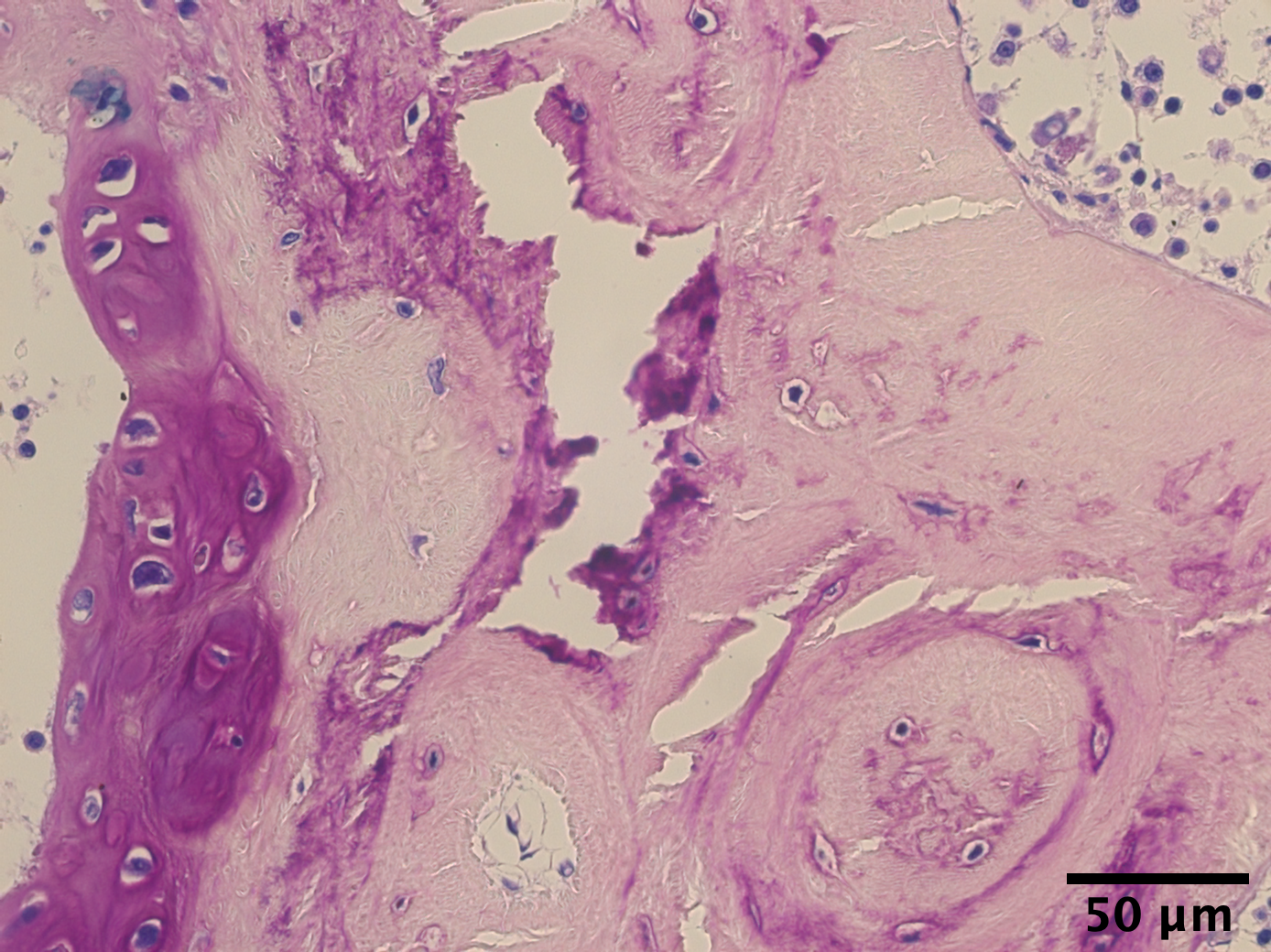

Supplement: Supplementary file 8 — Source Data for Figure 2 [file EMMM-15-e16834-s002.zip › Figure2/2G.tif]

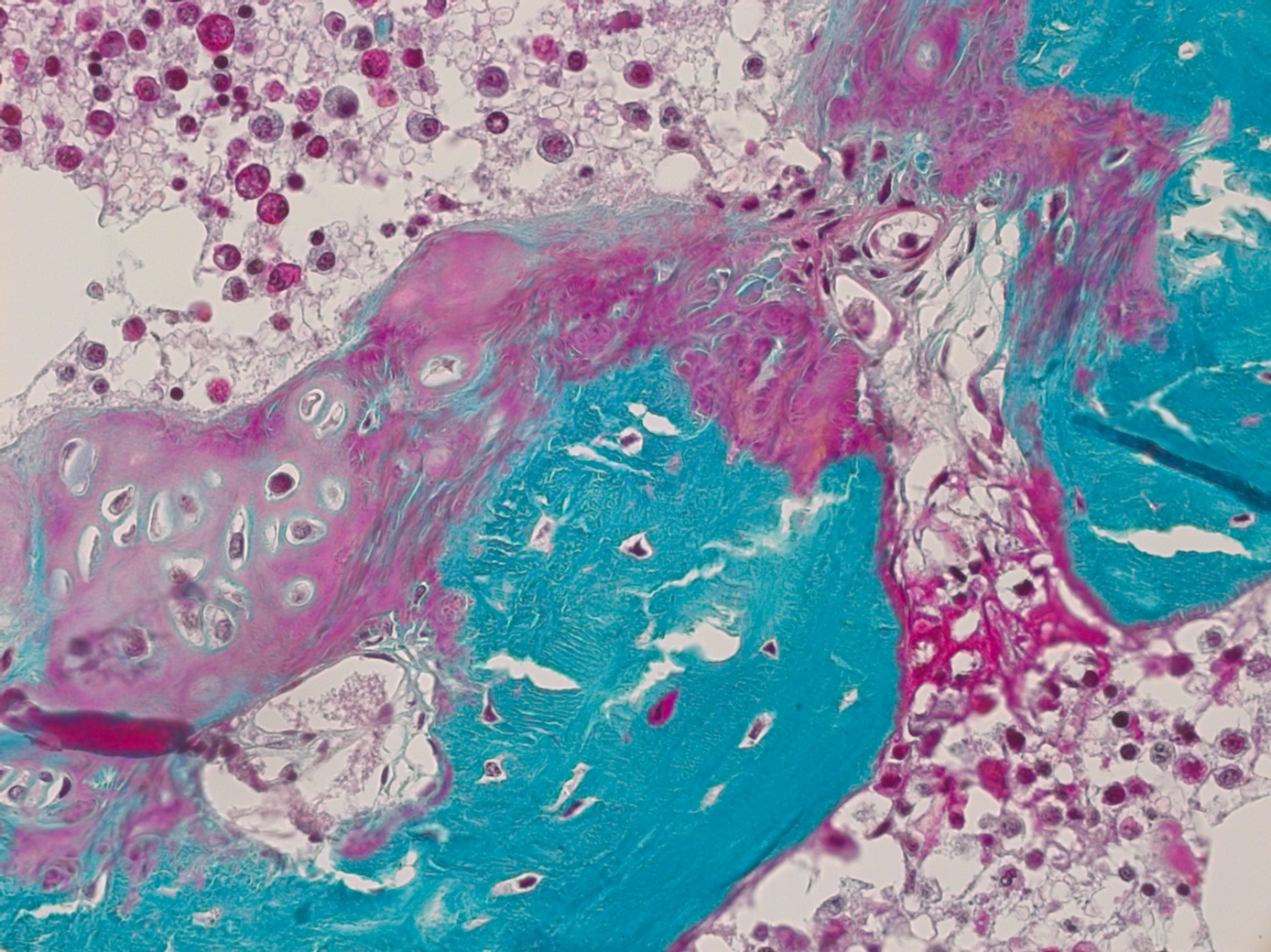

Supplement: Supplementary file 8 — Source Data for Figure 2 [file EMMM-15-e16834-s002.zip › Figure2/2E.tif]

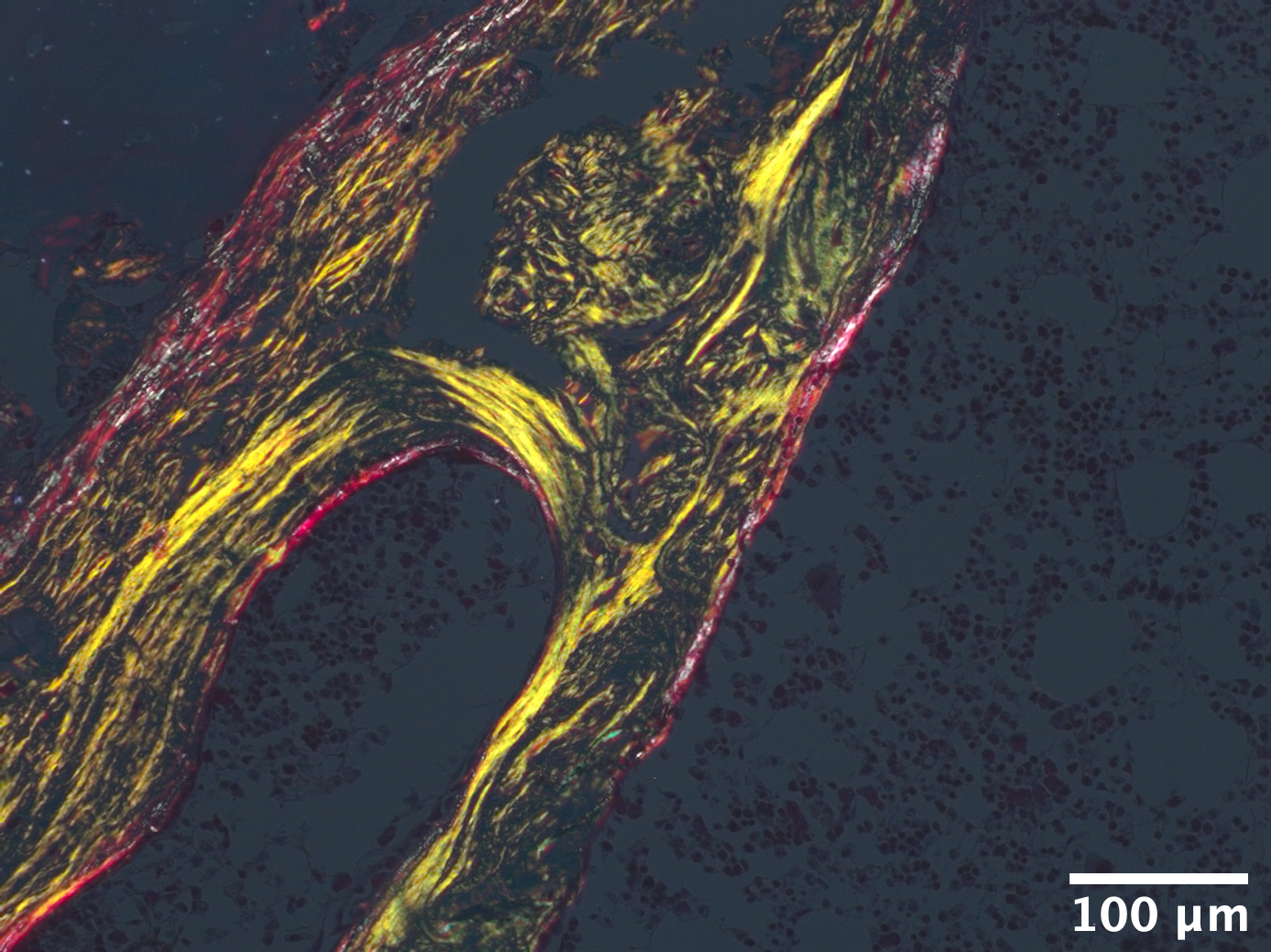

Supplement: Supplementary file 8 — Source Data for Figure 2 [file EMMM-15-e16834-s002.zip › Figure2/2D.tif]

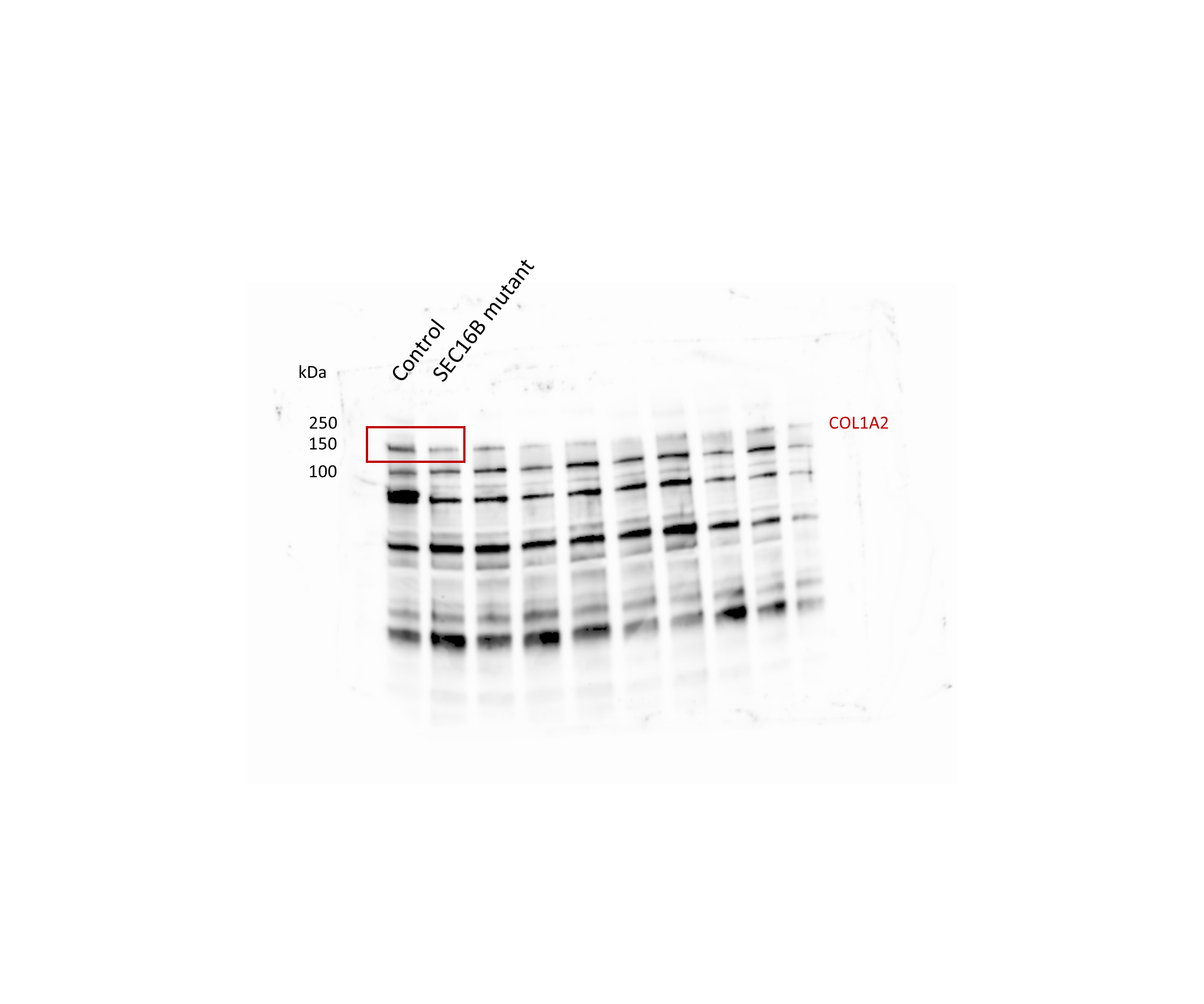

Supplement: Supplementary file 9 — Source Data for Figure 4 [file EMMM-15-e16834-s014.zip › Figure4/4E/4E_COL1A2.TIF]

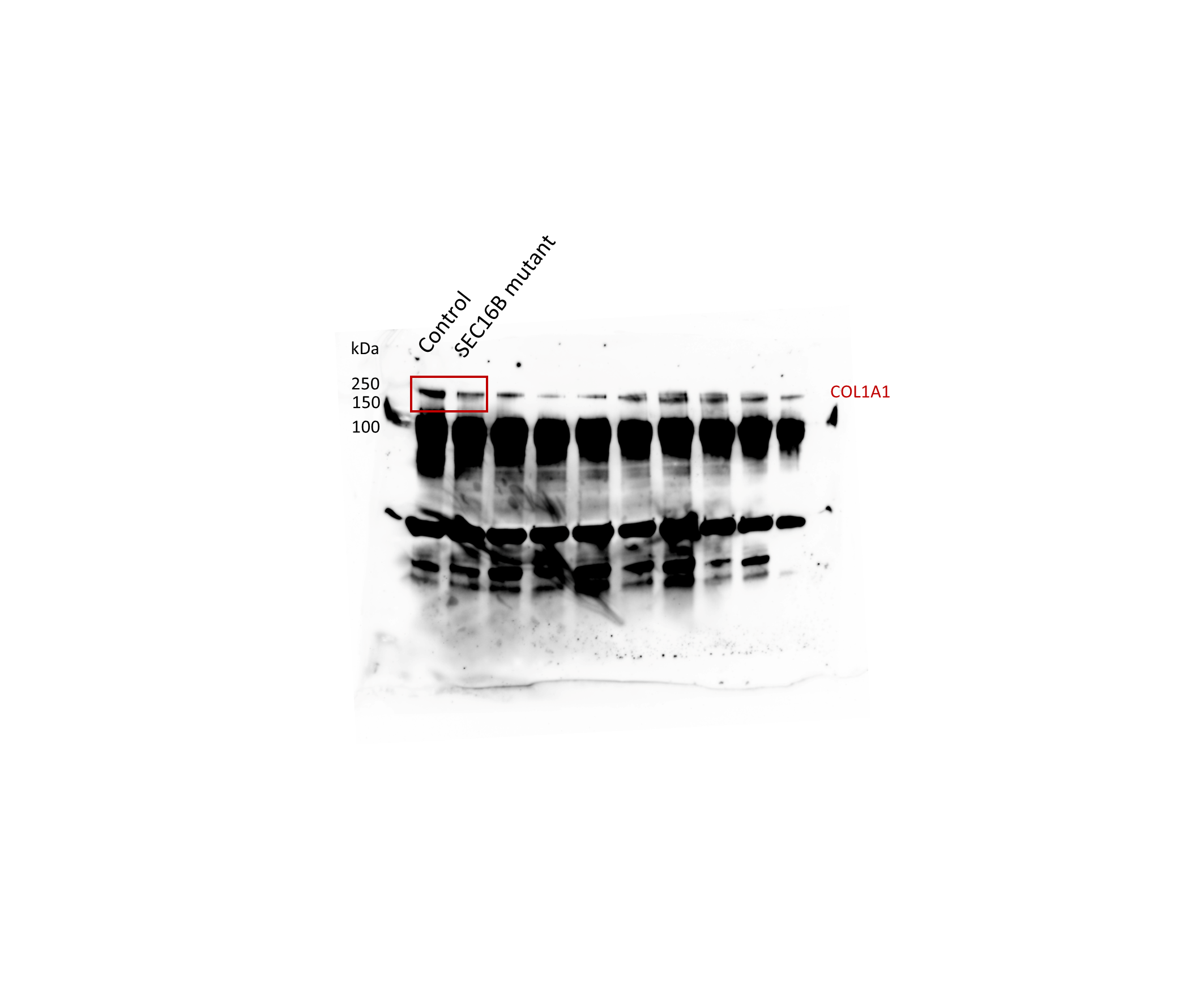

Supplement: Supplementary file 9 — Source Data for Figure 4 [file EMMM-15-e16834-s014.zip › Figure4/4E/4E_COL1A1.TIF]

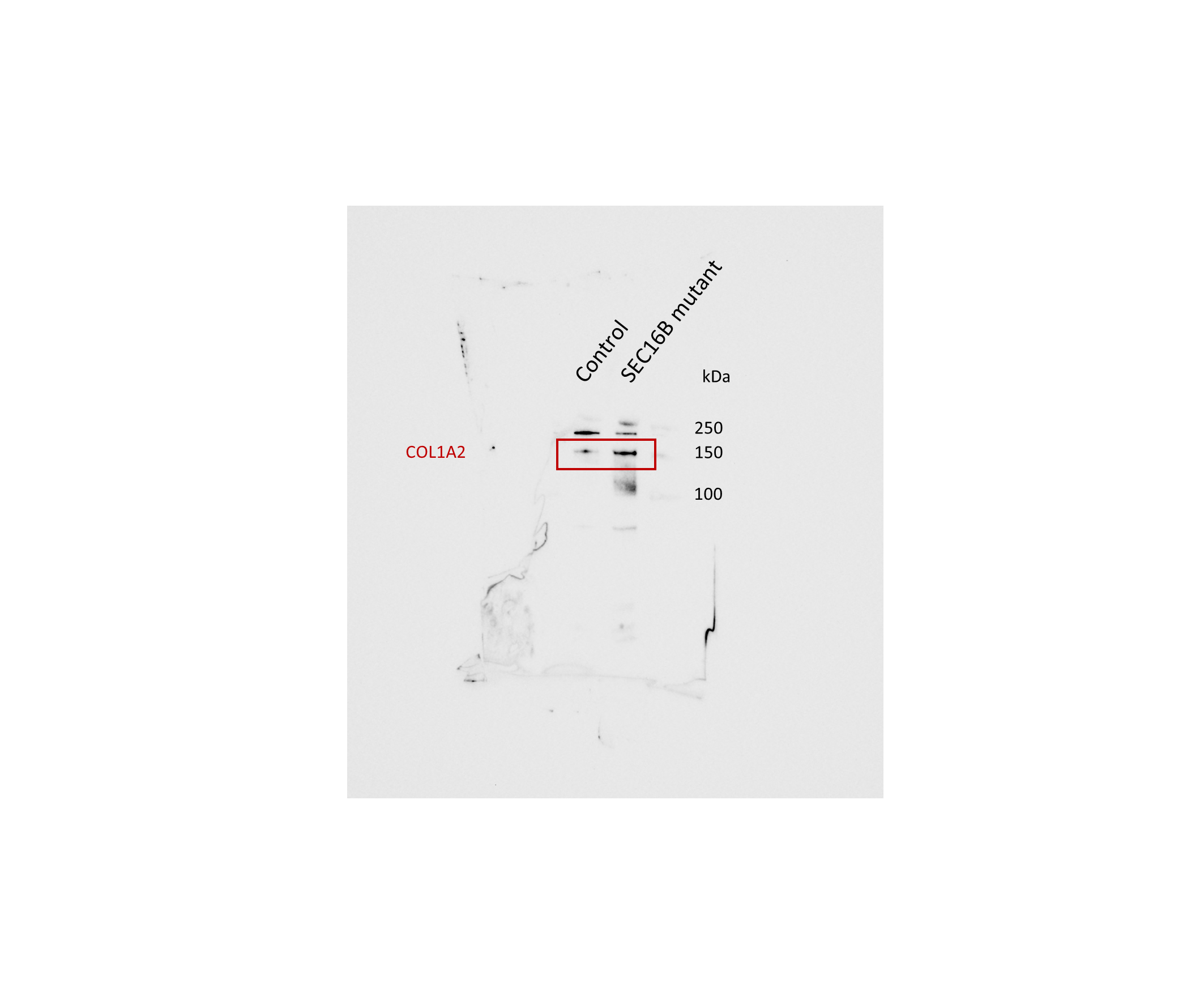

Supplement: Supplementary file 9 — Source Data for Figure 4 [file EMMM-15-e16834-s014.zip › Figure4/4C/4C_CO1A2.TIF]

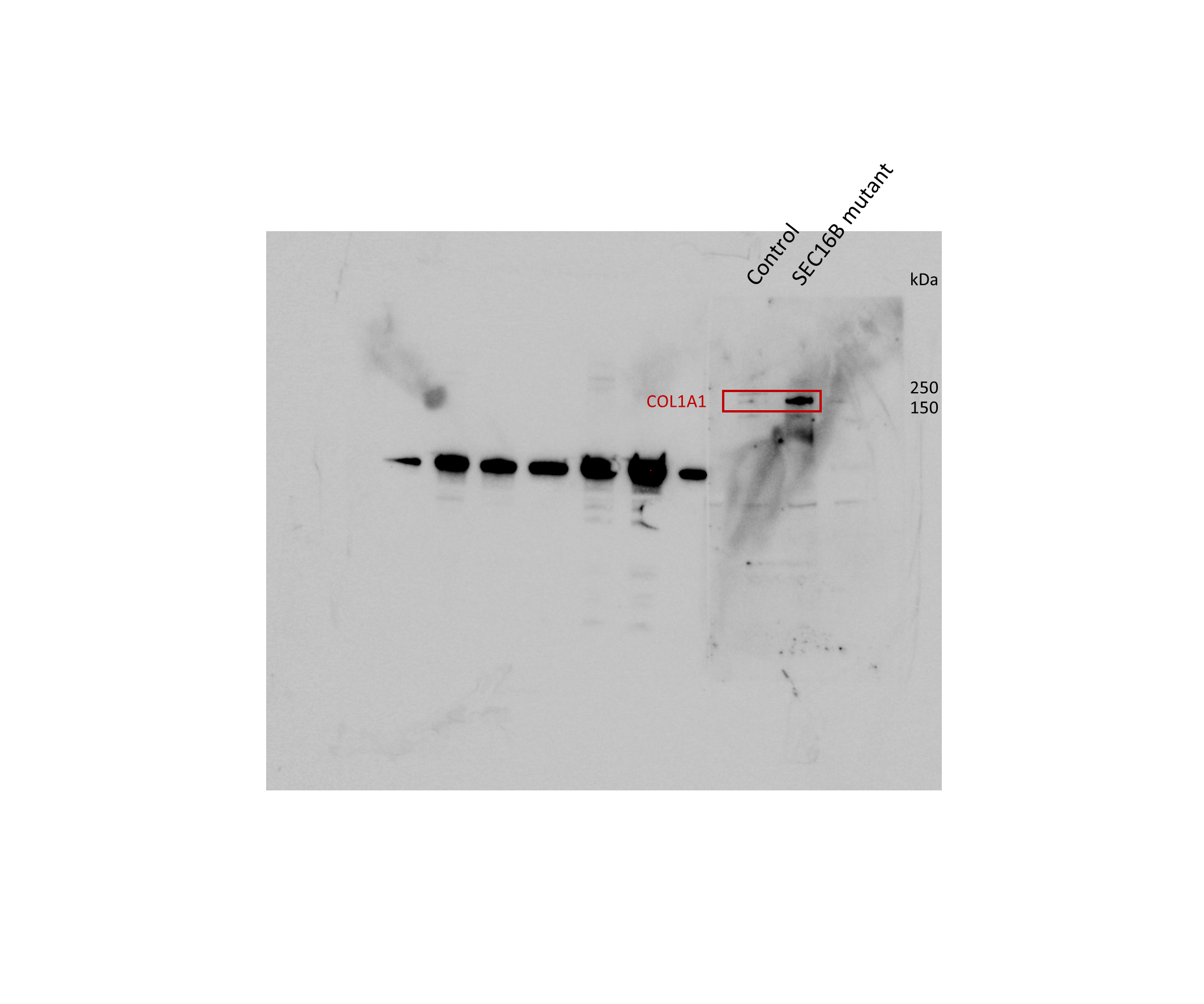

Supplement: Supplementary file 9 — Source Data for Figure 4 [file EMMM-15-e16834-s014.zip › Figure4/4C/4C_COL1A1.TIF]

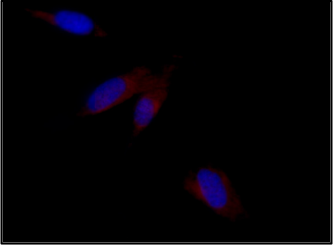

Supplement: Supplementary file 9 — Source Data for Figure 4 [file EMMM-15-e16834-s014.zip › Figure4/4A/4A_control_proalpha1_red.tif]

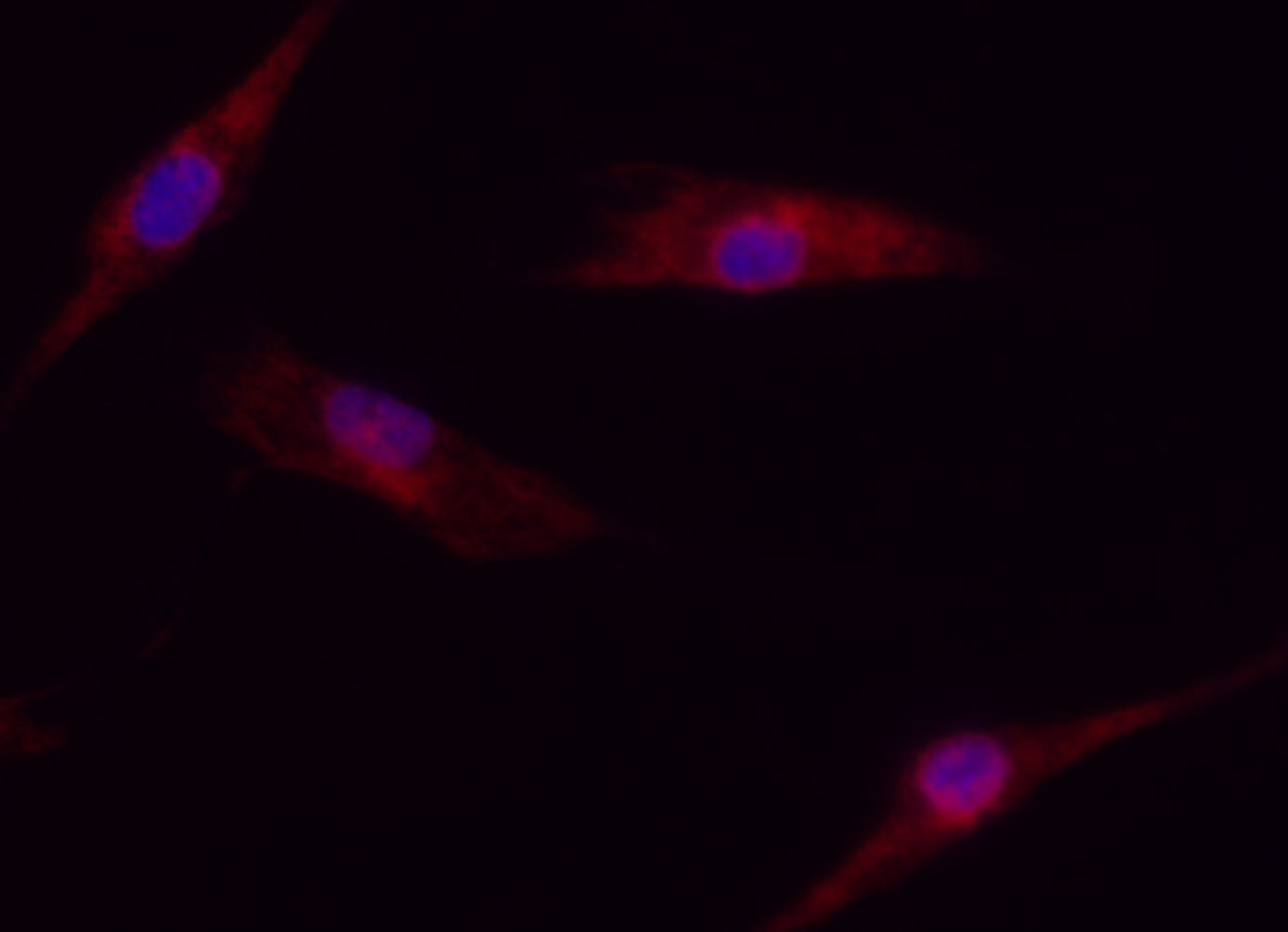

Supplement: Supplementary file 9 — Source Data for Figure 4 [file EMMM-15-e16834-s014.zip › Figure4/4A/4A_SEC16B_mutant_proalpha1_red.tif]

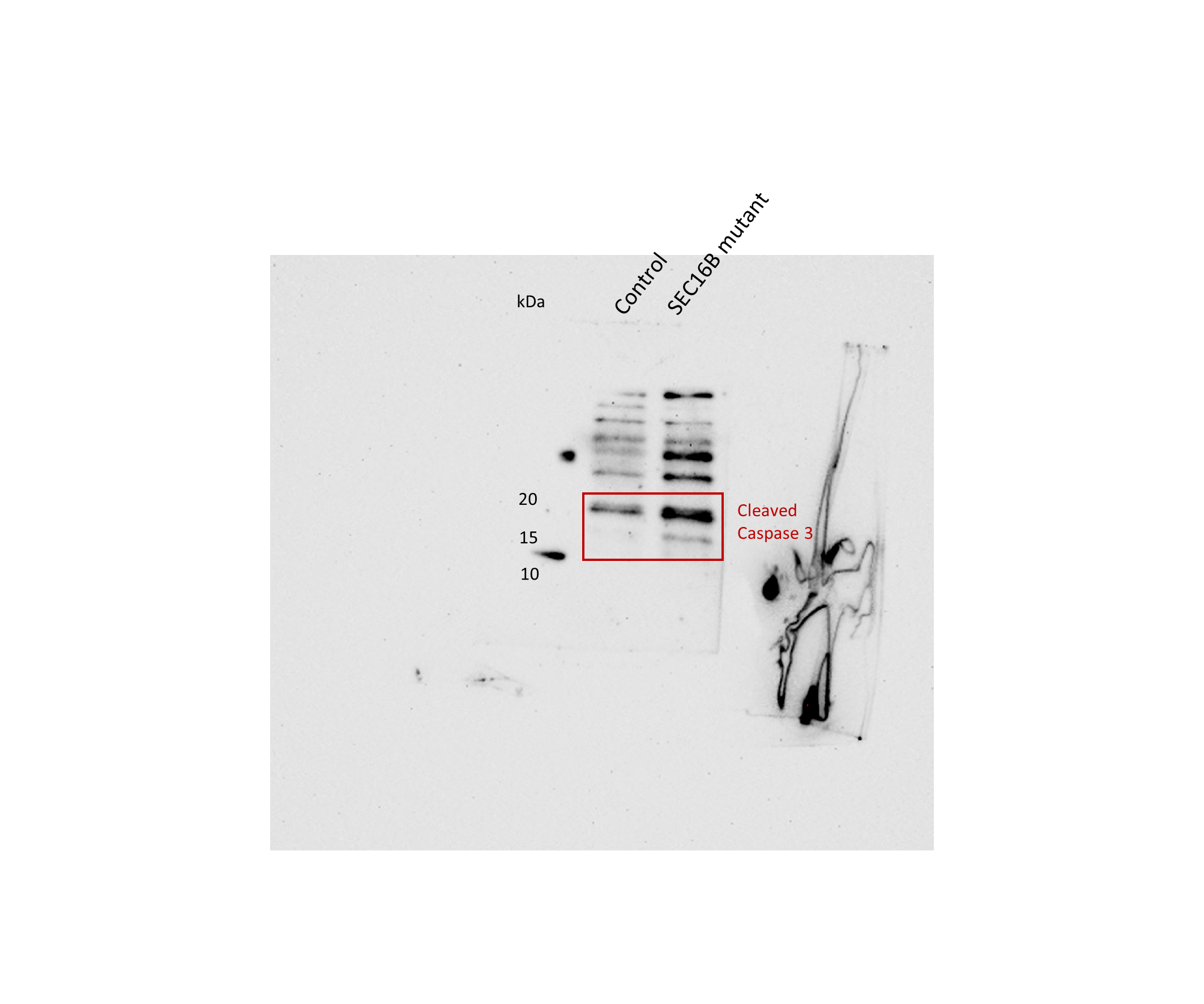

Supplement: Supplementary file 11 — Source Data for Figure 6 [file EMMM-15-e16834-s013.zip › Figure6/6F/6F.TIF]

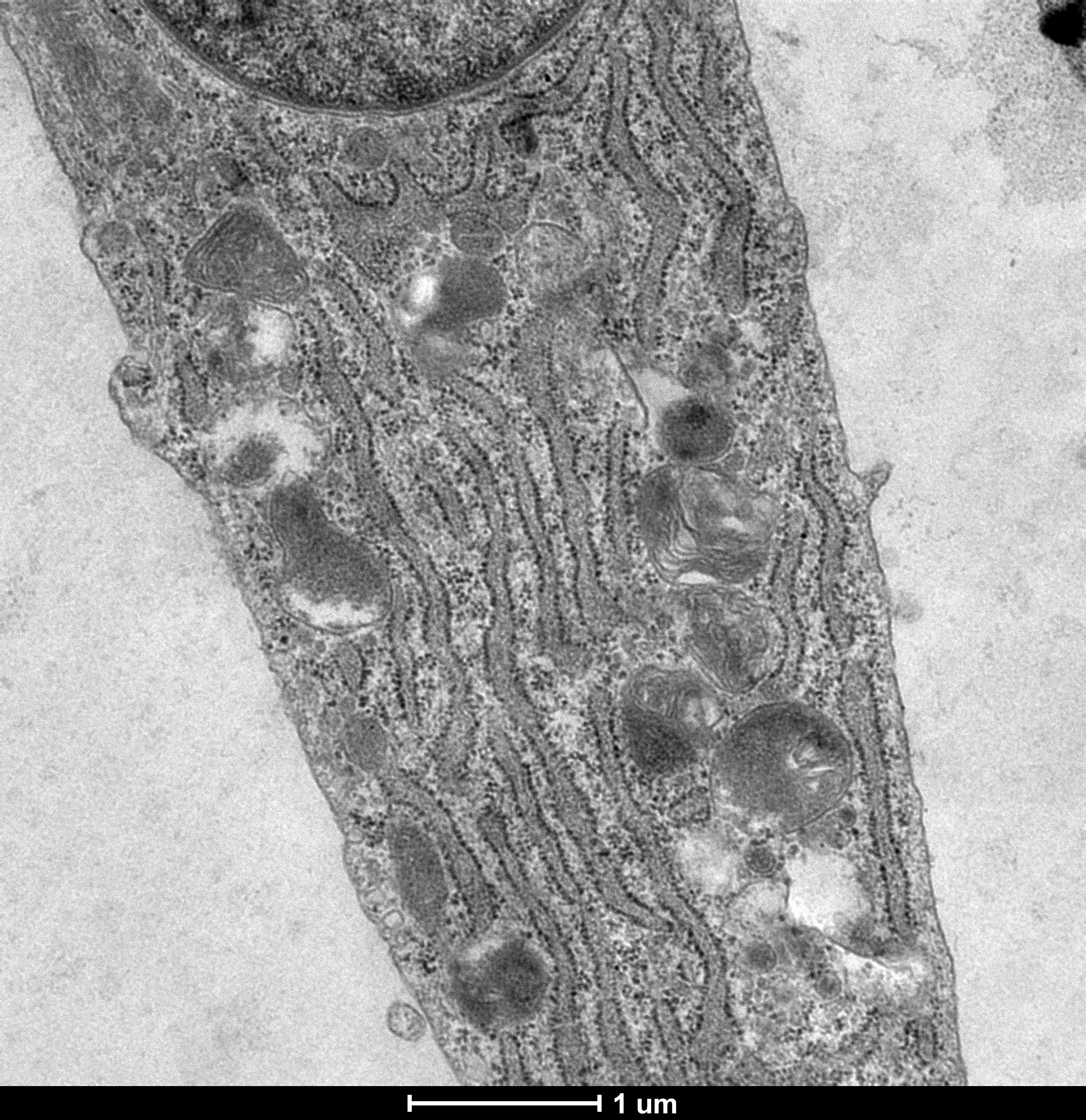

Supplement: Supplementary file 11 — Source Data for Figure 6 [file EMMM-15-e16834-s013.zip › Figure6/6A/6A_SEC16B_mutant.tif]

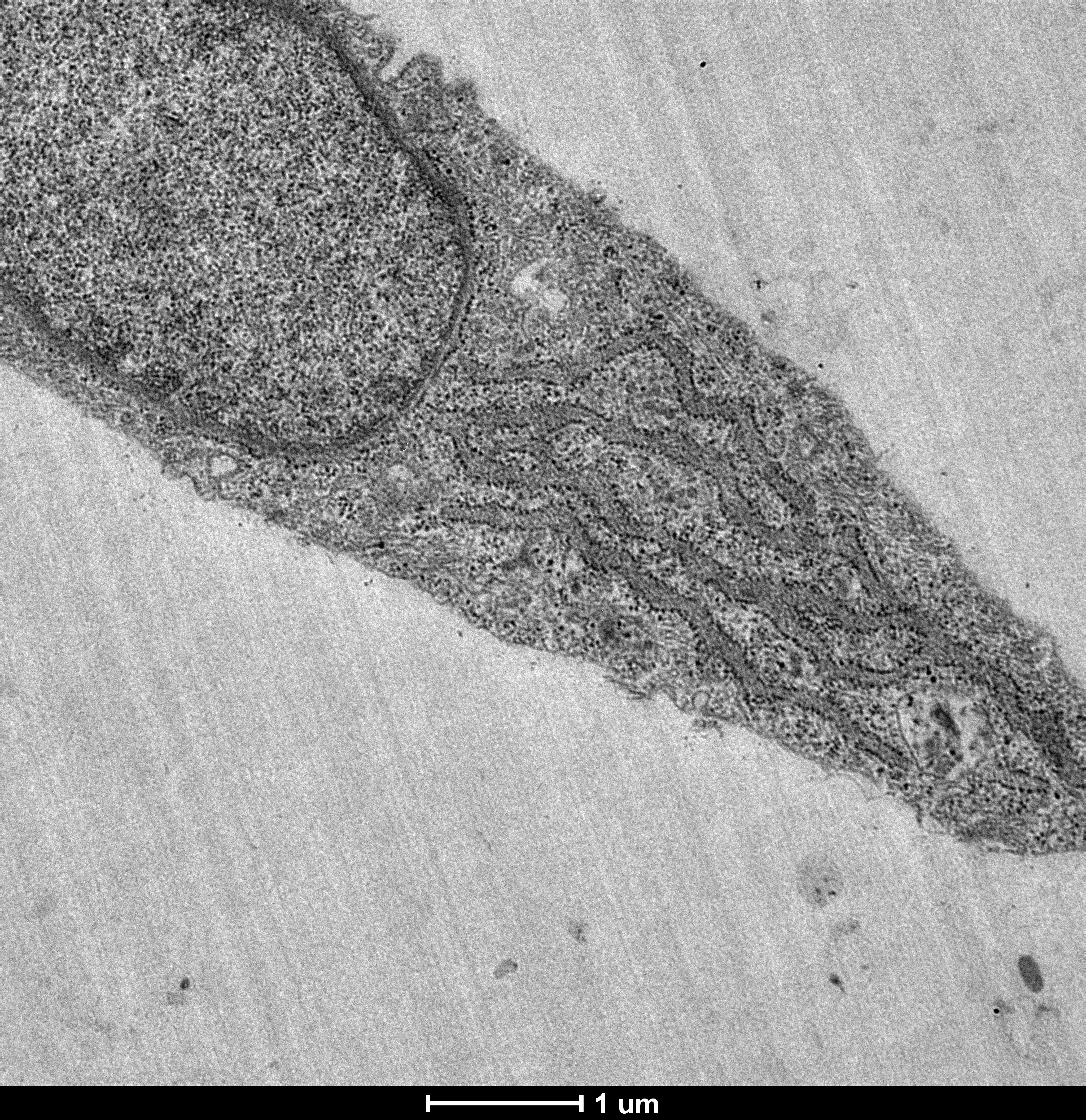

Supplement: Supplementary file 11 — Source Data for Figure 6 [file EMMM-15-e16834-s013.zip › Figure6/6A/6A_control.tif]

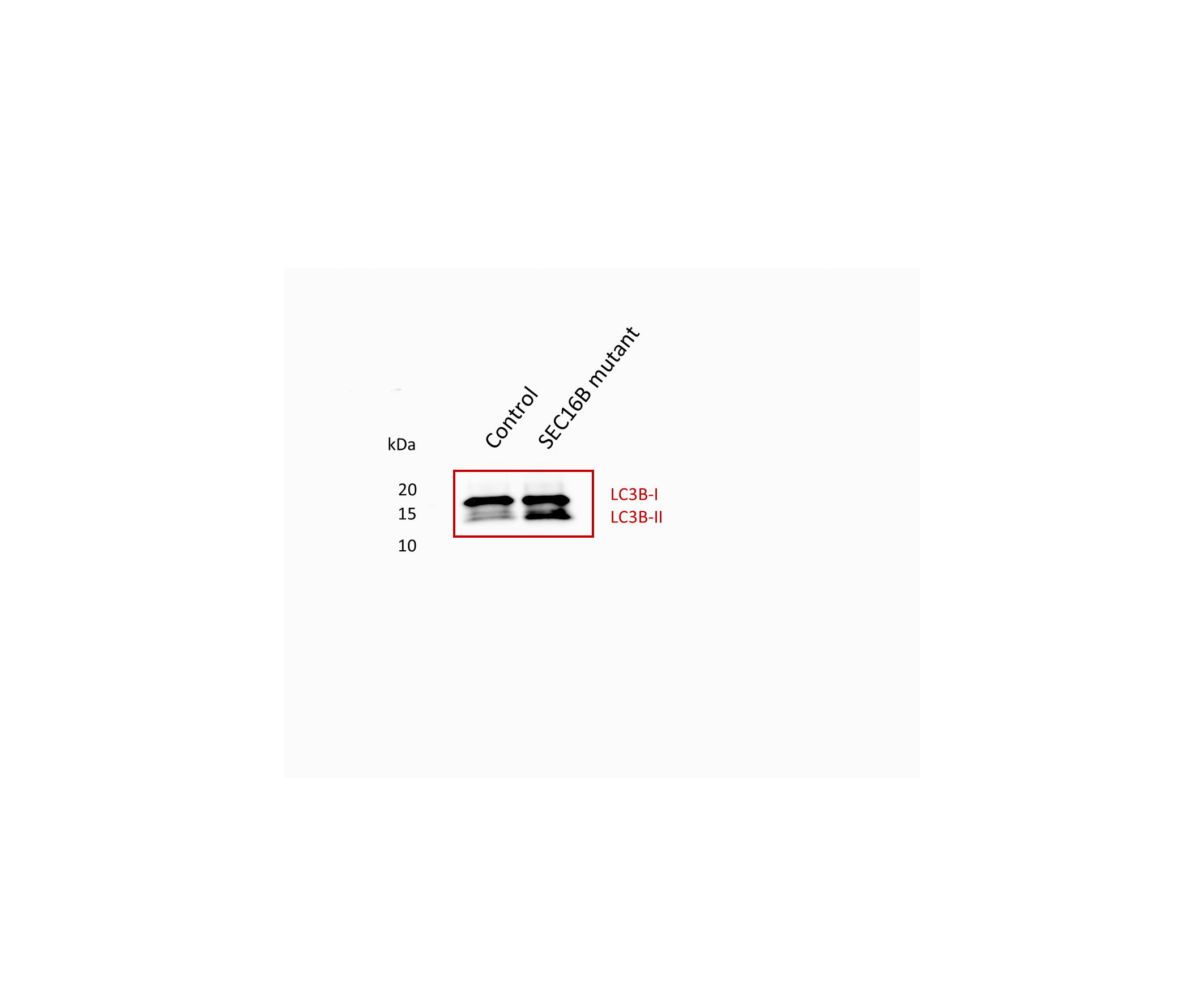

Supplement: Supplementary file 11 — Source Data for Figure 6 [file EMMM-15-e16834-s013.zip › Figure6/6C/6C_LC3B.TIF]

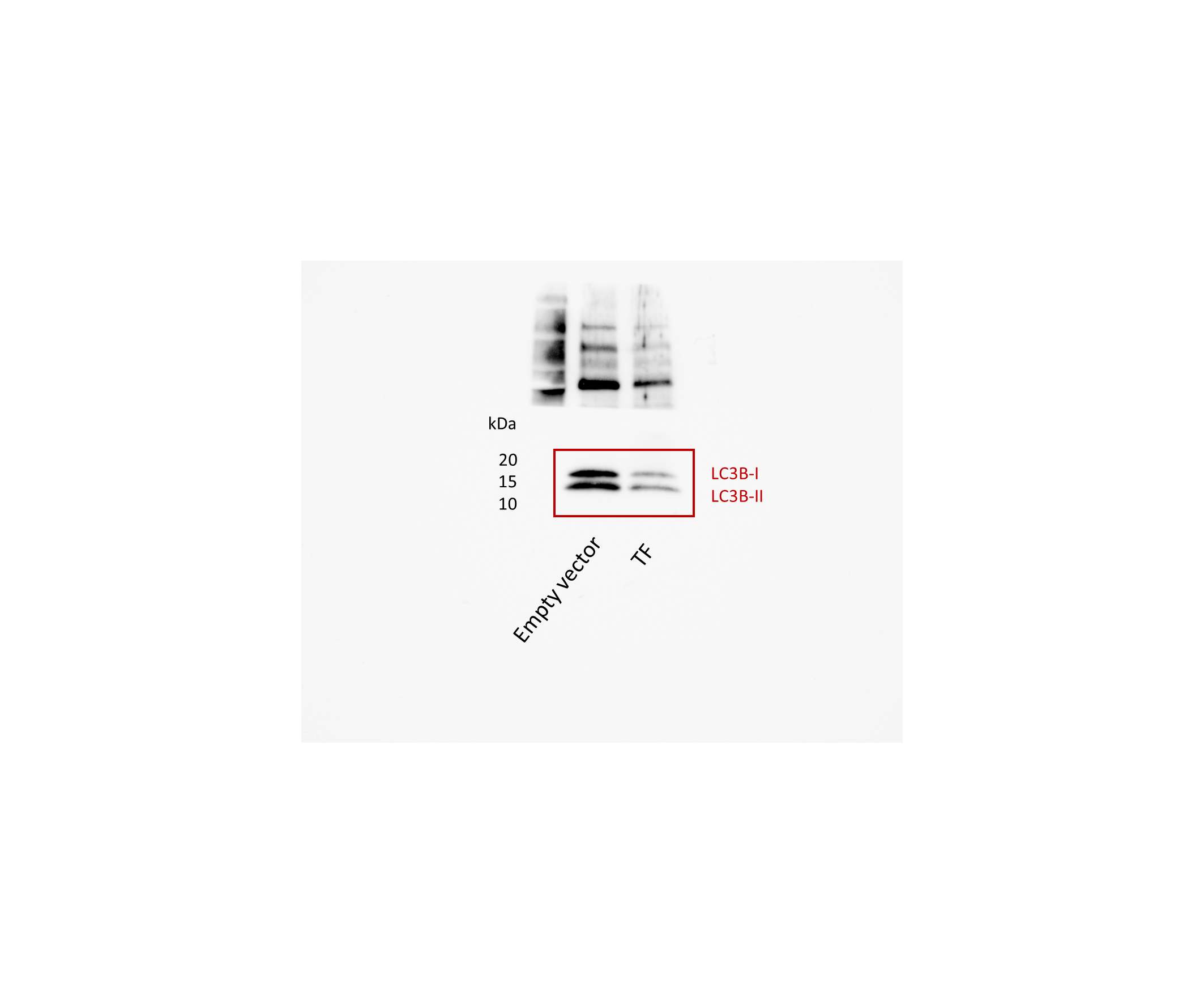

Supplement: Supplementary file 12 — Source Data for Figure 7 [file EMMM-15-e16834-s007.zip › Figure7/7D/7D.TIF]
