# Supplementary material for: Bi‐allelic mutation in SEC16B alters collagen trafficking and increases ER stress
Source: EMBO Mol Med. 2023 Mar 14;15(4):e16834. doi: 10.15252/emmm.202216834 (PMC10086588; doi:10.15252/emmm.202216834)
Supplement: Supplementary file 14 [file EMMM-15-e16834-s005.pdf]

EMBO Molecular Medicine

Bi-allelic mutation in SEC16B alters collagen trafficking and increases ER stress

Ahmed El-Gazzar, Barbara Voraberger, Frank Rauch, Mario Mairhofer, Katy Schmidt, Brecht Guillemyn, Goran Mitulović, Veronika Reiterer, Margot Haun, Michaela Mayr, Johannes Mayr, Susanne Kimeswenger, Oliver Drews, Vrinda Saraff, Nick Shaw, Nadja Fratzl-Zelman, Sofie Symoens, Hesso Farhan, and Wolfgang Högler
DOI: 10.15252/emmm.202216834

Corresponding author: Wolfgang Högler (wolfgang.hoegler@kepleruniklinikum.at)

Review Timeline:

Submission Date:	2nd Sep 22
Editorial Decision:	4th Oct 22
Revision Received:	13th Jan 23
Editorial Decision:	1st Feb 23
Revision Received:	15th Feb 23
Accepted:	17th Feb 23

Editor: Zeljko Durdevic

Transaction Report:

(Note: With the exception of the correction of typographical or spelling errors that could be a source of ambiguity, letters and reports are not edited. Depending on transfer agreements, referee reports obtained elsewhere may or may not be included in this compilation. Referee reports are anonymous unless the Referee chooses to sign their reports.)

4th Oct 2022

Dear Prof. Högler,

Thank you for the submission of your manuscript to EMBO Molecular Medicine. We have now received feedback from the three reviewers who agreed to evaluate your manuscript. As you will see from the reports, all three referees recognize potential interest of the study but also raise seriuos and partially overlapping concerns, particularly regarding the lack of animal model to support main conclusions of the study. During our cross-commenting session referees agreed that without an animal model the conclusion that SEC16B is a new gene causing osteogenesis imperfecta (OI) has to be toned down. Therefore, we would like to invite major revisions, which would either include addition of an animal model (e.g., gene knockdown in zebrafish that could be done relatively fast) or toning down the conclusions and adjusting the title of the manuscript regarding SEC16B mutation as a cause of OI.

Further consideration of a revision that addresses reviewers' concerns in full will entail a second round of review. EMBO Molecular Medicine encourages a single round of revision only and therefore, acceptance or rejection of the manuscript will depend on the completeness of your responses included in the next, final version of the manuscript. For this reason, and to save you from any frustrations in the end, I would strongly advise against returning an incomplete revision.

We would welcome the submission of a revised version within six months for further consideration. Please let us know if you require longer to complete the revision.

Please use this link to login to the manuscript system and submit your revision: https://embomolmed.msubmit.net/cgi-bin/main.plex

I look forward to receiving your revised manuscript.

Yours sincerely,

Zeljko Durdevic

Zeljko Durdevic
Editor
EMBO Molecular Medicine

/**** Reviewer's comments *****

Referee #1 (Comments on Novelty/Model System for Author):

There is no model involved in this manuscript. IT is the description of a patient with a skeletal dysplasia and the associated radiographic, histological, genetic and molecular findings. The data are both novel and of potential high impact.

Referee #1 (Remarks for Author):

Summary: the authors provide radiographic, histological, genetic, and molecular data on a young male patient affected with a form of skeletal dysplasia manifesting with vertebral fractures, bowing of the lower limbs, short stature, muscle hypotonia and histomorphometric features similar to OI. They identified a homozygous missense mutation in the SEC16B gene (p.R142W) which affected intracellular collagen trafficking, caused ER-stress, increased autophagosome formation and apoptosis in primary skin fibroblasts compared to healthy control fibroblasts. Transfection of a WT SEC16B protein into the patient's fibroblasts rescued the collagen trafficking defect. The missense mutation did not affect its location or function but rather appeared to affect its own expression level by reducing the amount of SEC16B mRNA.

Comments: This interesting and well-conducted study appears to finally shed light on the function of the SEC16B protein. The conclusion is that in cell with a high secretory demand, expression levels of SEC16B are similar to those of SEC16A and the former is important for the ER to GOLGI trafficking of large cargo proteins, such as type I collagen. Therefore, SEC16B mutations affect collagen trafficking and cause a skeletal dysplasia reminiscent of a recessive form of OI. I have the following specific comments:

1. TITLE. Since the authors have identified only 1 patient in 1 family with this genetic mutation, since the underlying skeletal dysplasia shows some unique features such as vertebral compression fractures only and no fractures in long bones, muscle hypotonia, and histological findings of mineralized cartilage remnants which are less typical of OI, and the authors themselves (in the Discussion - line 369) suggest SEC16B as a new candidate gene for recessive OI, I would recommend to change the title into something less affirmative and more suggestive of a potential new candidate gene for OI. After all, a second patient with mutations in SEC16B may present with a different phenotype, so I feel it is a bit premature to state that SEC16B is a new OI gene.
2. Abstract: same comment as above for the final sentence (lines 76-77). Also, there is a typo in the verb (cause).
3. Introduction: here the authors properly state in the last sentence that 'SEC16B is a candidate gene in OI'.
4. Results: line 214 - it appears from Fig. 4 C,D that primarily COL1A1 is retained in the ER and much less COL1A2. Perhaps normalization to PDI levels (instead of calnexin) in Figure D would provide a better evidence for the concomitant retention of COL1A2?

5. Lines 220-221: the statement that SEC16B was proposed to exert its function at ERES....needs references to support it.

6. Lines 229-236: I am confused about the mass spectrometric findings from the secreted collagen. How is it possible that only certain peptides were detected in one genotype and not in the other one? How do the authors explain this? I could understand the lack of a post-translational modification (e.g. a phosphorylation) on one peptide in the patient vs the control collagen but how to explain the lack of entire peptides? Was the collagen fragmentation incomplete? This was rather confusing and the statement in the discussion that collagen from the patient cells was differentially modified is not supported by the data as is. Please explain.
7. Line 314: please keep using past tense when discussing the results (we were unable to).
8. Line 321: the finding of a 70% reduction of SEC16B mRNA in the patient's fibroblasts is quite interesting but there is no discussion on how this could happen. After all this is a missense mutation, so how could the mutant protein affect the transcription of its own coding gene? There should be some discussion about how this could happen.
9. Line 330: I would not say that the decrease was 'marked' as it appears mild from the provided figure 8 D,E.
10. If there is a collagen trafficking defects, is there ultimately a reduced amount of collagen outside the cells? A simple collagen staining of the ECM deposed by these fibroblasts could provide an answer to this. Also, a collagen preparation from the media of these cells could show a delayed electrophoretic migration indicating indeed altered PTMs. Were these experiments performed?
11. Line 371: there is a typo - 'which has the closest homology to...'
12. Final statement - Lines 410-414: I would say that 'the mutation results in lower SEC16B mRNA levels...' and fix the statement on COL1A1 PTM since these were not demonstrated.
13. Table 1: please indicate what the numbers in brackets are. Are these SD or else?
14. Legend to Figure 5: Quantification of the number of ERES per area from cells processed as in panel E. Panel E from what figure?

Referee #2 (Remarks for Author):

The authors present an exciting case of a homozygous SEC16B mutation as cause for osteogenesis imperfecta. In line with the involvement of SEC16B in anterograde transport, the functional analyses showed trafficking defects including accumulation of collagen in the ER. This correlated with the expected ER stress and autophagosome formation whereas expression of wild type SEC16B rescued the collagen trafficking defect. Despite the intriguing evidence, the presence of a single patient makes it difficult to unequivocally substantiate SEC16B as a causative gene for osteogenesis imperfecta. The authors are strongly encouraged to confirm the OI phenotype in an animal model and identify more families in support of this causative relation.

Referee #3 (Comments on Novelty/Model System for Author):

Overall, this is a convincing study, which potentially identifies a new gene, in which pathogenic variants cause osteogenesis imperfecta. The major weakness is that only one patient is described and that the genetic testing did not include the whole family. Nevertheless, if the specific issues listed in my comments to the authors can be addressed, a revised version of the manuscript may be acceptable.

Referee #3 (Remarks for Author):

Based on the identification of a homozygous SEC16B missense mutation in a child with osteogenesis imperfecta, the authors performed various cell culture experiments to demonstrate the necessity of SEC16B for protein trafficking and type I collagen processing. Overall, this is a convincing study, which identifies a novel candidate gene, in which pathogenic variants can cause osteogenesis imperfecta. There are however several issues that remain to be addressed by the authors.

Specific comments:
1) Since the authors refer to the link of SEC16B variants with obesity, it would be informative to provide the BMI of the patient. Another question is, if serum/urine parameters of bone remodeling and/or calcium homeostasis were determined. If so, these data should be presented.
2) In general, the patient description can be improved, especially with respect to timeline, number and location of fractures and treatment efficacy. It may be worthwhile to include a Figure or Table with BMD and height parameters over time, since it is indeed quite remarkable that BMD was normalized at the age of 18 years. The statement regarding persistence of muscle weakness additionally raises the question, if specific parameters of muscle function were also quantified.
3) In the section "Identification of a SEC16B bi-allelic variant" the authors refer to "sequencing of the known genes associated with OI". The underlying methodology (Sanger or panel sequencing?) should be explained in the Methods section. Moreover, since there was no trio approach applied for whole exome sequencing, the authors have to clarify if they also identified bi-allelic pathogenic variants in other genes. Finally, while the Results section states that two other siblings were not available for genetic testing, Figure 3 actually shows three other siblings.
4) Figure 5A/B and Figure 5C/D should be exchanged in order to follow the text in the Results section. There is also an unusually lengthy description of the RUSH assay, which should either be shortened or be part of the Methods or Discussion

section.
5) That the SEC16B mutation does not affect protein function, but mRNA abundance is surprising and raises some questions. In this regard the authors should at least speculate, how such an influence is possibly explained, and they should certainly comment on the possibility that splicing events may be affected. Moreover, since the SEC16B gene contains 26 exons, they have to clarify, also by using additional qRT-PCR primers, if specific fragments of the mRNA are underrepresented in patient fibroblasts.
6) Another finding, which is only briefly described and not really discussed, is related to the quite interesting findings obtained by mass spectrometry shown as Supplementary Table 1. Here there should be some additional statements and/or illustrations about the potential specificity of the observed fragments. Especially the relevance of the patient-specific peptide (residues 985-994) has to be discussed at one point. Otherwise, these data can also be omitted.
7) Since Figure 4B and Figure 7C are apparently based on the same methodology, there is a need to explain, why the presented values are 50-fold higher in patients cells transfected with empty vector.

Please find below a point-by-point response to the reviewer's comments.

Response to comments and suggestions

We thank the referees for their insightful comments that helped to improve and shape our manuscript. Here, we provide a point-by-point response to the reviewer' comments, and changes in the revised manuscript are highlighted in the text.

Referee #1

"Comments: This interesting and well-conducted study appears to finally shed light on the function of the SEC16B protein. The conclusion is that in cell with a high secretory demand, expression levels of SEC16B are similar to those of SEC16A and the former is important for the ER to GOLGI trafficking of large cargo proteins, such as type I collagen. Therefore, SEC16B mutations affect collagen trafficking and cause a skeletal dysplasia reminiscent of a recessive form of OI. I have the following specific comments:"

Response: We thank the reviewer for her/his supportive comment.

1. TITLE. Since the authors have identified only 1 patient in 1 family with this genetic mutation, since the underlying skeletal dysplasia shows some unique features such as vertebral compression fractures only and no fractures in long bones, muscle hypotonia, and histological findings of mineralized cartilage remnants which are less typical of OI, and the authors themselves (in the Discussion - line 369) suggest SEC16B as a new candidate gene for recessive OI, I would recommend to change the title into something less affirmative and more suggestive of a potential new candidate gene for OI. After all, a second patient with mutations in SEC16B may present with a different phenotype, so I feel it is a bit premature to state that SEC16B is a new OI gene.

Response: We agree with the reviewer and have therefore changed the title as follows: "Bi-allelic mutation in SEC16B alters collagen trafficking and increases ER stress" (p.1 lines 1-2).

In addition, we have changed the running title to "SEC16B, a recessive candidate gene for OI"

2. Abstract: same comment as above for the final sentence (lines 76-77). Also, there is a typo in the verb (cause).

Response: This following change has been made: "These data suggest SEC16B as a recessive candidate gene for OI." (p.4 line 74)

3. Introduction: here the authors properly state in the last sentence that 'SEC16B is a candidate gene in OI'.

Response: We followed the suggestion of the reviewer and used this wording in the abstract and title.

4. Results: line 214 - it appears from Fig. 4 C,D that primarily COL1A1 is retained in the ER and much less COL1A2. Perhaps normalization to PDI levels (instead of calnexin) in Figure D would provide a better evidence for the concomitant retention of COL1A2?

Response: We would like to keep the normalization to calnexin for two reasons. Firstly, the expression levels of PDI are quite variable between experiments and therefore, normalization to PDI would only increase the variance in our data. The second reason is that we show that cells lacking SEC16B experience ER stress, thus explaining why the levels of calnexin and BiP are higher. We think it is more accurate to account for this biological fact and therefore normalize to an ER chaperone that is changing in the expected way.

5. Lines 220-221: the statement that SEC16B was proposed to exert its function at ERES....needs references to support it.

Response: we have added two references form the Glick and Stephens labs showing that SEC16B localizes to ERES, which implies that this is its primary site of action. (p.11 line 226-227, references)

"Bhattacharyya D, and Glick BS. Two mammalian Sec16 homologues have nonredundant functions in endoplasmic reticulum (ER) export and transitional ER organization. Mol Biol Cell. 2007;18(3):839-49."

"Budnik A, Heesom KJ, and Stephens DJ. Characterization of human Sec16B: indications of specialized, non-redundant functions. Sci Rep. 2011;1:77."

6. Lines 229-236: I am confused about the mass spectrometric findings from the secreted collagen. How is it possible that only certain peptides were detected in one genotype and not in the other one? How do the authors explain this? I could understand the lack of a post-translational modification (e.g. a phosphorylation) on one peptide in the patient vs the control collagen but how to explain the lack of entire peptides? Was the collagen fragmentation incomplete? This was rather confusing and the statement in the discussion that collagen from the patient cells was differentially modified is not supported by the data as is. Please explain.

Response: For mass spectrometry (MS), soluble proteins in the cell medium supernatant, which were retained by 100 kDa MWCO ultrafiltration, were analyzed. Thus, we excluded that endogenous fragments of COL1A1 were detected by MS. Eventually, detected peptides were obtained by digestion of the ultrafiltrate with trypsin. Differences in peptide detection might be due to cleavage sites being resistant or inaccessible to trypsin digestion due to post-translational modifications such as acetylation or glycation (Guan and Xiong,Trends Biochem Sci 2011; Wang et al., JBC 2002: Rýglová and Suchý, Macromol Mater Eng 2017).

Specificity: Only peptide identifications by MS and MS/MS (peptide mass and masses of collision-induced peptide fragmentation) with high confidence (Mascot Ion Score >= 40) and detected in replicates were considered as differences (Peptide tolerance was set to 10 ppm and the MS/MS tolerance to 0.05 Da). Still, the full dataset including all Mascot Ion Scores for COL1A1 is presented. Thus, we conclude that both phosphorylation as well as changing resistance to trypsin digestion are part of the molecular phenotype of the SEC16B mutant. For clarification, we added a short explanation to the results and discussion.

RESULTS: p.11, lines 242-244

"…Absence of specific peptides in all replicates at high confidence suggests that flanking cleavage sites were resistant or inaccessible to digestion by trypsin."

Discussion p17., lines 385-390

"…Furthermore, several COL1A1 peptides were either detectable only in the SEC16B wildtype or mutant background. Differences in detected peptides indicate that flanking cleavage sites were resistant or inaccessible to trypsin digestion. Posttranslational modifications, such as acetylation or glycation, interfere with digestion by trypsin (Guan & Xiong, 2011, Rýglov., Braun et al., 2017, Wang, Xu et al., 2002) …"

Discussion p19, lines 439-441
"…In addition, our mass spectrometry analysis showed secretion of aberrant forms of COL1A1 including the absence of the serine 1193 phosphorylation site in the patient."

7. Line 314: please keep using past tense when discussing the results (we were unable to).

Response: We followed the suggestion of the reviewer and now use past tense throughout the manuscript.

8. Line 321: the finding of a 70% reduction of SEC16B mRNA in the patient's fibroblasts is quite interesting but there is no discussion on how this could happen. After all this is a missense mutation, so how could the mutant protein affect the transcription of its own coding gene? There should be some discussion about how this could happen.

Response: We agree that this surprising finding requires more discussion which we now include in the revised manuscript. All our attempts to show that the mutation itself affects protein function were negative (localization and dynamics on ERES). We added new data showing that the interaction of mutant and wild type SEC16B with SEC16A is not altered, which further strengthens our original conclusion that the mutation itself does not have a major effect on protein function (p.15, line 319-321, EV Figure 1*D*, with methods line 644-649).

As to why the levels of SEC16B mRNA are reduced, we wanted to respond to the comment of the reviewer by investigating whether mutant and wild type cells exhibit different splicing and exon usage. We used primers covering the entire open reading frame of SEC16B from the control and the patient cells. To stabilize mRNAs, we treated with cycloheximide. We did not detect any appreciable alterations, which indicates that there is no cryptic splicing site, intron retention, exon skipping or deletion. (New EV Figure 2) Uncovering the mechanism for this is not a trivial task, and there might be multiple mechanisms. These include alterations of the structure of the mRNA which could affect its stability or translation. Another possibility is the codon usage that might affect the stability of the expression or stability of the Sec16B mRNA. Finally, we might envisage a scenario where the mutation affects a yet unknown cellular function of SEC16B that affects its mRNA expression or stability. We added this discussion on p.15, line 328-339 and EV Figure 2 (with methods lines 566-573).

9. Line 330: I would not say that the decrease was 'marked' as it appears mild from the provided figure 8 D,E.

Response: we have omitted "marked" from the sentence. (p. 15, line 348).

10. If there is a collagen trafficking defects, is there ultimately a reduced amount of collagen outside the cells? A simple collagen staining of the ECM deposed by these fibroblasts could provide an answer to this. Also, a collagen preparation from the media of these cells could show a delayed electrophoretic migration indicating indeed altered PTMs. Were these experiments performed?

Response: The trafficking defect is most likely not restricted to collagen. It is possible that the trafficking of other proteins such Golgi enzymes might also be affected, which would result in defects in post-translational modifications. Therefore, the skeletal defect might be caused by a combination of a defect in collagen trafficking defect and/or its post-translational modification. To analyze this systematically is a subject for future investigations and is outside the scope of our current work.

11. Line 371: there is a typo - 'which has the closest homology to...'

Response: Thank you. We have corrected it. (p. 18, line 397)

12. Final statement - Lines 410-414: I would say that 'the mutation results in lower SEC16B mRNA levels...' and fix the statement on COL1A1 PTM since these were not demonstrated.

Response: We have made the requested changes (p. 19, line 437).

"… mutation results in lower SEC16B mRNA levels…-"

And p.19, line 439-441
"…. In addition, our mass spectrometry analysis showed secretion of aberrant forms of COL1A1 such as the absence of serine 1193 phosphorylation site in the patient…"

13. Table 1: please indicate what the numbers in brackets are. Are these SD or else?

Response: We thank the Reviewer for pointing out this oversight. Data are mean ± SD or median [25th; 75th percentiles]. In the revised version we have added the corresponding information below table 1 (Now p.35 line 789)

14. Legend to Figure 5: Quantification of the number of ERES per area from cells processed as in panel E. Panel E from what figure?

Response: We are very sorry for this error. Panel C is meant.

Referee #2:
The authors present an exciting case of a homozygous SEC16B mutation as cause for osteogenesis imperfecta. In line with the involvement of SEC16B in anterograde transport, the functional analyses showed trafficking defects including accumulation of collagen in the ER. This correlated with the expected ER stress and autophagosome formation whereas expression of wild type SEC16B rescued the collagen trafficking defect. Despite the intriguing evidence, the presence of a single patient makes it difficult to unequivocally substantiate SEC16B as a causative gene for osteogenesis imperfecta. The authors are strongly encouraged to confirm the OI phenotype in an animal model and identify more families in support of this causative relation.

Response: In the lack of a second affected family, it is understandable that the reviewer asks for an animal model. As outlined above, we think that an animal model would not be directly useful. The reasons for deciding against an animal model are as follows: (i) the mutated residue (R142) is not conserved in mice, and thus it is not possible to perform a transgenic mouse model; (ii) a knockout model is unlikely to yield meaningful results, because the patient cells do not lack SEC16B completely. Therefore, an animal model would require us to test different degrees of a lack of SEC16B (e.g. 50-90% depletion), which is not feasible within a scope of a revision. (iii) A model in non-mammals such as zebrafish are also potentially problematic with respect to the comparability with the mammalian ER-export system. Deficiency of Sec24D in zebrafish causes a bone phenotype, but the knockout in mouse is embryonically lethal. Altogether, performing animal experiments in this context appears unfeasible and therefore we decided to tone down the message of our work, as suggested by the editor.

Referee #3

Based on the identification of a homozygous SEC16B missense mutation in a child with osteogenesis imperfecta, the authors performed various cell culture experiments to demonstrate the necessity of SEC16B for protein trafficking and type I collagen processing. Overall, this is a convincing study, which identifies a novel candidate gene, in which pathogenic variants can cause osteogenesis imperfecta. There are however several issues that remain to be addressed by the authors.

Specific comments:
1) Since the authors refer to the link of SEC16B variants with obesity, it would be informative to provide the BMI of the patient. Another question is, if serum/urine parameters of bone remodeling and/or calcium homeostasis were determined. If so, these data should be presented.

Response: The weight (63.3kg) and BMI (26.17kg/m^2) of the patient as a young adult has been added (p.8, line 151). Routine assessment of calcium-phosphate metabolism was repeatedly normal, as expected in OI. Bone turnover markers were not measured (p. 7, line 139-140).

2) In general, the patient description can be improved, especially with respect to timeline, number and location of fractures and treatment efficacy. It may be worthwhile to include a Figure or Table with BMD and height parameters over time, since it is indeed quite remarkable that BMD was normalized at the age of 18 years. The statement regarding persistence of muscle weakness additionally raises the question, if specific parameters of muscle function were also quantified.

Response: Fractures: As documented in the text, the boy only had vertebral fractures which were first detected age 9y, and this information is given in one sentence including location and cannot be shortened. There is no timeline for fractures.
Bone density: The BMD Z-scores values were not size corrected until age 9 (dates back to the year 2007) so technically cannot be compared to those size-corrected ones taken after age 9y. We provided the BMAD and TBLH z-score values before BP therapy and have now added those at age 18y in the text. (p. 8, line 150)
Height: We are now also providing z-scores for height now at given time points (p. 8, line 151). Providing a table, growth chart or figure for bone density and height would overload this paper which is focused on mechanism of disease. Here are the available data on height and bone density over time, we are not including this table in the manuscript.

age	4.5y	8.6y	10y (pre-BP)	18y
Height z	-2.1 (93,5cm)	-2.6 (115,2cm)		-3.77 (155,5cm)
BMAD z			-2.8	-0.9
TBLH BMD z			-1.8	-1.1

Muscle: In terms of muscle assessment, unfortunately neither CK levels, nor EMG or muscle biopsy were conducted.

3) In the section "Identification of a SEC16B bi-allelic variant" the authors refer to "sequencing of the known genes associated with OI". The underlying methodology (Sanger or panel sequencing?) should be explained in the Methods section. Moreover, since there was no trio approach applied for whole exome sequencing, the authors have to clarify if they also identified bi-allelic pathogenic variants in other genes. Finally, while the Results section states that two other siblings were not available for genetic testing, Figure 3 actually shows three other siblings.

Response: As stated in the methods section (line 487+), whole exome sequencing was conducted. Analysis focused initially on known OI genes and the subsequent search for new variants then focused on those located in coding regions (i.e. nonsense, frameshift, and missense), splicing sites and untranslated regions and having low allele frequency (<0.005) in gnomAD and our in-house exome databases". Trio (exome) analysis was not possible since the father had died but mother and one of the 2 tested brothers were heterozygous of the mutations. As for other bi-allelic variants, of course there were many given the consanguinity. Clinical bioinformatics WES output assessment produced lists with predictive scores for recessive and dominant variants of which SEC16B was identified as the most likely target gene.
Thank you for noting the mistake on the number of siblings which we have corrected now (three other siblings, p. 10, line 207).

4) Figure 5A/B and Figure 5C/D should be exchanged in order to follow the text in the Results section. There is also an unusually lengthy description of the RUSH assay, which should either be shortened or be part of the Methods or Discussion section.

Response: We have exchanged the panel A/B and C/D to better match the text. We shorted the description of the RUSH assay, but kept it partially. The reason for this is that our work is likely to be read not only by colleagues from the field of membrane trafficking, but also by clinical/translational bone researchers, who might not be familiar with this method. Keeping a short description improves the clarity of the text. (p. 31 line 706-709)

5) That the SEC16B mutation does not affect protein function, but mRNA abundance is surprising and raises some questions. In this regard the authors should at least speculate, how such an influence is possibly explained, and they should certainly comment on the possibility that splicing events may be affected. Moreover, since the SEC16B gene contains 26 exons, they have to clarify, also by using additional qRT-PCR primers, if specific fragments of the mRNA are underrepresented in patient fibroblasts.

Response: We added a speculation about the reasons for mRNA downregulation on p.15 line 328-339. Please see our response to referee #1, point 8.

6) Another finding, which is only briefly described and not really discussed, is related to the quite interesting findings obtained by mass spectrometry shown as Supplementary

Table 1. Here there should be some additional statements and/or illustrations about the potential specificity of the observed fragments. Especially the relevance of the patient-specific peptide (residues 985-994) has to be discussed at one point. Otherwise, these data can also be omitted.

Please see our response to referee #1, point 6.

7) Since Figure 4B and Figure 7C are apparently based on the same methodology, there is a need to explain, why the presented values are 50-fold higher in patients cells transfected with empty vector.

Response: In Figure 4B, we compared the healthy control cells with SEC16B mutant cells. In Figure 7C, we compared the patient cells that either expressed the empty vector or WT SEC16B. So, we are talking about two different systems with different cell types and genotypes. Also, expression of WT SEC16B in the rescue experiment (Fig. 7C) is under the control of a strong constitutive promoter (pCMV promoter), which ectopically expresses SEC16B at high levels. Thus, quantitative expression is not comparable between these two systems, since ectopic expression is much higher than endogenous expression of a specific gene. Therefore, this explains the differences between Figure 4B and Figure 7C.

<u>Specific new sections/tables/figures in the manuscript</u>

Line 566-573: **the methodology for EV Figure 2**

Line 644-649: **the methodology for EV Figure 1C**

Figure 6D and 6G, which show the quantification of the western blot: We found an error in the calculation. The figures were updated. Differences remained statistically significant and our findings remained unchanged.

EV Table 3: **the primer sequences used for Supplementary Figure 2**

EV Figure 1A, B and C: we have provided a much higher quality figures.

EV Figure 1D: **show that the interaction of mutant and wild type SEC16B with SEC16A was not altered.**

EV Figure 2: **show there is no cryptic splicing site, intron retention, exon skipping or deletion in SEC16B.**

1st Feb 2023

Dear Prof. Högler,

Thank you for the submission of your revised manuscript to EMBO Molecular Medicine. We have now heard back from the two referees who agreed to re-evaluate your manuscript. As you will see from the report below, while the referee #1 is supporting publication of the manuscript raising only a minor concern, the referee #3 appreciates your efforts in addressing the referees' comments but also raises serious concern regarding the genetic analysis.

Given the differential referee reports I have sought external advice on the study from an expert in the field. Our advisor reached a conclusion that the work presents exciting new information of inherited bone disease, but he/she also raised similar concerns as the referee #3. Therefore, in this final round of revision following points need to be addressed for further consideration of your manuscript at EMBO Molecular Medicine:

- Specify the relationship among the consanguineous parents.
- Pathogenicity of the SEC16B missense mutation should be estimated using appropriate online tools.
- It is not clear how the qPCR results were quantified. What was the reference mRNA to which SEC16B mRNA levels were normalized? Please provide more information or repeat the experiment with an appropriate control mRNA as a reference.
- The complete list of homozygous pathogenic or likely pathogenic variants in other genes identified in the patient should be published as a Data Set file.

Acceptance or rejection of the manuscript will depend on the completeness of your responses included in the next, final version of the manuscript. For this reason, and to save you from any frustrations in the end, I would strongly advise against returning an incomplete revision.

In addition, please amend the following:
1) Figures:
- Please add scale bars to figures 5A and C, 8A and D, and EV1A. Also, indicate merged images in figures 5A and C, 8A and D.
- Provide figure titles for all EV figures and a legend for the figure EV3.
- Figure 4F shows western blot quantification on n=2. Please show raw values from both measurements and remove error bars. When n=2 statistical analysis is not recommended and a justification for the use of the statistical test employed has to be provided. Please check our Author Guidelines:
https://www.embopress.org/page/journal/17574684/authorguide#statisticalanalysis
2) In the main manuscript file, please do the following:
- Correct/answer the track changes suggested by our data editors by working from the attached document.
- Limit keywords to max. 5.
- Remove text highlight color.
- Add callouts for figure EV3.
- In M&M, the statistical paragraph should reflect all information that you have filled in the Authors Checklist, especially regarding randomization, blinding, replication.
- In M&M, please include statement provided in the "Checklist" that the informed consent was obtained from the human subject and that the experiments conformed to the principles set out in the WMA Declaration of Helsinki and the Department of Health and Human Services Belmont Report.
- Please rename "Competing Interest" to "Disclosure Statement & Competing Interests" and move it after the "Acknowledgements". We updated our journal's competing interests policy in January 2022 and request authors to consider both actual and perceived competing interests. Please review the policy https://www.embopress.org/competing-interests and update your competing interests if necessary.
- Author contributions: Please remove it from the manuscript and specify author contributions in our submission system. CRediT has replaced the traditional author contributions section because it offers a systematic machine-readable author contributions format that allows for more effective research assessment. You are encouraged to use the free text boxes beneath each contributing author's name to add specific details on the author's contribution. More information is available in our guide to authors:
https://www.embopress.org/page/journal/17574684/authorguide#authorshipguidelines
- In the reference list, where there are more than 10 authors on a paper, 10 will be listed, followed by "et al.". Please check "Author Guidelines" for more information.
https://www.embopress.org/page/journal/17574684/authorguide#referencesformat
- Raw data from large-scale datasets (WES) should be deposited in one of the relevant databases and made freely available prior the publication of the manuscript. Use the following format to report the accession number of your data:

The datasets produced in this study are available in the following databases:
[data type]: [full name of the resource] [accession number/identifier] ([doi or URL or identifiers.org/DATABASE:ACCESSION])

Please check "Author Guidelines" for more information.
https://www.embopress.org/page/journal/17574684/authorguide#availabilityofpublishedmaterial
- Please remove EV tables from the manuscript and uploud them as separate files. Also, correct nomenclature to Table EV1 etc. and Figure EV1 etc.
3) The Paper Explained: Please provide "The Paper Explained" and add it to the main manuscript text. Please check "Author Guidelines" for more information. https://www.embopress.org/page/journal/17574684/authorguide#researcharticleguide
4) Synopsis: Every published paper now includes a 'Synopsis' to further enhance discoverability. Synopses are displayed on the journal webpage and are freely accessible to all readers. They include separate synopsis image and synopsis text.
- Synopsis image: Please provide the visual abstract as a high-resolution jpeg file 550 px-wide x (250-400)-px high.
- Synopsis text: Please provide a short standfirst (maximum of 300 characters, including space) as well as 2-5 one sentence bullet points that summarise the paper as a .doc file. Please write the bullet points to summarise the key NEW findings. They should be designed to be complementary to the abstract - i.e. not repeat the same text. We encourage inclusion of key acronyms and quantitative information (maximum of 30 words / bullet point). Please use the passive voice.
- Please check your synopsis text and image before submission with your revised manuscript. Please be aware that in the proof stage minor corrections only are allowed (e.g., typos).
5) For more information: This space should be used to list relevant web links for further consultation by our readers. Could you identify some relevant ones and provide such information as well? Some examples are patient associations, relevant databases, OMIM/proteins/genes links, author's websites, etc...
6) As part of the EMBO Publications transparent editorial process initiative (see our Editorial at http://embomolmed.embopress.org/content/2/9/329), EMBO Molecular Medicine will publish online a Review Process File (RPF) to accompany accepted manuscripts. This file will be published in conjunction with your paper and will include the anonymous referee reports, your point-by-point response and all pertinent correspondence relating to the manuscript. Let us know whether you agree with the publication of the RPF and as here, if you want to remove or not any figures from it prior to publication. Please note that the Authors checklist will be published at the end of the RPF.
7) Please provide a point-by-point letter INCLUDING my comments as well as the reviewer's reports and your detailed responses (as Word file).

I look forward to reading a new revised version of your manuscript as soon as possible.

Yours sincerely,

Zeljko Durdevic

Zeljko Durdevic
Editor
EMBO Molecular Medicine

***** Reviewer's comments *****

Referee #1 (Comments on Novelty/Model System for Author):

The authors did not use any animal model and justified that in their rebuttal. The in vitro cell models that they used were aedquate.

Referee #1 (Remarks for Author):

I am overall satisfied by the author's replies to my comments and I think that the manuscript has improved substantially. I am only asking them to clarify the data on the ERES: they state that the number of ERES was slightly but consistently elevated in patient fibroblasts (Lines 230-232 and Figure 5B). Then later in the results (lines 300-303) they affirm that the presence of Sec16b-R142W mutation does not affect the number of ERES - which I found confusing. I have no further comments.

Referee #3 (Remarks for Author):

The authors have addressed most of my questions, yet the most critical issue remains the genetic analysis. First (a minor issue), if only WES was conducted, the statement "After sequencing of the known genes associated with OI (El-Gazzar & Hogler, 2021) failed to identify causative variants, whole exome sequencing was performed ..." is certainly incorrect. Second, if, indeed as expected, many other bi-allelic variants were detected, the genetic analysis of only two unaffected family members, both heterozygous for the mutation, by Sanger sequencing may not be sufficient, especially since only a single patient is described here. Therefore, it is certainly correct that the authors now changed the title of their manuscript and only refer to a candidate gene for recessive OI.

Please find below a point-by-point response to the second round of the Editor and reviewer's comments.

Response to comments and suggestions

We thank the Editor and referees for their insightful comments that helped to improve and shape our revised manuscript. Here, we provide a point-by-point response to the Editor and reviewer' comments.

Editor's comments

"- Specify the relationship among the consanguineous parents."

Response: Parents are first cousins. This was added (line 148)

"- Pathogenicity of the SEC16B missense mutation should be estimated using appropriate online tools."

Response: We performed whole exome sequencing in 2016. As expected for a consanguineous individual, we got a large number of homozygous variants in different genes (see Table EV1). The rationale to select this variant from the list of candidates in 2016 was that it was a rare variant, which was predicted to be damaging by the prediction programs used at that time (SIFT and Polyphen2). In addition, it was possible to construct a link with collagen trafficking because SEC16B interacts with SEC31 that is in complex with TANGO1, a protein well known to regulate collagen secretion.

Other in silico pathogenicity prediction programs we employed recently in addition to the original SIFT and Polyphen2 estimations do, by majority not reach the same estimations. Such prediction tools are without any doubt helpful in many conditions. However, different tools reach different conclusions, none of them is perfect and finally functional assays have to be performed in order to show the relevance of genetic variants. This we have done. Our data demonstrate the clear cellular phenotype that we can attribute to SEC16B. That cellular phenotype fits very well with the bone phenotype observed clinically. Thus, overall these prediction tools are of reduced value for the paper.

“- It is not clear how the qPCR results were quantified. What was the reference mRNA to which SEC16B mRNA levels were normalized? Please provide more information or repeat the experiment with an appropriate control mRNA as a reference.”

Response: In figure 8 B and C, the qPCR data are displayed as fold of control. The results were calculated as ΔΔCT using actin as internal reference transcript. ΔΔCT was calculated as delta CT of the SEC16B - delta CT of the control as in figure 8B. To work out the fold gene expression we performed 2^-delta delta CT. This was added (line 578-582)

“- The complete list of homozygous pathogenic or likely pathogenic variants in other genes identified in the patient should be published as a Data Set file.”

Response: Please find the list attached. Please see Table EV1.

<u>Referee 1</u>

"I am overall satisfied by the author's replies to my comments and I think that the manuscript has improved substantially. I am only asking them to clarify the data on the ERES: they state that the number of ERES was slightly but consistently elevated in patient fibroblasts (Lines 230-232 and Figure 5B). Then later in the results (lines 300-303) they affirm that the presence of Sec16b-R142W mutation does not affect the number of ERES - which I found confusing. I have no further comments."

Response: We thank the reviewer for positive evaluation of our manuscript. As for the effect on ERES number: we already indicated in lines 271-272 that the tiny increase in ERES number is most likely due to the UPR induction. Such an effect is most likely biologically insignificant. This is why we mentioned later that there is no significant effect on ERES number. We have made the following change in the revised manuscript (line 320-322) *"…. In addition, we noticed above that the presence of SEC16B-R142W does not affect the number of ERES significantly"*

<u>Referee #3:</u>

"The authors have addressed most of my questions, yet the most critical issue remains the genetic analysis. First (a minor issue), if only WES was conducted, the statement "After sequencing of the known genes associated with OI (El-Gazzar & Hogler, 2021) failed to identify causative variants, whole exome sequencing was performed ..." is certainly incorrect. Second, if, indeed as expected, many other bi-allelic variants were detected, the genetic analysis of only two unaffected family members, both heterozygous for the mutation, by Sanger sequencing may not be sufficient, especially since only a single patient is described here. Therefore, it is certainly correct that the authors now changed the title of their manuscript and only refer to a candidate gene for recessive OI."

Response: Thank you for the precious feedback. As suggested by the referee, we omitted the first part of the sentence. It now reads *"Whole exome sequencing was performed and identified a homozygous missense variant…"* (line 223). **For your**

second point, we are glad that the referee agrees to the change of title and message.

We are pleased to inform you that your manuscript is accepted for publication and is now being sent to our publisher to be included in the next available issue of EMBO Molecular Medicine.

EMBO Press Author Checklist

Corresponding Author Name: Prof. Wolfgang Högler	
Journal Submitted to: EMBO Molecular Medicine	
Manuscript Number: EMM-2022-16834-V2	

USEFUL LINKS FOR COMPLETING THIS FORM
The EMBO Journal - Author Guidelines
EMBO Reports - Author Guidelines
Molecular Systems Biology - Author Guidelines
EMBO Molecular Medicine - Author Guidelines

Reporting Checklist for Life Science Articles (updated January 2022)
This checklist is adapted from Materials Design Analysis Reporting (MDAR) Checklist for Authors. MDAR establishes a minimum set of requirements in transparent reporting in the life sciences (see Statement of Task: 10.31222/osf.io/9sm4x). Please follow the journal's guidelines in preparing your manuscript.
Please note that a copy of this checklist will be published alongside your article.

Abridged guidelines for figures
1. Data
The data shown in figures should satisfy the following conditions:
- the data were obtained and processed according to the field's best practice and are presented to reflect the results of the experiments in an accurate and unbiased manner.
- ideally, figure panels should include only measurements that are directly comparable to each other and obtained with the same assay.
- plots include clearly labeled error bars for independent experiments and sample sizes. Unless justified, error bars should not be shown for technical replicates.
- if n<5, the individual data points from each experiment should be plotted. Any statistical test employed should be justified.
- Source Data should be included to report the data underlying figures according to the guidelines set out in the authorship guidelines on Data Presentation.

2. Captions
Each figure caption should contain the following information, for each panel where they are relevant:
- a specification of the experimental system investigated (eg cell line, species name).
- the assay(s) and method(s) used to carry out the reported observations and measurements.
- an explicit mention of the biological and chemical entity(ies) that are being measured.
- an explicit mention of the biological and chemical entity(ies) that are altered/varied/perturbed in a controlled manner.
- the exact sample size (n) for each experimental group/condition, given as a number, not a range;
- a description of the sample collection allowing the reader to understand whether the samples represent technical or biological replicates (including how many animals, litters, cultures, etc.).
- a statement of how many times the experiment shown was independently replicated in the laboratory.
- definitions of statistical methods and measures:
  - common tests, such as t-test (please specify whether paired vs. unpaired), simple χ2 tests, Wilcoxon and Mann-Whitney tests, can be unambiguously identified by name only, but more complex techniques should be described in the methods section;
  - are tests one-sided or two-sided?
  - are there adjustments for multiple comparisons?
  - exact statistical test results, e.g., P values = x but not P values < x;
  - definition of 'center values' as median or average;
  - definition of error bars as s.d. or s.e.m.

Please complete ALL of the questions below.
Select "Not Applicable" only when the requested information is not relevant for your study.

Materials

Newly Created Materials	Information included in the manuscript?	In which section is the information available? (Reagents and Tools Table, Materials and Methods, Figures, Data Availability Section)
New materials and reagents need to be available; do any restrictions apply?	Not Applicable	

Antibodies	Information included in the manuscript?	In which section is the information available? (Reagents and Tools Table, Materials and Methods, Figures, Data Availability Section)
For **antibodies** provide the following information:<br>- Commercial antibodies: RRID (if possible) or supplier name, catalogue number and or/clone number<br>- Non-commercial: RRID or citation	Yes	Material and Methods

DNA and RNA sequences	Information included in the manuscript?	In which section is the information available? (Reagents and Tools Table, Materials and Methods, Figures, Data Availability Section)
**Short novel DNA or RNA including primers, probes**: provide the sequences.	Yes	Expanded View Table 3

Cell materials	Information included in the manuscript?	In which section is the information available? (Reagents and Tools Table, Materials and Methods, Figures, Data Availability Section)
**Cell lines:** Provide species information, strain. Provide accession number in repository **OR** supplier name, catalog number, clone number, and/**OR** RRID.	Not Applicable	
**Primary cultures:** Provide species, strain, sex of origin, genetic modification status.	Yes	Material and Methods and Figure Legends
Report if the cell lines were recently **authenticated** (e.g., by STR profiling) and tested for mycoplasma contamination.	Not Applicable	

Experimental animals	Information included in the manuscript?	In which section is the information available? (Reagents and Tools Table, Materials and Methods, Figures, Data Availability Section)
**Laboratory animals or Model organisms:** Provide species, strain, sex, age, genetic modification status. Provide accession number in repository **OR** supplier name, catalog number, clone number, **OR** RRID.	Not Applicable	
**Animal observed in or captured from the field:** Provide species, sex, and age where possible.	Not Applicable	
Please detail **housing and husbandry conditions**.	Not Applicable	

Plants and microbes	Information included in the manuscript?	In which section is the information available? (Reagents and Tools Table, Materials and Methods, Figures, Data Availability Section)
**Plants:** provide species and strain, ecotype and cultivar where relevant, unique accession number if available, and source (including location for collected wild specimens).	Not Applicable	
**Microbes:** provide species and strain, unique accession number if available, and source.	Not Applicable	

Human research participants	Information included in the manuscript?	In which section is the information available? (Reagents and Tools Table, Materials and Methods, Figures, Data Availability Section)
If collected and within the bounds of privacy constraints report on age, sex and gender or ethnicity for all study participants.	Yes	Results and Material and Methods

Core facilities	Information included in the manuscript?	In which section is the information available? (Reagents and Tools Table, Materials and Methods, Figures, Data Availability Section)
If your work benefited from core facilities, was their service mentioned in the acknowledgments section?	Yes	Acknowledgments

Design

Study protocol	Information included in the manuscript?	In which section is the information available? (Reagents and Tools Table, Materials and Methods, Figures, Data Availability Section)
If study protocol has been **pre-registered, provide DOI in the manuscript**. For clinical trials, provide the trial registration number **OR** cite DOI.	Not Applicable	
Report the **clinical trial registration number** (at ClinicalTrials.gov or equivalent), where applicable.	Not Applicable	

Laboratory protocol	Information included in the manuscript?	In which section is the information available? (Reagents and Tools Table, Materials and Methods, Figures, Data Availability Section)
Provide DOI OR other citation details if **external detailed step-by-step protocols** are available.	Not Applicable	

Experimental study design and statistics	Information included in the manuscript?	In which section is the information available? (Reagents and Tools Table, Materials and Methods, Figures, Data Availability Section)
Include a statement about **sample size** estimate even if no statistical methods were used.	Yes	Material and Methods
Were any steps taken to minimize the effects of subjective bias when allocating animals/samples to treatment (e.g. **randomization procedure**)? If yes, have they been described?	Not Applicable	
Include a statement about **blinding** even if no blinding was done.	Not Applicable	
Describe **inclusion/exclusion criteria** if samples or animals were excluded from the analysis. Were the criteria pre-established?<br><br>If sample or data points were omitted from analysis, report if this was due to attrition or intentional exclusion and provide justification.	Not Applicable	
For every figure, are **statistical tests** justified as appropriate? Do the data meet the assumptions of the tests (e.g., normal distribution)? Describe any methods used to assess it. Is there an estimate of variation within each group of data? Is the variance similar between the groups that are being statistically compared?	Yes	Material and Methods, and Figure Legends

Sample definition and in-laboratory replication	Information included in the manuscript?	In which section is the information available? (Reagents and Tools Table, Materials and Methods, Figures, Data Availability Section)
In the figure legends: state number of times the experiment was **replicated** in laboratory.	Yes	Figure Legends
In the figure legends: define whether data describe **technical or biological replicates**.	Yes	Figure Legends

Ethics

Ethics	Information included in the manuscript?	In which section is the information available? (Reagents and Tools Table, Materials and Methods, Figures, Data Availability Section)
Studies involving **human participants**: State details of **authority granting ethics approval** (IRB or equivalent committee(s), provide reference number for approval.	Yes	Material and Methods
Studies involving **human participants**: Include a statement confirming that **informed consent** was obtained from all subjects and that the experiments conformed to the principles set out in the WMA Declaration of Helsinki and the Department of Health and Human Services Belmont Report.	Yes	Material and Methods
Studies involving **human participants:** For publication of **patient photos**, include a statement confirming that consent to publish was obtained.	Not Applicable	
Studies involving experimental **animals**: State details of **authority granting ethics approval** (IRB or equivalent committee(s), provide reference number for approval. Include a statement of compliance with ethical regulations.	Not Applicable	
Studies involving **specimen and field samples:** State if relevant **permits** obtained, provide details of authority approving study; if none were required, explain why.	Not Applicable	

Dual Use Research of Concern (DURC)	Information included in the manuscript?	In which section is the information available? (Reagents and Tools Table, Materials and Methods, Figures, Data Availability Section)
Could your study fall under dual use research restrictions? Please check biosecurity documents and list of **select agents and toxins** (CDC): https://www.selectagents.gov/sat/list.htm	Not Applicable	
If you used a select agent, is the security level of the lab appropriate and reported in the manuscript?	Not Applicable	
If a study is subject to dual use research of concern regulations, is the name of the **authority granting approval and reference number** for the regulatory approval provided in the manuscript?	Not Applicable	

Reporting

The MDAR framework recommends adoption of discipline-specific guidelines, established and endorsed through community initiatives. Journals have their own policy about requiring specific guidelines and recommendations to complement MDAR.

Adherence to community standards	Information included in the manuscript?	In which section is the information available? (Reagents and Tools Table, Materials and Methods, Figures, Data Availability Section)
State if relevant guidelines or checklists (e.g., **ICMJE, MIBBI, ARRIVE, PRISMA**) have been followed or provided.	Not Applicable	
For **tumor marker prognostic studies,** we recommend that you follow the **REMARK** reporting guidelines (see link list at top right). See author guidelines, under 'Reporting Guidelines'. Please confirm you have followed these guidelines.	Not Applicable	
For **phase II and III randomized controlled trials**, please refer to the **CONSORT** flow diagram (see link list at top right) and submit the CONSORT checklist (see link list at top right) with your submission. See author guidelines, under 'Reporting Guidelines'. Please confirm you have submitted this list.	Not Applicable	

Data Availability

Data availability	Information included in the manuscript?	In which section is the information available? (Reagents and Tools Table, Materials and Methods, Figures, Data Availability Section)
Have **primary datasets** been deposited according to the journal's guidelines (see 'Data Deposition' section) and the respective accession numbers provided in the Data Availability Section?	Not Applicable	
Were **human clinical and genomic datasets** deposited in a public access-controlled repository in accordance to ethical obligations to the patients and to the applicable consent agreement?	Not Applicable	
Are **computational models** that are central and integral to a study available without restrictions in a machine-readable form? Were the relevant accession numbers or links provided?	Not Applicable	
If publicly available data were reused, provide the respective **data citations in the reference list**.	Not Applicable	
